# Perioperative Depression and Anxiety Care in Older Patients: A Randomized Clinical Trial

**DOI:** 10.1001/jamanetworkopen.2026.32619

**Published:** 2026-09-09

**Authors:** Joanna Abraham, Katherine J. Holzer, Simon Haroutounian, Bethany Pennington, Ana Baumann, Ryan Calfee, Theresa Cordner, Kenneth E. Freedland, Thomas Kannampallil, Benjamin Kozower, Emily Lenard, J Philip Miller, Mary Politi, Michael Yingling, Michael S. Avidan, Eric J. Lenze

**Affiliations:** 1 Department of Anesthesiology, Washington University School of Medicine (WashU Medicine), St Louis, Missouri; 2 Institute for Informatics, Data Science, and Biostatistics, WashU Medicine, St Louis, Missouri; 3 Department of Surgery, School of Medicine, WashU Medicine, St Louis, Missouri; 4 Department of Orthopedics, School of Medicine, WashU Medicine, St Louis, Missouri; 5 Department of Psychiatry, School of Medicine, WashU Medicine, St Louis, Missouri; 6 Bursky School of Public Health, Washington University, St Louis, Missouri

## Abstract

**Importance:**

Depression and anxiety are common among older adults undergoing surgery and are associated with adverse postoperative outcomes. However, effective tailored perioperative mental health interventions are lacking.

**Objective:**

To evaluate a perioperative intervention to optimize mental health.

**Design, Setting, and Participants:**

A single-blind, hybrid, type 1, effectiveness-implementation randomized clinical trial was conducted (November 1, 2022, to March 31, 2025), with 3-month postoperative follow-up, at a US academic and community practice hospital network. Participants were 60 years or older; scheduled for cardiac, oncologic, or orthopedic surgery; and had clinically meaningful symptoms of depression and/or anxiety based on the Patient Health Questionnaire–Anxiety and Depressive Symptom (PHQ-ADS) scale. A total of 3159 patients were screened for eligibility, with 1518 ineligible, 1079 declining participation, and 236 excluded for other reasons. A total of 326 patients were enrolled and randomized (1:1), with 20 excluded after surgery cancelation.

**Intervention:**

Participants were assigned to receive a perioperative intervention combining psychological management and pharmacologic optimization or enhanced usual care (materials for self-managing symptoms).

**Main Outcomes and Measures:**

The primary outcome was change in PHQ-ADS score from baseline to 3 months after surgery. Other outcomes included persistent postsurgical pain, delirium, falls, quality of life, patient satisfaction, length of stay, and rehospitalizations. Implementability was evaluated through semistructured interviews and reach, acceptability, feasibility, appropriateness, and fidelity measures.

**Results:**

A total of 306 older adults were included in analysis (mean [SD] age, 68.5 [6.1] years; 209 [68.3%] female; 153 randomized to intervention and 153 randomized to enhanced usual care): 102 cardiac, 100 oncologic, and 104 orthopedic patients. Participants’ mean (SD) baseline PHQ-ADS score was 18.5 (7.4). At 3 months, there was a significant decrease in PHQ-ADS scores in the intervention group compared with the enhanced usual care group (mean difference, 2.20; 95% CI, 0.16-4.24; *P* = .03). Effects varied by surgical subgroups (oncologic patients: mean difference, 4.93; 95% CI, 1.51-8.36; *P* = .005; cardiac patients: mean difference, 2.68; 95% CI, −0.98 to 6.35; *P* = .15; and orthopedic patients: mean difference, −1.11; 95% CI, −4.62 to 2.40; *P* = .54). Patients and interventionists perceived the intervention as appropriate, with high-fidelity delivery and broad reach across the target population.

**Conclusions and Relevance:**

In this randomized clinical trial, psychological management and pharmacologic optimization reduced anxiety and depression in older adults undergoing surgery. Future studies should assess reproducibility and determine which patients benefit most.

**Trial Registration:**

ClinicalTrials.gov Identifiers: NCT05575128, NCT05685511, and NCT05697835

## Key Points

**Question:**

Does an intervention combining psychological management and pharmacologic optimization improve depression and anxiety in older adults undergoing major surgery?

**Findings:**

In this randomized clinical trial of 306 participants, a perioperative psychological and pharmacologic intervention significantly improved depression and anxiety symptoms at 3 months postoperatively compared with enhanced usual care.

**Meaning:**

The findings provide initial evidence that psychological management and pharmacologic optimization can improve mental health among older adults in the perioperative period.

## Teaser

This randomized clinical trial of older surgical populations evaluates the effectiveness and implementation potential of a perioperative intervention to reduce anxiety and depression symptoms.

## Introduction

Depression and anxiety symptoms are common in older surgical patients, affecting an estimated 10% to 33% and 22% of patients preoperatively, respectively, in the US^1,2^ and 6% to 52% for depression and 5% to 45% for anxiety globally.^3^ These symptoms co-occur with medication use, such as sedatives-hypnotics or antipsychotics,^4,5^ and are associated with higher risks of postoperative complications, including delirium, falls, delayed recovery, and persistent postsurgical pain.^6,7,8,9,10^

Although treatments to decrease anxiety and depression for surgical patients exist,^11,12^ current perioperative care models do not adequately address mental health needs and challenges faced by older adults.^5,13^ To address this, we codeveloped and systematically adapted a perioperative intervention to optimize mental health for older surgical patients using a community-based participatory research approach^14^ with input from multiple stakeholders, including older adults with lived surgical experiences.^15,16,17^ It combined psychological management (eg, behavioral activation and other psychotherapeutic strategies)^18^ with pharmacologic optimization (eg, deprescribing potentially inappropriate central nervous system–active medications and optimizing antidepressant doses).^16^ This article reports findings from 3 linked randomized clinical trials (RCTs) conducted among cardiac, oncologic, and orthopedic older surgical populations evaluating the effectiveness and implementation potential of a perioperative intervention to reduce anxiety and depression symptoms.

## Methods

### Setting and Participants

The study was conducted within an academic and community practice hospital network serving rural, suburban, and urban populations (see trial protocol in Supplement 1). Each trial, conducted in cardiac, oncologic, and orthopedic surgical populations, was approved by the Washington University School of Medicine (WashU) Medicine Institutional Review Board.^19^ Written informed consent was obtained from participants.

Participants were identified using procedure schedules and direct clinician referrals. There was no minimum required interval between enrollment plus randomization and the index operation. Initial screening used the Patient Health Questionnaire 4 (PHQ-4), assessing depression and anxiety symptoms,^20^ with scores of 3 or higher considered as a positive screen result, indicating at least mild symptoms. Individuals with a positive screen result were referred for full screening using the 16-item Patient Health Questionnaire–Anxiety and Depressive Symptom (PHQ-ADS) scale, which combines the 9-item PHQ (PHQ-9) and 7-item Generalized Anxiety Disorder (GAD-7) questionnaires.^21^

Between November 1, 2022, and March 31, 2025, a total of 3159 patients were screened for eligibility, and 326 patients were enrolled and randomized (1:1), with 20 excluded after surgery cancelation. Participants were included if they were 60 years or older; scheduled for orthopedic, oncologic, or cardiac surgery; and had clinically meaningful anxiety and/or depression symptoms (score of ≥10 on the PHQ-ADS; range, 0-48, with 0 indicating no symptoms of anxiety or depression [ie, not at all on every PHQ-9 and GAD-7 item] and 48 indicating the maximum possible symptom burden (ie, nearly every day on every item), consistent with extremely severe anxiety and depression symptoms). Participants were excluded if they could not read, speak, or understand English, demonstrated severe cognitive impairment as assessed by a Short Blessed Test^22^ score of 10 or higher, or had active suicidal ideation as assessed by an adapted suicide safety screening.^23^ Race and ethnicity were abstracted from the patient’s electronic record and confirmed by participant self-report during screening. Race and ethnicity were collected in accordance with National Institutes of Health reporting policies to characterize the study population and assess the representativeness and generalizability of study findings.

### Randomization and Masking

Consented participants were randomized 1:1 to the intervention or control groups. Treatment assignments were performed via REDCap (Research Electronic Data Capture) randomization module using a randomized block design. For those assigned to the intervention, a randomization table with a similar block sequence design was generated, and the data manager selected and assigned an interventionist to each participant. The study team was blinded to allocation, whereas the participants and interventionists were unblinded due to the nature of the intervention.

### Study Design

We used a hybrid, type 1, effectiveness-implementation design to test the effectiveness of the intervention while examining implementation potential.^24^ A mixed-methods sequential explanatory approach integrating quantitative outcomes with qualitative data enabled interpretation of intervention effectiveness and implementation outcomes while perioperative context and participants’ lived experiences were captured.^25^ We followed the Consolidated Standards of Reporting Trials (CONSORT)^26^ guideline for reporting RCTs and the Consolidated Criteria for Reporting Qualitative Research (COREQ)^27^ guideline for reporting qualitative studies. The qualitative findings are reported in a separate publication.^28^

### Perioperative Intervention to Optimize Mental Health

The intervention included psychological management and pharmacologic (or medication) optimization (eFigure 1 in Supplement 2). Both components were rooted in principles of compassionate and patient-centered care.^29^

Psychological management was delivered by master’s-level social workers or counselors, called *wellness partners*, using a structured, manualized approach rooted in principles of behavioral activation and supportive counseling.^30^ Wellness partners elicited patients’ values, goals, and surgery-related concerns; assessed symptoms of depression, anxiety, sleep disruption, and cognitive concerns; and collaboratively developed individualized activity plans aligned with patients’ values. They provided psychological support before and after surgery while empowering patients to overcome recovery barriers, navigate the health care system, and communicate effectively with clinical teams.

Clinical pharmacists and pharmacy students, with geriatric psychiatrist oversight, led pharmacologic optimization.^31,32^ The pharmacist team reviewed and verified home medications and refill histories, developed individualized deprescribing plans for potentially inappropriate central nervous system–active medications, optimized subtherapeutic antidepressant dosing, monitored responses to medication changes, provided patient education, and coordinated recommendations with prescribing clinicians. Detailed manuals are available in eMethods 1 in Supplement 2. Weekly interventionist meetings were conducted with wellness partners and the pharmacist team to review patient cases, ensure fidelity, and collaboratively address emerging challenges.

### Intervention Delivery

The intervention was delivered across the perioperative period (eFigure 1 in Supplement 2), with sessions scheduled according to patient preferences. Psychological management typically began preoperatively, with 1 to 2 sessions every 2 weeks. After surgery, psychological management continued for 2 to 12 sessions or approximately 3 months. Pharmacologic optimization was initiated preoperatively before hospital admission and continued throughout the postoperative period, with medication changes actively monitored and reinforced after discharge for up to 3 months. Sessions were delivered via telephone.

### Enhanced Usual Care

Control participants received enhanced usual care (EUC), which included written materials (paper handout or email) on “mindfulness and recovery from surgery,” “self-help for disrupted sleep,” and “training your brain.”^33,34,35^ These materials were selected for their relevance to older patients with anxiety and/or depression.^34,35,36,37^

### Primary Outcome

The primary outcome was change in PHQ-ADS score from baseline to 3 months postoperatively. This outcome reflects the expected timeframe for therapeutic effects of the intervention and a clinically meaningful period of postoperative recovery.

### Other Exploratory Effectiveness Outcomes

Patient-reported exploratory effectiveness outcomes included health-related quality of life,^38^ postoperative falls,^39^ and persistent postsurgical pain (pain interference^40^ and pain severity at rest and with movement). These outcomes were assessed at baseline, 1 month, and 3 months after surgery and were collected via REDCap surveys. Other exploratory outcomes, obtained from the electronic health record, included in-hospital delirium identified using the Chart-Based Delirium Identification Instrument (CHART-DEL) method,^41^ hospital length of stay, and all-cause rehospitalizations (at 1 month and 3 months).

### Other Exploratory Implementation Outcomes

Exploratory implementation outcomes included intervention reach, patient satisfaction, and the acceptability, feasibility, and appropriateness of implementation.^42^ Implementation data sources included fidelity rating checklists,^43^ biweekly intervention session forms, patient end-of-study interviews, interventionist periodic reflections, weekly case review meeting notes, and patient self-report surveys. End-of-study patient interviews guided by the Consolidated Framework for Implementation Research^44^ and interventionist reflections explored intervention adaptations, implementation challenges, and facilitators. We also measured target engagement by examining intervention patients’ perceptions of their activation,^45^ understanding of their medications, and preparedness for surgery^46^ and by assessing interventionists’ compassion toward patients.^29^ Perioperative mental health intervention implementation evaluation methods are detailed in eMethods 2 and eTable 1 in Supplement 2.

### Sample Size Calculation

The within-person minimally important difference for the PHQ-ADS is approximately 4 points.^21^ Because between-group differences in RCTs average treatment effects across responders and nonresponders, clinically meaningful between-group differences are typically smaller (approximately 1-3 points), although no consensus threshold has been established. Assuming an SD of 8.8 and a 2-sided α = .05, the pooled sample (3 cohorts × 46 participants per group) provided 94% power to detect a 4-point between-group difference and 81% power to detect a 3-point difference.

### Analysis of Intervention Delivery

Quantitative data, including intervention fidelity and intervention session characteristics, were reported using descriptive statistics. Qualitative data, including patient interviews and interventionist periodic reflections, were analyzed using inductive thematic analysis.^55^ Weekly case review notes and patient session forms were analyzed using qualitative and quantitative content analyses.^56^ Qualitative findings were used to contextualize the quantitative results, providing deeper insight into observed patterns and outcomes.

### Statistical Analysis

Analyses were conducted in a modified intention-to-treat population, including all randomized participants except those whose surgery was canceled after randomization. The prespecified statistical analysis plan is available in Supplement 1. Internal consistency (Cronbach α) was calculated for the PHQ-9 and GAD-7 at baseline and 3 months within each cohort. The primary outcome was analyzed separately for each cohort using repeated-measures mixed models with fixed effects for treatment group, time (baseline, 1 month, and 3 months), and their interaction. Least-squares mean changes from baseline to 3 months were compared between groups, with sensitivity analyses adjusting for baseline values. A prespecified, individual-patient, 2-stage, fixed-effect meta-analysis^47^ summarized treatment effects across the 3 cohorts. This is logically equivalent to a single fixed-effect model in which the study group is a fixed effect and the inference is whether there is a mean intervention effect in these 3 specific groups taken as a whole.

Other outcomes measured longitudinally were analyzed using analogous mixed models. Length of stay was analyzed using analysis of covariance; delirium, rehospitalization, and persistent postsurgical pain using modified Poisson regression with a log link; number of falls using a repeated-measures mixed model; and fall severity using a repeated measures ordinal multinomial logistic regression. For these last 2 models, the baseline value was treated as a covariate. Prespecified covariates were included where appropriate, and potential effect modification by sex was examined. Analyses were performed using SAS, version 9.4M8 (SAS Institute Inc), SPSS, version 32.0 (IBM Inc), and R, version 4.5.2 (R Foundation for Statistical Computing). The level of statistical significance was set to 5%.

All models were adjusted for sex assigned at birth, surgery type based on complexity, invasiveness, length of recovery,^48,49,50,51,52^ and American Society of Anesthesiologists physical status score, a validated measure of perioperative risk based on medical comorbidities that effectively discriminates between low- and high-risk patients.^53,54^ All pain models additionally included chronic pain and opioid use as covariates. A meta-analytical approach was used to allow the effects of covariates to differ across surgical cohorts. This approach also accommodated potential differences in residual error variance across cohorts, resulting in simpler models. It was particularly appropriate because the distributions of surgery type were not equivalent across the surgical cohorts.

## Results

**Figure 1.**
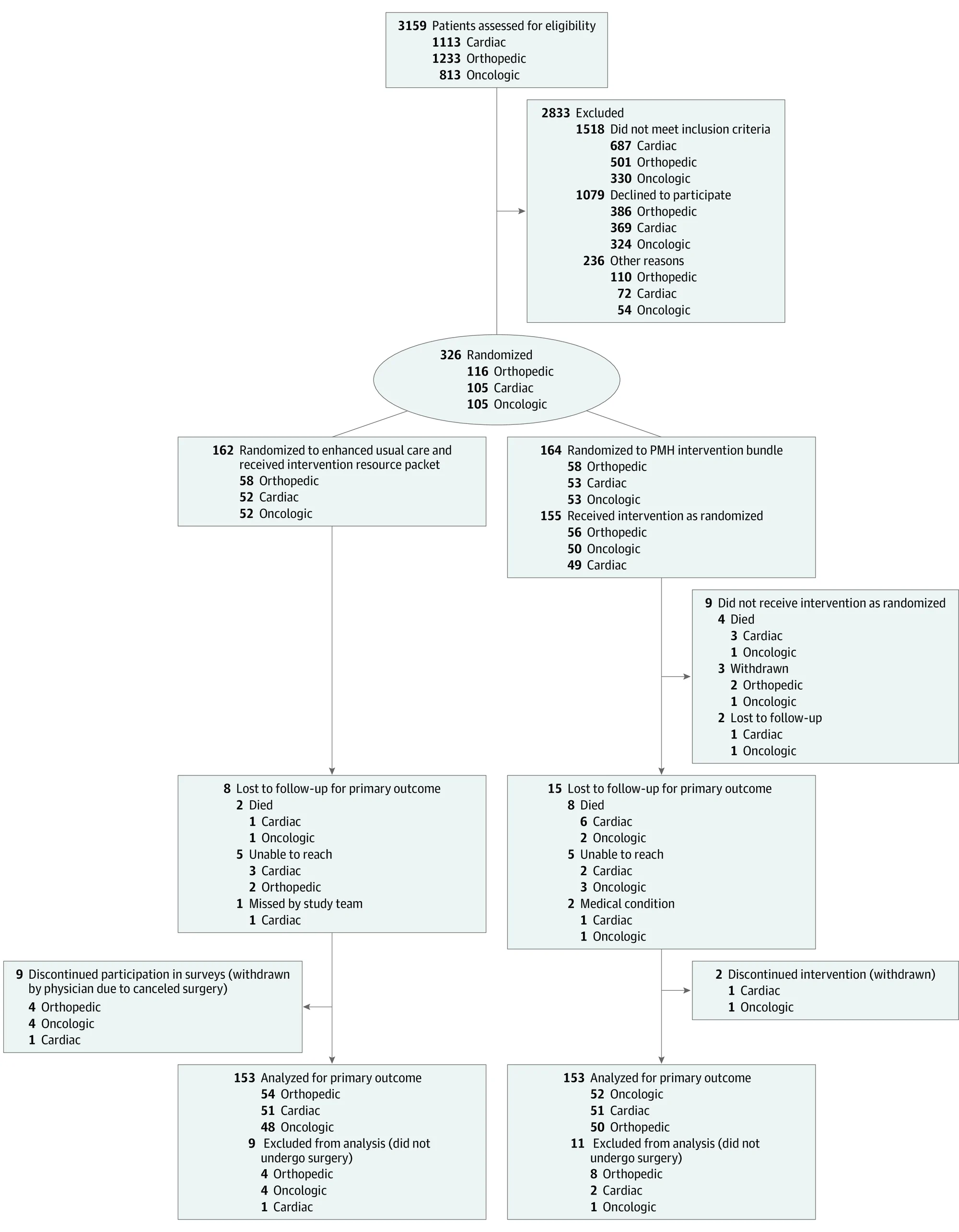
CONSORT Diagram CONSORT indicates Consolidated Standards of Reporting Trials; PMH, perioperative mental health.

Of 326 patients, 306 were analyzed (20 had their surgery canceled after randomization, with 153 randomized to intervention and 153 randomized to enhanced usual care): 209 (68.3%) were female and 97 (31.7%) were male, the mean (SD) age was 68.5 (6.1) years, and 1 (0.3%) was American Indian or Alaska Native, 45 (14.7%) were Black or African American, and 260 (85.0%) were White. The pooled enrollment rate among eligible participants was 78.6% across the 3 cohorts. Figure 1 shows the flow of participants in the study.

At baseline, participants reported mild-to-moderate levels of depression and anxiety symptoms (Table 1). All surgeries were elective procedures. Mean (SD) time from consent to surgery was 18.3 (23.5) days for the overall cohort and was similar between the intervention (18.4 [19.9] days) and usual care (18.3 [26.6] days) groups. Across surgical cohorts, the mean (SD) time from consent to surgery was 16.7 (16.1) days for cardiac patients, 11.4 (12.5) days for oncologic patients, and 26.5 (33.3) days for orthopedic participants. Cronbach α coefficients indicated acceptable internal consistency across the pooled sample and surgical cohorts (eTable 2 in Supplement 2).

**Table 1.** Baseline Participant Characteristics

Characteristic	No. (%) of participants^a^			
	Overall (N = 306)	Cardiac (n = 102)	Oncologic (n = 100)	Orthopedic (n = 104)
Treatment group				
Intervention	153 (50.0)	51 (50.0)	52 (52.0)	50 (48.1)
Usual care	153 (50.0)	51 (50.0)	48 (48.0)	54 (51.9)
Surgery type				
Open cardiac	58 (19.0)	58 (56.9)	NA	NA
Interventional cardiac	44 (14.4)	44 (43.1)	NA	NA
Breast cancer	63 (20.6)	NA	63 (63.0)	NA
Other thoracic or abdominal cancer	37 (12.1)	NA	37 (37.0)	NA
Knee replacement	61 (19.9)	NA	NA	61 (58.7)
Hip replacement	43 (14.1)	NA	NA	43 (41.3)
Age, mean (SD), y	68.5 (6.1)	69.3 (6.0)	67.3 (6.4)	68.9 (5.9)
Sex assigned at birth				
Female	209 (68.3)	52 (51.0)	84 (84.0)	73 (70.2)
Male	97 (31.7)	50 (49.0)	16 (16.0)	31 (29.8)
Race^b^				
African American or Black	45 (14.7)	18 (17.6)	15 (15.0)	12 (11.5)
American Indian or Alaska Native	1 (0.3)	1 (1.0)	0	0
White	260 (85.0)	83 (81.4)	85 (85.0)	92 (88.5)
Non-Hispanic or non-Latino ethnicity	306 (100.0)	102 (100.0)	100 (100.0)	104 (100.0)
GAD-7 score, mean (SD)^c^	8.9 (4.2)	8.7 (4.0)	9.4 (4.5)	8.5 (4.0)
PHQ-9 score, mean (SD)^d^	9.7 (4.5)	9.9 (4.4)	9.8 (5.1)	9.3 (4.1)
PHQ-ADS score, mean (SD)^e^	18.5 (7.4)	18.5 (6.9)	19.2 (8.4)	17.8 (7.0)
ASA score, mean (SD)^f^	2.9 (0.7)	3.6 (0.6)	2.7 (0.5)	2.4 (0.6)

Abbreviations: ASA, American Society of Anesthesiologists; GAD-7, 7-item Generalized Anxiety Disorder; NA, not applicable; PHQ-9, 9-item Patient Health Questionnaire; PHQ-ADS, Patient Health Questionnaire–Anxiety and Depressive Symptom.

^a^ Unless otherwise indicated.

^b^ Race and ethnicity were abstracted from the electronic health record and confirmed by participant self-report during screening.

^c^ Assessed using the GAD-7, which consists of 7 items each rated on a scale of 0 (not at all) to 3 (nearly every day); sample question: “Over the last 2 weeks, how often have you been bothered by feeling nervous, anxious or on edge?”

^d^ Assessed using the PHQ-9, which consists of 9 items each rated on a scale of 0 (not at all) to 3 (nearly every day); sample question: “Over the last 2 weeks, how often have you been bothered by having little interest or pleasure in doing things?”

^e^ Assessed using the PHQ-9 and GAD-7 (see the previous 2 footnotes).

^f^ The ASA score was used to measure a patient’s overall health on a scale of 1 to 6, with 1 indicating a normal, healthy patient and 6 indicating a patient with brain death.

### Intervention Effect on Primary Outcome

The intervention group showed a significantly greater improvement in depression and anxiety symptoms scores between baseline and 3 months postoperatively compared with the EUC groups, with a mean difference of 2.20 (95% CI, 0.16-4.24; *P* = .03). The *Q* test yielded a *P* = .05, consistent with heterogeneity in effects across the 3 surgical cohorts. These effects across surgical subtypes were, in order from largest to smallest or null, as follows: 4.93 (95% CI, 1.51-8.36; *P* = .005) for oncologic patients, 2.68 (95% CI, −0.98 to 6.35; *P* = .15) for cardiac patients, and −1.11 (95% CI, −4.62 to 2.40; *P* = .54) for orthopedic patients.

**Figure 2.**
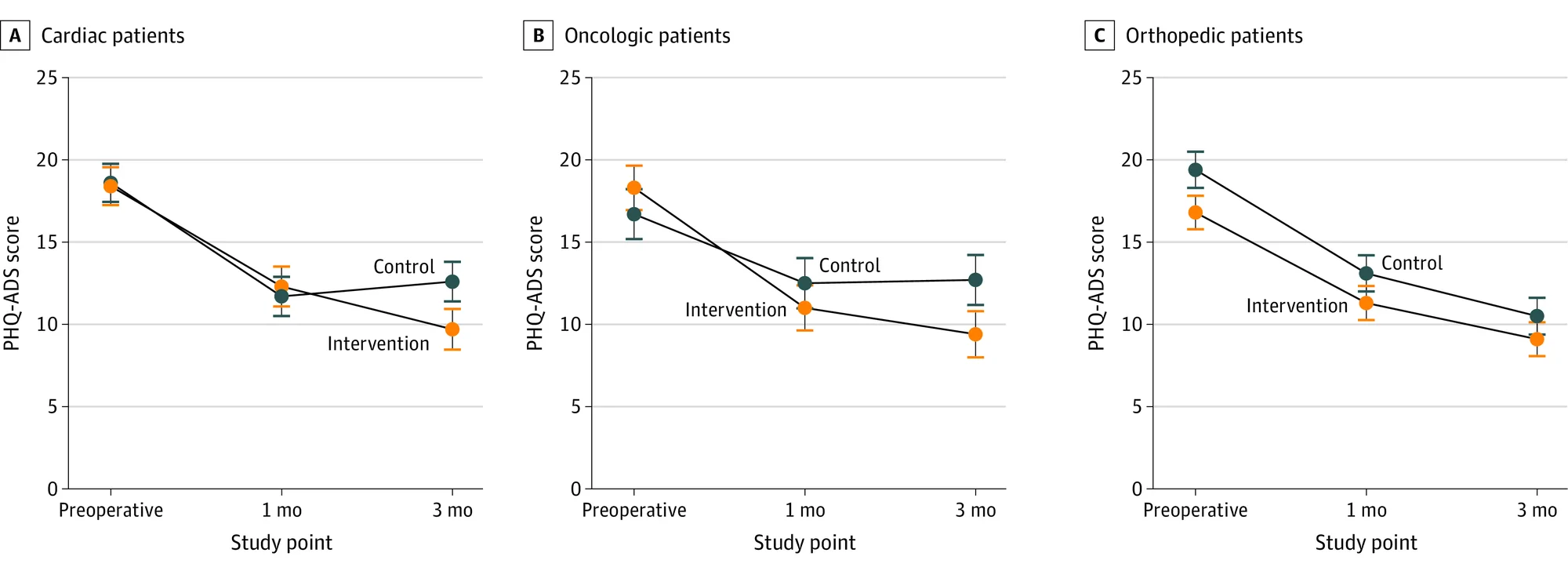
Changes in Anxiety and Depression Scores Among Surgical Cohorts PHQ-ADS indicates Patient Health Questionnaire–Anxiety and Depressive Symptom. Whiskers indicate SEs.

Figure 2 shows the course of depression and anxiety symptoms in each surgical group. Table 2 reports the effect of the intervention on depression and anxiety symptoms separately. In a sensitivity analysis adjusting for baseline PHQ-ADS score, the intervention was associated with greater reductions in PHQ-ADS scores at 3 months compared with EUC in the pooled sample (mean difference, 2.50 points; 95% CI, 0.76-4.24 points; *P* =.005). The adjusted mean differences were 2.98 points (95% CI, −0.14 to 6.09 points; *P *= .06) in the cardiac cohort, 4.84 points (95% CI, 1.96-7.71 points; *P* =.001) in the oncology cohort, and 0.34 points (95% CI, −2.53 to 3.20 points; *P* = .82) in the orthopedic cohort.

**Table 2.** Effect of the Intervention on Other Effectiveness Outcomes

Outcome	Effect size (95% CI)^a^			
	Overall meta-analysis	Cardiac patients	Oncologic patients	Orthopedic patients
Primary outcomes				
Depressive and anxiety symptoms^b^^,^^c^	2.20 (0.16-4.24)	2.68 (−0.98 to 6.35)	4.93 (1.51-8.36)	−1.11 (−4.62 to 2.40)
Depressive symptoms alone^b^^,^^d^	1.49 (0.28-2.70)	2.35 (0.26-4.45)	2.67 (0.63-4.70)	−0.80 (−2.98 to 1.38)
Anxiety symptoms alone^b^^,^^e^	0.88 (−0.19 to 1.95)	0.32 (−1.73 to 2.37)	2.31 (0.60-4.01)	−0.32 (−2.16 to 1.52)
In-hospital outcomes				
Length of stay, % of EUC (95% CI)^f^	110 (90-120)	100 (70-130)	120 (90-170)	110 (90-130)
Delirium, ARR (95% CI) (n = 42 events)^g^	1.05 (0.68-1.61)	1.14 (0.72-1.79)	0.45 (0.09-2.21)	0.76 (0.08-6.92)
Postsurgical outcomes				
Readmission, ARR (95% CI) (n = 41 events)^g^	0.68 (0.38-1.24)	0.58 (0.26-1.30)	0.98 (0.33-2.89)	0.59 (0.13-2.75)
Health-related quality of life, No. of healthy days (95% CI)^b^^,^^h^	−1.49 (−3.00 to 0.03)	−2.84 (−5.82 to 0.14)	−2.76 (−5.08 to −0.44)	1.31 (−1.37 to 3.99)
Pain severity at rest^i^				
Month 1^j^	−0.79 (−1.38 to −0.21)	−0.84 (−1.86 to 0.19)	−0.96 (−2.03 to 0.13)	−0.63 (−1.57 to 0.32)
Month 3^k^	−0.34 (−0.84 to 0.16)	−0.53 (−1.34 to 0.28)	−0.48 (−1.45 to 0.49)	−0.05 (−0.88 to 0.79)
Pain severity with movement^l^				
Month 1^j^	−0.95 (−1.64 to −0.26)	−0.52 (−1.62 to 0.59)	−1.26 (−2.54 to 0.02)	−1.18 (−2.39 to 0.02)
Month 3^k^	−0.38 (−0.97 to 0.20)	−0.30 (−1.14 to 0.54)	−0.58 (−1.70 to 0.54)	−0.33 (−1.51 to 0.84)
Persistent postsurgical pain, ARR (95% CI)^g^^,^^m^				
Month 1 (n = 149 events)^j^	0.78 (0.62-0.97)	0.76 (0.44-1.31)	0.71 (0.49-1.04)	0.83 (0.60, 1.15)
Month 3 (n = 81 events)^k^	0.73 (0.50-1.06)	0.65 (0.29-1.48)	0.69 (0.36-0.76)	0.80 (0.46-0.71)
Pain interference^n^				
Month 1^j^	−0.72 (−1.28 to −0.15)	−0.39 (−1.38 to 0.60)	−0.77 (−1.68 to 0.15)	−1.03 (−2.09 to 0.03)
Month 3^k^	−0.41 (−0.85 to 0.03)	−0.36 (−0.99 to 0.28)	−0.52 (−1.41 to 0.38)	−0.40 (−1.22 to 0.41)
No. of postsurgical falls				
Month 1^j^	−0.05 (−0.15 to 0.05)	−0.17 (−0.40 to 0.06)	−0.03 (−0.17 to 0.11)	−0.002 (−0.19 to 0.19)
Month 3^k^	−0.03 (−0.10 to 0.05)	0.005 (−0.15 to 0.15)	0.04 (−0.12 to 0.19)	−0.07 (−0.17 to 0.03)

Abbreviations: ARR, adjusted relative risk; EUC, enhanced usual care.

^a^ Unless otherwise indicated.

^b^ The contrast of interest is the change in the intervention group score (score at month 3 subtracted from the score at month zero) minus the change in the EUC group score (calculated the same way).

^c^ Assessed using the 9-item Patient Health Questionnaire (PHQ-9) and 7-item Generalized Anxiety Disorder (GAD-7) scales (see footnotes c and d).

^d^ Assessed using the PHQ-9, which consists of 9 items each rated on a scale of 0 (not at all) to 3 (nearly every day); sample question: “Over the 2 weeks, how often have you been bothered by having little interest or pleasure in doing things?”

^e^ Assessed using the GAD-7, which consists of 7 items each rated on a scale of 0 (not at all) to 3 (nearly every day); sample question: “Over the last 2 weeks, how often have you been bothered by feeling nervous, anxious or on edge?”

^f^ The contrast of interest is the length of stay for the EUC group subtracted from the length of stay for the intervention group.

^g^ Reference group was EUC.

^h^ Assessed using the Health-Related Quality of Life Scale, which consists of 5 items; sample question: “During the past 30 days, for about how many days have you felt very healthy and full of energy?”

^i^ One item measured on a scale of 0 (no pain) to 10 (worst pain); sample question: “During the past week, what was your average pain level while you were at rest?”

^j^ The contrast of interest is the intervention group score at month 1 minus the EUC group score at month 1.

^k^ The contrast of interest is the intervention group score at month 3 minus the EUC group score at month 3.

^l^ One item measured on a scale of 0 (no pain) to 10 (worst pain); sample question: “During the past week, what was your average pain level while you were active or moving?”

^m^ Persistent postsurgical pain defined as having pain at the site of surgery in the past week, with pain intensity while at rest or while moving of at least a 3 on a scale of 0 (no pain) to 10 (worst pain). The contrast of interest is the intervention group score minus the EUC group score at month 1 and at month 3, separately; values supplied are ARRs with 95% CIs.

^n^ Seven items of the Brief Pain Inventory measured on a scale of 0 (did not interfere) to 10 (completely interfered); sample question: “During the past week, how much did the pain in your surgical area interfere with your general activity?”

### Intervention Effect on Exploratory Outcomes

The intervention was associated with a lower rate of clinically meaningful persistent postsurgical pain at 1 month, as well as statistically significant reductions in pain severity at rest, with movement, and interference at 1 month. The intervention was not associated with a significant rate of improved postoperative health-related quality of life (ie, number of healthy days) and did not alter in-hospital outcomes, including length of stay, odds ratio of postoperative delirium, or rates of postoperative rehospitalizations and falls (Table 2). Additional details are available in eTables 3 to 7 in Supplement 2.

### Intervention Effect on Implementation Outcomes

Table 3 summarizes the implementation outcomes. Patients reported positive experiences with the intervention during perioperative care, with respect to acceptability, appropriateness, and feasibility. They also expressed high levels of satisfaction with their interactions with wellness partners and the pharmacist team interventionists. Additional details on intervention implementation outcomes and patient satisfaction findings across cohorts are available in eFigures 2 to 4 and eTables 8 to 11 in Supplement 2. No trial-related adverse events occurred.

**Table 3.** Intervention Implementation: Potential Outcomes and Target Engagement

Outcome	Mean (SD)^a^
Psychological management	
Reach, No./total No. (%) of participants who completed ≥7 sessions	114/146 (78.1)
Acceptability^b^	4.4 (0.5)
Appropriateness^c^	4.4 (0.6)
Feasibility^d^	4.4 (0.5)
Fidelity, %	
Treatment delivery^e^	98.8
Treatment receipt^f^	99.2
Total sessions	7.4 (2.8)
Preoperative sessions	1.2 (1.4)
Postoperative sessions	6.2 (2.4)
Session duration, min	42.4 (17.6)
Pharmacologic optimization	
Reach, No./total No. (%) of patients who accepted and implemented the recommendations from the pharmacist team	77/87 (88.5)
Acceptability^b^	4.3 (0.9)
Appropriateness^c^	4.3 (0.8)
Feasibility^d^	4.3 (0.7)
Fidelity, %	
Treatment delivery^e^	78.2
Treatment receipt^f^	71.9
Total sessions	2.2 (1.5)
Preoperative sessions	1.2 (0.7)
Postoperative sessions	1.8 (1.3)
Session duration, min	17.3 (9.6)
Target engagement (mechanisms of the intervention)	
Patient activation^g^	4.2 (0.6)
Patient understanding of medications^h^	3.6 (1.6)
Patient preparedness for surgery^i^	4.5 (0.5)
Patient assessment of interventionist compassion^j^	4.8 (0.5)

^a^ Unless otherwise indicated.

^b^ Four items measured on a scale of 1 (completely disagree) to 5 (completely agree); sample question: “The behavioral change (or medication review and changes of targeted medications) component meets my approval.”

^c^ Four items measured on a scale of 1 (completely disagree) to 5 (completely agree); sample question: “Behavioral change (or medication review and changes of targeted medications) seems fitting.”

^d^ Four items measured on a scale of 1 (completely disagree) to 5 (completely agree); sample question: “Behavioral change (or medication review and changes of targeted medications) seems implementable.”

^e^ Mean percentage completed across all measures of treatment delivery.

^f^ Mean percentage completed across all measures of treatment receipt.

^g^ Behavioral Activation for Depression Scale activation subscale consists of 7 questions measured on a scale of 1 (strongly disagree) to 5 (strongly agree); sample question: “I am content with the amount and types of things I did.”

^h^ One item (“My medications are supporting my mental health”) measured on a scale of 1 (strongly disagree) to 5 (strongly agree).

^i^ Three items measured on a scale of 1 (completely disagree) to 5 (completely agree); sample question: “My wellness partner encouraged me to discuss any fears or anxiety I had about the procedure.”

^j^ The Sinclair Compassion Questionnaire consists of 5 items measured on a scale of 1 (strongly disagree) to 5 (strongly agree); sample question: “I felt that my wellness partners were attentive to me.”

### Intervention Delivery Process

Interventionists completed a mean (SD) of 7.4 (2.8) psychological management sessions (1.2 preoperative sessions and 6.2 postoperative sessions) and 2.2 (1.5) pharmacologic optimization sessions (1.2 preoperative sessions and 1.8 postoperative sessions) per patient. For psychological management, mean item-level treatment-delivery fidelity was 98.8% across 7 indicators (60 of 60 for 6 indicators and 55 of 60 for 1 indicator), and mean item-level treatment-receipt fidelity was 99.2% across 2 indicators (59 of 60 and 60 of 60). For pharmacologic optimization, mean item-level treatment-delivery fidelity was 78.2% across 9 indicators (45 of 60, 59 of 59, 44 of 44, 26 of 37, 45 of 48, 33 of 33, 1 of 1, 14 of 45, and 2 of 6), and mean item-level treatment-receipt fidelity was 71.9% across 4 indicators (28 of 32, 1 of 1, 7 of 14, and 1 of 2). Qualitative findings indicated that most patients had positive experiences with the intervention and valued support from their wellness partners and pharmacist team. Additional findings on intervention delivery and patient and interventionist experiences are presented in eTables 12 to 17 in Supplement 2.

### Target Engagement

Participants in the intervention group reported generally high levels of behavioral activation, understanding of their medications, preparedness for surgery, and assessment of the interventionists’ compassion, all of which were hypothesized mechanisms of the intervention. Target engagement scores are presented in eTable 18 in Supplement 2.

## Discussion

Perioperative care is an important part of US health care.^57^ This study evaluated the effectiveness and implementation potential of a perioperative mental health intervention for older surgical adults with depression and anxiety. We identified 3 key findings. First, depression and anxiety symptoms decreased slightly but meaningfully at 3 months postoperatively. Second, intervention effects varied by surgical subgroup. The oncologic cohort had a significant and potentially meaningful reduction of approximately 5 points from baseline, exceeding the previously reported minimally important difference of 3 to 4 points for similar groups. The cardiac cohort had a modest, nonsignificant reduction, whereas the orthopedic cohort showed no measurable effect. Third, the intervention demonstrated strong implementation potential, with high patient satisfaction, adequate fidelity, and preliminary evidence of successful engagement of the targeted mechanisms of action.

We examined cardiac, oncologic, and orthopedic groups separately because their mental health needs and surgical trajectories may differ. Oncologic participants appeared to benefit most, an important finding given the high and persistent psychological burden associated with cancer.^58,59,60,61,62^ The reasons for the modest benefits among cardiac participants and the lack of benefit among orthopedic surgeries generally followed more predictable trajectories and structured recovery plans, which may mitigate depression and anxiety.^63^ Consistent with this assertion, both subgroups showed naturalistic reductions in symptoms, limiting the ability to discern an additional treatment effect.

These cohort-specific findings support the development of precision mental health interventions tailored to surgical diagnosis.^28^ Perioperative populations vary considerably in surgical complexity and baseline characteristics. Identifying individuals at highest risk for persistent postoperative depression and anxiety may allow interventions to be tailored to their needs, preferences, and prognostic profiles. Some participants may also have experienced transient, situational symptoms that resolved after surgery without treatment.^64^ Further research is needed to understand which patients need treatment and which intervention components (including number of sessions) are most associated with symptom reduction.

Participants reported overwhelmingly positive experiences, suggesting that the intervention functioned as intended, increased behavioral activation, and improved understanding of their medications. Qualitative findings further supported the intervention’s effectiveness, feasibility, acceptability, and appropriateness for perioperative care.^28^ Delivery characteristics were adequate to be effective in this aging population and surgical context, with an appropriate session dose and high fidelity to the treatment model. The intervention was patient centered and remotely delivered by master’s-level social workers and counselors, with pharmacist-led medication optimization and psychiatrist supervision,^65^ enhancing its scalability.^66^ These features align with collaborative care and telemental health models that can extend access despite mental health specialist shortages.^67,68,69^

The intervention reduced the occurrence, severity, and interference of persistent postsurgical pain at 1 month but not at 3 months; these exploratory findings are consistent with prior studies reporting mixed effects of perioperative psychological interventions on postsurgical pain.^5,70,71,72,73^ Given the substantial burden of persistent postsurgical pain, particularly among older adults,^69,74,75^ these findings warrant further investigation. Other exploratory outcomes, such as length of stay, delirium, rehospitalization, and postoperative falls, did not significantly improve, which may reflect the elective surgical populations studied, limited statistical power, or limited intervention effects on these outcomes.

### Strengths and Limitations

Study strengths included the randomized design, inclusion of participants with clinically significant symptoms regardless of formal diagnosis, an EUC comparator reflecting usual care, and integration of quantitative and qualitative data to contextualize intervention effects and mechanisms. The collaborative planning approach, including preliminary studies and stakeholder engagement before randomized testing, further enhanced the study.^16^

This study has several limitations. First, the study was conducted within a single health care system. Second, the PHQ-ADS, although an appropriate screening instrument, may not fully distinguish transient surgery-related distress from chronic or clinically significant depression and anxiety, potentially limiting the opportunity to observe larger improvements. Third, the study was not powered to detect between-group differences in exploratory outcomes. Fourth, exclusion of participants with active suicidal ideation or moderate-to-severe cognitive impairment, along with the relatively young sample (mean age, 68.5 years), predominantly non-Hispanic White population, and exclusion of non–English speakers, may limit generalizability to higher-risk, older, more racially and ethnically diverse, and non–English-speaking populations. Additionally, the absence of an attention-matched control condition limits the ability to distinguish intervention effects from nonspecific benefits of attention, therapeutic alliance, and patient engagement. Nevertheless, this study offers critical preliminary evidence for a pragmatic, scalable intervention with the potential to transform perioperative mental health care for an increasing and clinically complex patient population.

## Conclusions

Older surgical patients are clinically complex, with multidimensional needs spanning biological, social, behavioral, and environmental factors. This study showed that a multicomponent mental health intervention can be implemented in perioperative care and result in beneficial effects. Larger studies are needed to confirm these findings.

## Notes

Supplement 1.
Trial Protocol

Supplement 2. **eFigure 1. **Perioperative mental health intervention **eMethods 1. **Perioperative mental health intervention manual of procedures (MOP) and interventionist training **eTable 1. **Intervention delivery process measures **eTable 2. **Cronbach alphas at baseline and 3 months for each surgical sub-population for depression (PHQ-9) and anxiety (GAD-7) scales **eTable 3. **Impact of intervention on health-related quality of life **eTable 4. **Incidences of delirium and all-cause readmissions **eTable 5. **Impact of intervention on persistent postsurgical pain **eTable 6. **Intervention impact on falls **eTable 7. **Impact of intervention on length of hospital stay and length of ICU stay **eFigure 2. **Intervention reach **eTable 8. **Psychological management reach details **eTable 9. **Pharmacoligical optimization reach details **eFigure 3. **Acceptability, appropriateness, and feasibility of psychological management **eFigure 4. **Acceptability, appropriateness, and feasibility of pharmacological optimization **eTable 10. **Patient satisfaction with psychological management **eTable 11. **Patient satisfaction with pharmacological optimization **eTable 12. **Intervention implementation fidelity to psychological management and pharmacological optimization **eTable 13. **Summary of planned and completed psychological management activities **eTable 14. **Summary of pharmacological interventions **eTable 15. **Medication types within pharmacological optimization sessions **eTable 16. **Intervention delivery for intervention patients **eTable 17. **Patient and interventionist experiences with the intervention **eTable 18. **Target engagement survey scores **eMethods 2.** Perioperative mental health intervention implementation evaluation methods: data collection and analysis
eReferences

Supplement 3.
Data Sharing Statement
