# Supplementary material for: Perioperative Depression and Anxiety Care in Older Patients: A Randomized Clinical Trial
Source: JAMA Netw Open. 2026 Sep 9;9(9):e2632619. doi: 10.1001/jamanetworkopen.2026.32619 (PMC13559599; doi:10.1001/jamanetworkopen.2026.32619)
Supplement: Supplementary file 1 [file jamanetwopen-e2632619-s001.pdf]

RESEARCH PROTOCOL

Behavioral Activation and Medication Optimization For Improving Perioperative Mental Health In Older Adults Undergoing Cardiac Procedures

Principal Investigator:

Simon Haroutounian, PhD
Associate Professor
Chief, Division of Clinical and Translational Research
Department of Anesthesiology
Washington University School of Medicine
660 S. Euclid Ave, Campus Box 8054
Saint Louis, MO, 63110
Tel: 314-286-1715
Fax: 314-286-2948
E-mail: sharout@wustl.edu

Co-Investigators:

Kenneth Freedland, PhD
Department of Psychiatry
Washington University School of Medicine

Michael Avidan, MBBCh
Professor and Chair, Department of Anesthesiology
Washington University School of Medicine

Eric Lenze, MD
Professor and Chair, Department of Psychiatry
Washington University School of Medicine

Joanna Abraham, PhD
Associate Professor, Department of Anesthesiology and Institute for Informatics
Washington University School of Medicine

Katherine Holzer, PhD
Assistant Professor, Department of Anesthesiology
Washington University School of Medicine

Thomas J. Graetz, MD
Associate Professor
Chief, Division of Cardiothoracic Anesthesiology
Department of Anesthesiology
Washington University School of Medicine
Medical Director, Cardiovascular Recovery (CVR) Intensive Care Unit, Missouri Baptist Medical Center

F. Muhammad Masood, MD
Associate Professor, Department of Surgery
Washington University School of Medicine

Kunal Kotkar, MD
Assistant Professor, Department of Surgery
Washington University School of Medicine

Thomas Kannampallil, PhD
Associate Professor, Department of Anesthesiology and Institute for Informatics
Washington University School of Medicine

J. Philip Miller, AB
Professor
Institute of Informatics, Data Science and Biostatistics
Washington University School of Medicine

Mary Politi, PhD
Professor, Department of Surgery
Washington University School of Medicine

Ana Baumann, PhD
Assistant Professor, Department of Surgery
Washington University School of Medicine

Bethany Pennington, PharmD
Assistant Professor,
Department of Anesthesiology
Washington University School of Medicine

Theresa Cordner
Senior Research Coordinator
Department of Anesthesiology
Washington University School of Medicine

Protocol #: 202209033

Version Date: v1 September 01, 2022
v2 October 19, 2022
v3 January 10, 2023
v4 January 30, 2023
v5 February 9, 2023

v6 March 17, 2023
v7 May 23, 2023
v8 August 15,2023
v9 October 30, 2023

1. SYNOPSIS

1. **Study Title**	Behavioral activation and medication optimization for perioperative mental health in cardiac procedures
2. **Abbreviated title**	CPMH Cardiac R34 RCT
**Objective**	Using a Hybrid Type 1 Effectiveness-Implementation RCT design, we will test the effectiveness of a bundled behavioral activation and medication optimization in reducing symptoms of depression and anxiety in older adults undergoing cardiac surgery (compared with usual care), while examining implementation outcomes.
**Study Period**	Planned enrollment duration: 12-15 weeks (an initial visit; follow-up survey at 1 and 3 months)<br><br>Planned study duration: 2 years
**Number of Patients**	100
**Study Treatment**	Behavioral activation and medication optimization
**Study Design**	Hybrid Type 1 Effectiveness-Implementation prospective randomized controlled trial design.
**Inclusion and Exclusion Criteria**	<u>Inclusion criteria</u>:<br>• Adults ≥ 60 years<br>• Scheduled major cardiac procedure<br>• PHQ-ADS ≥ 10, indicating clinically significant depression or anxiety symptoms<br><u>Exclusion criteria:</u><br>• Barrier to communication (Unable to read, speak, and understand English)<br>• Severe cognitive impairment screened by the SBT (Short Blessed Test) >10<br>• Acutely suicidal<br>• Previous participation in this study or another CPMH study of the intervention bundle or its feasibility.
**Measurements**	<u>Baseline</u><br>PHQ-ADS<br>Quality of Life (CDC HRQOL-14)<br>Short Blessed Test (SBT)<br>Presence and intensity of preoperative pain

	<u>In-Hospital</u><br>Delirium (extracted from electronic health record retrospectively)<br>Medications of interest<br><u>Postoperative</u><br>PHQ-ADS<br>Quality of life<br>Rehospitalization<br>Falls<br>Presence and intensity of Persistent post-surgical pain (PPSP)<br>Pain interference, using modified Brief Pain Inventory (BPI)<br>Medications of interest
**Statistical Methodology**	The study will use a modified Intent To Treat (mITT) approach where randomized patients who did not have the surgery or procedure will not be included in the analyses. We will compare the intra-group PHQ-ADS change scores from baseline to 3 months after surgery as the primary outcome.<br><br>As exploratory outcomes, we will compare each the following outcomes between the groups: (occurrence, or change from baseline to 1-month, and 3-month follow-ups, as appropriate):<br>• Quality of life (CDC HRQOL-14)<br>• Length of stay (ICU & hospital)<br>• Delirium<br>• Rehospitalization<br>• Falls<br>• Persistent post-surgical pain presence and pain interference<br>• Medications of interest<br><br>The following measures will be collected from patients allocated to the intervention arm for implementation assessment:<br><br>• Reach<br>• Implementation potential (i.e., Acceptability of intervention; Appropriateness of intervention; Feasibility of intervention)<br>• Patient satisfaction and experiences with overall surgical experience and intervention bundle<br><br>A combined measure including modified versions of the following scales will be collected only from patients allocated to the intervention arm to assess engagement with our intervention bundle approximately 2 months after surgery:<br>• Sinclair Compassion Questionnaire-Short Form (SCQ-SF)<br>• Behavioral Activation for Depression Scale (BADS) Activation subscale<br>• MiPrep survey

2. STUDY PROTOCOL

2.1 Background and Significance

Inadequate management of preoperative mental health disorders often contributes to poor postoperative outcomes [1], including increased rates of readmission, delirium, falls, and mortality [2-5]. However, very little work has been done to improve perioperative mental health. In particular, there have not been any systematic efforts to identify evidence-based behavioral interventions that were originally developed for depression and anxiety in otherwise medically well psychiatric patients, adapt them for use in surgical populations, test their effectiveness in these patient populations, and study implementation strategies.

Consequently, we currently lack conclusive evidence on effectiveness of interventions specifically focused on the delivery of perioperative mental health care in older surgical patients. To address this understudied research area, the proposed study will focus on comparing a bundled intervention of Behavioral Activation (BA) and Medication Optimization (MO) (compared to usual care) among older adults undergoing cardiac procedures with anesthesia.

The mental health intervention bundle, (Surgical Wellness Program) composed of BA and MO, can mitigate anxiety and depression symptoms during the perioperative period [6]. Towards this end, we propose to test the effectiveness as well as the implementation potential of the intervention bundle in a Hybrid Type 1 Effectiveness-Implementation RCT Design.

2.1.1 Preliminary Data

In an IRB-approved study (IRB # 202101103), we enrolled 8 patients undergoing cardiac surgery to an open-label feasibility phase of the study, to establish recruitment feasibility, and finalize the details pertaining to the study protocol, participant screening, informed consent process, recruitment and follow-up, as well as finalize the workflow for medication optimization and behavioral activation.

Given that these objectives were attained, and reflected in the finalized methodology proposed in this study protocol, we are ready to launch our clinical trial to evaluate the effectiveness and implementation-potential of our intervention bundle using a Hybrid Type 1 Effectiveness-Implementation RCT design.

2.2 Objective

We will test the effectiveness of a bundled intervention comprised of behavioral activation and medication optimization in reducing symptoms of depression and anxiety in older adults undergoing cardiac surgery (compared with usual care), while examining implementation outcomes. The study will be conducted by the members of the newly established Center for Perioperative Mental Health (CPMH), supported by National Institute of Mental Health (NIMH).

2.3 Patient Selection

The study coordinators will screen the Cardiac and Cardiology clinics and procedure schedules, as well as the schedule for the Center for Preoperative Assessment and Planning (CPAP) at Barnes-Jewish Hospital for patients undergoing cardiac procedures. Prospective patients will also be identified by Epic workbench reports customized to limit results to those at least 60 years of age with an eligible procedure scheduled at BJH/WUSM. Additionally, patients under the care of WashU Anesthesiology providers with eligible procedures scheduled at BJH and Missouri Baptist Medical Center (MBMC) will be included. Study collaborators in both Anesthesiology as well as in cardiac

surgery (Drs. Masood and Kotkar, along with cardiac surgery research support team) will refer patients from their clinics who meet the inclusion criteria sought from the EHR. Flyers will be available in clinical areas for potentially eligible patients at both BJH and MBMC, so interested patients may reach out to the study team for screening.

A member of the study team will obtain the patient's phone number, age and sex, as well as type of procedure, the provider’s name and planned surgery date through Epic. A member of the study team will contact potentially eligible patients to screen for inclusion criteria.

2.3.1 Inclusion Criteria

<u>Pre-screening</u>:

To be considered for participation, patients will be screened using PHQ-4 questionnaire. Scores ≥3 will indicate positive pre-screen (i.e. can be considered for participation and formal screening). Scores < 3 will indicate negative screen (i.e. patient not considered for formal screening due to low risk for anxiety or depression).

<u>Screening:</u>

Each participant must meet all of the following criteria:

Inclusion

- Adults age≥60 years
- Scheduled major cardiac procedure
- PHQ-ADS (Patient Health Questionnaire Anxiety and Depression Scale) ≥10 indicating clinically significant depression or anxiety symptoms.[7]

2.3.2 Exclusion Criteria

Participants will not be enrolled if any of the following criteria exist:

Exclusion

- Barrier to communication (Unable to read, speak, and understand English)
- Severe cognitive impairment screened by the SBT (Short Blessed Test) >10
- Acutely suicidal
- Considered ineligible per the discretion of the cardiac surgeon or study PI
- Previous participation in this study or another CPMH study of the intervention bundle or its feasibility.

2.4. Study Design

All eligible and consenting patients will be randomized in 1:1 ratio (with no stratifications) to receive either the intervention bundle or usual care.
Consenting participants (in both arms) will undergo a standard battery of assessments at baseline (prior to the intervention in the intervention group) and will be followed until approximately 3 months postoperatively. In conjunction with the effectiveness outcomes data, implementation outcomes related to reach, and implementation feasibility, acceptability, and appropriateness of the intervention bundle will be collected.

2.4.1 Study Procedures

Consented subjects will be asked to participate in the study for approximately 3 months after surgery. All participants must sign a consent document (e-consent or paper), indicating their consent, approved by the Washington University School of Medicine Institutional Review Board, before they can participate in the study.

All participants will be asked to complete the following questionnaires at baseline (in person, remotely via a web-based survey, or via telephone):

- CDC HRQOL-14 Quality of Life survey
- PHQ-ADS (Patient Health Questionnaire Anxiety and Depression Scale)
- SBT (Short Blessed Test)
- Presence and intensity of preoperative pain
- Demographics survey
- Patient-reported falls

All participants will be followed for up to approximately 3 months after their scheduled procedure. They will complete follow-up surveys at approximately 1 month and 3 months after surgery by either web-based survey, mail, or via a telephone interview.

At the end of the study, all participants in the intervention arm will be invited to take part in a semi-structured telephone interview conducted by a research team member experienced in qualitative research. The interviews will explore the participants' perceptions, attitudes, and experiences of the intervention bundle.

2.4.2 Minimization of Bias

Enrollment will not be based on sex or ethnic background. Standard of care will not be affected for any of the participants due to study participation. Completion of the outcomes will be entirely patient-reported, to minimize assessor's bias in interpreting patient responses.

2.4.3 Pre-Study Period

Patients who are ≥ 60 years of age undergoing cardiac procedures will be approached for consent to this study. Eligible patients will be invited to participate in the study, which will be conducted remotely and in-person. Potential candidates for the study will be provided a written and oral description of the study procedures, benefits, and potential risks as well as the opportunity to ask questions regarding the study. Participants are informed that their participation is voluntary and that they may refuse to participate or withdraw from the study at any time without penalty. Patients will be required to give informed consent prior to participation. A study team member who is experienced with cognitive testing will recruit the subjects to the study, and perform the SBT assessment.

Each subject who qualifies for entry into the study on the basis of inclusion/exclusion criteria and completion of informed consent will be assigned the next available patient number. This indicates enrollment in the study. Patients who are eligible but decline participation will be invited to complete an <u>optional</u> survey indicating reasons for decline.

2.4.4. Randomization

The randomization table will be generated by the study biostatistician, using a variable block sequence. The assignment of the treatment condition for each individual participant will be done by the REDCap randomization module after they have been determined to be eligible for the trial.

2.4.5. Blinding

Study staff responsible for administering ratings throughout the study are blinded. This blinding is kept throughout the study until data collection is complete. At the end of the follow-up, the study coordinator will complete a blinding question to try to guess the arm to which each participant was allocated. This will allow to determine whether the blinding procedures in the study were effective [8].

2.4.6 Study Period

Baseline Characterization of Participants: At enrollment, a battery of assessments will characterize participants. *Anxiety and depression* assessment will use the PHQ-ADS, as described above. The use of this tool for this population is supported by a recent meta-analysis suggesting that the PHQ9 (on which this tool is based) has good sensitivity and specificity in the diagnosis of depression in older adults [9]. *Quality of Life* (QoL) will be assessed using the CDC HRQOL-14 survey) [10]. The questions in this patient-reported survey correspond to the QoL domains of Healthy days (physical and mental), Activity Limitations, and Healthy Days Symptoms. *Cognitive function* assessment will be performed using the Short Blessed Test (SBT). SBT is a reliable test, with high inter-rater agreement, which is beneficial for characterizing cognitive functioning in the perioperative setting [11,12]. Recognizing the uncertainty associated with face-to-face data collection due to the COVID-19 pandemic, we selected tools such as the SBT because it can be delivered remotely [13].

All patients in both arms will receive resources (see Handouts for CPMH RCT SOC Group and Handouts for CPMH RCT Intervention Group) either by mail or email if they prefer. The resources include options for exploring brain health, supporting sleep and mental health, and introducing community resources for older adults.

Intervention Bundle: This will be initiated prior to or soon after the surgery and will be continued until approximately 3 months postoperatively.

The basic premise of *behavioral activation* is to help people with mental health problems to engage in activities that are reinforcing or meaningful and guided by their personal values [14]. Patients will be asked to check with their physicians if there is any question about the safety of any physical activities that are included in the behavioral activation plan. Behavioral activation as the core intervention allows for uniformity across participants from each cohort, yet enough flexibility for the actual components of behavioral activation to be individually adapted based on patient preferences. In a recent meta-analysis, behavioral activation programs improved depression and anxiety [15], as well as disability in older adults [16]. In addition, behavioral activation delivered by telehealth to older adults was cost-effective and lowered health care utilization costs over 1 year [17]. In addition to the core intervention of behavioral activation, study participants will tailor the intervention by choosing activities, per their preference, with demonstrated benefit in improving depression and anxiety symptoms in older adults [18,19]. Behavioral activation will generally begin pre-operatively, with sessions approximately every two weeks. After discharge, the behavioral intervention will continue, for 10-12 sessions, or out to approximately 3 months postoperatively. Patients in the intervention arm will receive information (Handouts for CPMH RCT Intervention Group) by mail or email if they prefer. The handouts include tools to support behavioral activation and optional activities.

Medications will be *reviewed and optimized*, as appropriate. In several clinical trials including thousands of older adults, we have developed and refined a process for this review and medication optimization, including adjusting suboptimal dosing for anti-depressants and discontinuing medications that are harmful for brain health (such as anticholinergics [20]). Medication optimization consists of a simple set of principles: reconcile patient's medications, identify the patient's likely need for, and interest in, a medication adjustment, make the adjustment, and assess the response to that adjustment. Benzodiazepines, and strong centrally acting anticholinergics and antihistamines, are the focus of *deprescribing*, as they increase the risk for falls and delirium. We will follow the principles of shared decision-making with patients for medication changes. While the participant is an inpatient, the study pharmacy team will coordinate with the inpatient team to ensure that medication changes that were introduced preoperatively are maintained in-house and that no new inappropriate medications are initiated. After discharge, and up to approximately 3 months postoperatively, the interventionist (also referred to as a perioperative wellness partner) and pharmacy team will ensure that targeted medication changes are reconciled during transitions of care.

Methods

2.4.6 Observations and Measurements

2.4.6.3 Primary Outcome Measures

Effectiveness outcomes will include the primary outcome measure of PHQ-ADS, a validated composite scale of depression and anxiety symptoms [7], measured at 1 and 3 months after surgery. Three months post-operatively is the primary endpoint.

Process measure for target engagement

As a process measure of target engagement at approximately 2 months after surgery, we will use a combined measure including modified versions of the Sinclair Compassion Questionnaire-Short Form (SCQ-SF) [21], Behavioral Activation for Depression Scale (BADS) Activation subscale [22,23], the MiPrep survey [24], four questions specific to our Wellness Program, and one question to assess participants' understanding of how the study's target medications can affect their mental health, if applicable.. The target engagement measure will be collected by survey, which participants in the intervention group can complete either online via REDCap link or by phone if they prefer, with an unblinded member of the study team.

2.4.6.4 Exploratory Outcome Measures

The exploratory outcomes will include QoL measured via the CDC HRQOL-14 survey, and other perioperative outcomes of interest, nominated by our stakeholders as key outcomes of interest, including:

(1) In-hospital: (i) Delirium incidence,[20], (ii) Length of Stay (hospital and ICU), (iii) information on the use of common medications for chronic pain, such as opioids, gabapentin and pregabalin.

(2) Post-discharge: (i) Falls, (ii) All-cause rehospitalization out to 90 days postoperative. (iii) persistent post-surgical pain (PPSP), (iv) information on the use of common medications for chronic pain, such as opioids, gabapentin and pregabalin.

(3) Process outcomes: For patients on the intervention arm, CAHPS modified survey will be administered after the intervention is complete.

(4) Implementation-potential surveys: Acceptability of Intervention Measure, Intervention Appropriateness Measure, Feasibility of Intervention Measure.

Table 2. Details on Study Outcomes

Effectiveness Outcomes		Data Source	Description	Data Collection Mechanism	Time points (Baseline, 1, 2, 3 month-post-op or EOS)
**Primary** ***(Clinical)***	Anxiety and Depression	Patient Health Questionnaire Anxiety and Depression Scale (PHQ-ADS)	A validated composite of symptoms of depression and anxiety	Patient Self-Report	Baseline; 1 mo and 3 mo
Exploratory (Clinical)	Quality of Life	The Health-Related Quality of Life CDC HRQOL-14 survey [10]	A patient-reported global health measure. The questions in this survey correspond to the QOL domains of Healthy days (physical and mental), Activity Limitations, and Healthy Days Symptoms	Patient Self-Report	Baseline; 1 mo and 3 mo
	In-hospital delirium incidence	Retrospective EHR chart review	.	EHR report, and Research Team Member review EHR for delirium-related information from the time of post-op inpatient stay.	In-hospital: at ICU and hospital floor
	Post-discharge falls	Patient Reported Falls	A Patent Reported Falls survey will be collected at 1 month and 3 months either by phone or REDCap link, at follow-up clinic appointments.	Patient Self-Report	Baseline; 1mo and 3mo
	Pain	Pain presence and severity at the site of surgery, and	BPI is a well validated measure of pain severity and pain	Patient self-report	Baseline, 1 mo, 3 mo

		Brief Pain Inventory (BPI) pain interference scale	interference items.		
	Length of stay (both hospital and ICU)	Research Data Core	Standardized reports on RDC warehouse will generate statistics of enrolled patients regarding the length of stay, rehospitalization, and related clinical metrics.	EHR report	In-hospital: at ICU and hospital discharge
	All-cause rehospitalization	EHR and patient		EHR, patient	Post-op: 1 mo and 3 mo
**Implementation and Process Outcomes**		**Data Source**	**Description**	**Data Collection Mechanism**	**Time points**
Reach		Enrollment Log	<u>Reach of the study</u>: patients who agreed to participate in the study out of total eligible to participate<br><u>Reach of the intervention bundle</u>: patients who completed the interventions out of patients who agreed to participate	Electronic Health Record	End of Study
Implementation-Potential (I)		Acceptability of Intervention Measure, Intervention Appropriateness Measure, Feasibility of Intervention Measure	Acceptability, Feasibility and Appropriateness 4-item Likert scale survey	Patient Self-Report	Post-EOS
Patient satisfaction and experience		Modified-Consumer Assessment of Healthcare Providers and Systems (CAHPS)[25]	The modified CAHPS asks patients about their experience with the intervention bundle before surgery, during	Patient Self-Report	Post-EOS

		surgery, and after surgery.		
**Process measure for target engagement**	**Data Source**	**Description**	**Data Collection Mechanism**	**Time points**
Target engagement measure	Combined measure of modified versions of the following scales: Sinclair Compassion Questionnaire-Short Form (SCQ-SF)[21], Behavioral Activation for Depression Scale (BADS) Activation subscale [22], the MiPrep survey[24]	The SCQ-SF is a 5-item patient-reported measure modified to assess compassion of the Wellness Provider; the BADS Activation Subscale (7 items) measures target engagement of BA in studies of geriatric depression [23]; and the MiPrep survey (3 items) assesses patients' preparation for medical interventions.	Patient Self-Report	approximately 2 mo post-op

In addition to these outcomes, we will assess fidelity to our intervention bundle to ascertain the extent to which an intervention bundle is carried out, as intended and consistently across different settings, and patients. If the patient agrees to audio/video recording, the intervention sessions will be audio recorded (or audio/video if the patient prefers to meet by Zoom teleconference). To track this, we will use the recordings of these discussions between the patient and the perioperative wellness partner during the behavioral intervention and medication optimization sessions. We will capture data on patient interactions with the wellness partner, their delivery of the intervention bundle, any observed barriers and facilitators to the intervention adherence, acceptance and fit within the perioperative workflow. 10% of these sessions of patients and their wellness partners will be selected based on a convenience sample for evaluating fidelity of the bundle.

Lastly, we will also obtain stakeholder perspectives on the intervention bundle after the intervention period has been completed using semi-structured interviews. We will conduct interviews with patients who participated in the intervention, and the topics of discussions will be guided by an implementation science framework referred to as CFIR (Consolidated Framework of Implementation Research). The interviews will explore the participants' perceptions, attitudes, and experiences with the intervention bundle, intervention bundle acceptability and detailed accounts of participants' experiences after the intervention has been stopped with regards to intervention sustainability and maintenance. Interviews will be conducted via Zoom or telephone and will be digitally recorded and transcribed verbatim.

Study Calendar

	Time 0	Baseline / Time 1	1 mo follow up	2 mo follow up	3 mo follow up	Post-EOS

Patient Consent	X					
Patient Screening	X					
Patient Baseline Surveys		X				
Patient Follow-up 1 & 3 mo Surveys			X		X	
For Patients on the Intervention Arm: Process Target Engagement Measure				X		
For Patients on the Intervention Arm: Intervention-Patient Interviews and Implementation, Patient Satisfaction and Experience surveys						X
For Patients on the Intervention Arm: Behavioral Activation and Medication Review Intervention Sessions			Approximately 8-12 sessions as needed (per Pt preference) after Baseline, continuing for up to approximately 3 months post-op.			

2.4.6.5 Statistical Methods

Analyses were conducted in a modified intention-to-treat population, including all randomized participants except those whose surgery was canceled after randomization. The prespecified statistical analysis plan is available in the Statistical Analysis Protocol (Supplement 1 below). Internal consistency (Cronbach α) was calculated for the PHQ-9 and GAD-7 at baseline and 3 months within each cohort.

The primary outcome was analyzed separately for each cohort using repeated-measures mixed models with fixed effects for treatment group, time (baseline, 1 month, and 3 months), and their interaction. Least-squares mean changes from baseline to 3 months were compared between groups, with sensitivity analyses adjusting for baseline values. A prespecified, individual-patient, 2-stage, fixed-effect meta-analysis summarized treatment effects across the three cohorts. This is logically equivalent to a single fixed-effect model in which the study group is a fixed effect and the inference is whether there is a mean intervention effect in these three specific groups taken as a whole.

Other outcomes measured longitudinally were analyzed using analogous mixed models. Length of stay was analyzed using analysis of covariance; delirium, rehospitalization, and persistent postsurgical pain using modified Poisson regression with a log link; and falls using repeated-measures mixed models adjusted for baseline. Prespecified covariates were included where appropriate, and potential effect modification by sex was examined. Analyses were performed using SAS, version 9.4M8 (SAS Institute Inc, Cary, NC, USA).

All models were adjusted for sex assigned at birth, surgery type based on complexity, invasiveness, and length of recovery, and ASA (American Society of Anesthesiologists) physical status score, a validated measure of perioperative risk based on medical comorbidities that effectively discriminates between low- and high-risk patients. All pain models additionally included chronic pain and opioid use as covariates. A meta-analytical approach was used to allow the effects of covariates to differ across surgical cohorts. This approach also accommodated potential differences in residual error variance across cohorts, resulting in simpler models. It was particularly appropriate because the distributions of surgery type were not equivalent across the surgical cohorts.

2.4.6.6 Sample Size and Power of Primary Analysis:

In order to estimate the power of the primary outcome analysis we examined the manuscript which provided the original validity and reliability studies for the PHQ-ADS [26] and one of the trials which provided data for that study (SCOPE) [27,28]. At 3 months they observed a change from baseline divided by the standard deviation of the change of 0.43 which corresponds to a change of 3 points on the PHQ-ADS. The PHQ-ADS authors estimate the minimal important clinical difference (MICD) from the standard error of measure (SEM) as 3-4 points. If we postulate a difference in the change scores of 2 points between the two groups, then with a power of 0.80 we would need 36 in each group. Since the SCOPE trial was a pain relief study without a requirement of anxiety or depression, we consider these estimates rough estimates. This trial will target having 100 mITT patients (50 per arm). In order to account for any incomplete data among recruited patients, recruitment will continue until 100 randomized patients have had their procedure or surgery.

2.5 Management of Intercurrent Events

2.5.1 Adverse Experiences

Based on the nature of this study, which involves surveys, a cognitive test, and a behavioral intervention, study related adverse events are exceedingly unlikely.

2.5.2 Premature Withdrawal

Subjects will be withdrawn if the investigator decides that discontinuation is in the best interest of the subject, or the subject requests withdrawal from the study. Additionally at PI discretion, they may be withdrawn if their surgery is cancelled or postponed (for clinical or non-clinical reasons) for more than approximately 3 months after baseline.

Participants who choose to end study participation early, may be asked to complete early termination assessments. Additionally, those in the intervention arm may choose to withdraw from that at any time, but will be allowed to continue with data collection follow-ups if they still want to participate.

2.5.3 Potential Risks

This study involves minimal risk to subjects. Unlikely but potential risks include increased risk of depression or anxiety, errors in medication recommendations or medication withdrawal symptoms, and breach of confidentiality. The risk of medication withdrawal (i.e., from benzodiazepines) is mitigated by slowly tapering rather than stopping these medications. Patients who are identified as being acutely suicidal will be excluded from the study. Nevertheless, since the rate of completed suicide in the USA remains high (i.e., about twice the rate of homicide) and most Americans who commit suicide suffer from depression, all participants eligible to participate in this study are statistically at a relatively higher risk for suicide than the general population. However, the participants' absolute risk for completing suicide during this brief study remains very low (i.e., about 1 in 3,000 to 10,000) and participation in the study does not create or increase the risk of completed suicide; rather, most experts believe that one of the most efficient ways to decrease suicidal risk in older depressed individuals is to treat their depression. Furthermore, all participants will be formally assessed frequently throughout the study. If the study personnel identify that a participant has become acutely suicidal, this participant will be referred to a mental health professional for further evaluation and treatment (see section: Procedures to Minimize Risks). This may lead to a clinical intervention that is lifesaving and may not have occurred had the participant not been participating in the study.

2.5.4 Procedures to Minimize Potential Risks

Medication Error Risk: Dr. Eric Lenze will supervise careful training and oversite of the intervention pharmacists making medication optimization recommendations to reduce the risk of medication error or medication withdrawal symptoms. Algorithms for tapering medications that should not be abruptly discontinued will be employed. We have carried out this deprescribing protocol in several large RCTs and have not seen any problems occur.

Suicide risk management: As discussed above, at each assessment point, the research staff will assess for passive death wish, and suicidal ideation, intent or plan when they administer the PHQ-ADS. If a participant endorses suicidal ideation, intent, or plan, the rater will be trained to follow an operationalized protocol that has been developed to manage high-risk participants in other studies of depressed participants potentially at risk for suicide. This protocol has already been used successfully by Dr. Lenze's team to manage acutely suicidal patients. Briefly, the protocol entails a specific determination of the suicidal risk and prescribes a set of actions. For instance, when a participant is determined to be at high and immediate risks, the rater is instructed to stay with the participant until he or she has contacted a study psychiatrist (Dr. Lenze) to discuss the situation and to devise a plan. For this reason, raters will have cell phones and Dr. Lenze will be reachable via email and cell phone. In case of extreme emergency, raters are instructed to call their hospital security team or 911 for immediate help and to initiate commitment proceedings.

Patients will be asked to check with their clinician if there is any question about the safety of any physical activities that are included in the behavioral activation plan.

The risk of breach of confidentiality will be minimized by ensuring that protected health information will only be shared with members of the research team. Only data necessary to complete the study will be collected. Electronic data will be kept in a password-protected electronic database only assessable via password protected departmental computers. Patients may choose to not participate in the study or withdraw at any time with no penalty in terms of the care they receive.

2.5.5 Data and Safety Monitoring Plan

The specific monitoring plan for this investigation is commensurate with the risks and the size and complexity of the studies planned. Given the nature of the protocol, the risks are likely limited to breach of confidentiality. The Principal Investigator will be responsible for monitoring the data and assuring protocol compliance. The PI will meet with the clinical research coordinator and Data Manager bi-weekly to review enrollment, eligibility, participant follow-up and outcome assessment. The PI and study coordinator will monitor for breaches of confidentiality on an ongoing basis. Study-related adverse events will be reported per IRB guidelines. All serious adverse events (SAE) will be recorded and adjudicated by a CPMH Principal Investigator and reported per IRB guidelines. Any endorsement of suicidal ideation will be managed acutely as detailed in the section 2.5.4. If study staff becomes aware of a reportable adverse event, it will be reported to necessary stakeholders according to Washington University Human Protection Office guidelines. This type 1 hybrid randomized study does not require a Data and Safety Monitoring Board.

3. HUMAN SUBJECTS RESEARCH

3.1 Protection of Human Subjects

The study will be conducted under appropriate Washington University Institutional Review Board protocols and consent forms approvals. The study will be conducted under the supervision of the PI, who has extensive of experience in the conduct of human research studies.

3.2 Sources of Materials

Preoperative assessments along with preoperative and postoperative questionnaires regarding quality of life, depression, and anxiety will be obtained from all study participants. The results of follow-up questionnaires will be obtained via e-mail, mail, or telephone survey. Data will be collected at baseline, one, two, and 3 months following surgical procedure.

3.2.1 List of Protected Health Information Collected for Study

The HIPAA protected identifiers that will be collected for this study include:

- Name
- Dates
  - Date of birth
  - Date of services (surgical procedure)
- Contact information
  - Mailing address
  - Phone number
  - Electronic mail address
- Medical record number

3.2.2 Data Management

All paper data will be stored under lock and key (office, file cabinet) and all electronic data will be stored in REDCap, only accessible to the research team members. Surveys that are completed electronically will be administered via the REDCap server. All REDCap email correspondence will be sent using secure survey links. All survey information will be coded with the patient's unique study ID number and no personal health information that may directly identify patients will be included.

3.3 Recruitment and Informed Consent

Participants will be recruited either from the cardiac surgery and cardiology clinics or procedure schedules at Washington University or the Center for Preoperative Assessment and Planning (CPAP) at Barnes-Jewish Hospital/Washington University. Patients under the care of WashU Anesthesiology providers undergoing eligible cardiac procedures requiring anesthesia at either BJH or MBMC will be included. Flyers will also be available in clinical areas for interested patients to contact the study team to screen. Participants with pre-screening scores of PHQ-4 ≥3 will be recruited, and eligibility confirmed if PHQ-ADS ≥10. Retention strategies will include flexibility in scheduling follow-up visits and coordination of research visits with any in-person clinical visits. Subjects will be given verbal and then written descriptions of the study aims, procedures, risks, and benefits, and will be required to give informed consent. A member of the research team will be available to present study descriptions, informed consent, and answer any questions. Subjects are informed verbally and in writing that participation is voluntary and they may refuse to participate and may withdraw from the study at any time without penalty. Remuneration for each data collection study visit will be provided to encourage retention as well.

3.4 Potential Benefits of the Proposed Research to the Subjects and Others

Although the study is designed to improve symptoms of depression and anxiety and other outcomes of care, there is no guarantee that the study participants will receive any clinical benefits by participating in the study. This may lead to a clinical intervention that is lifesaving and may not have occurred had the participant not been participating in the study. Given the undertreatment of depression and anxiety in older adults, the minimal risks are acceptable in comparison to the potential benefits in improving the mental health of older adults undergoing surgery.

3.5 Remuneration

Patients will receive remuneration by a reloadable debit card of $25 for completion of the following data collection study visits: Standard Care (control arm) participants will be compensated for their time at Baseline, 1, and 3 month post-op, up to $75 for completion of all data collection time points. Wellness Program (intervention arm) participants will also be compensated for their time at Baseline, 1, and 3 month post-op, plus a post-EOS semi-structured interview, so up to $100 for completion of all eligible data collection time points.

3.6 Inclusion of Women

Eligible individuals will be offered the opportunity to participate in the study regardless of gender or minority status. Thus, the sample composition will reflect the composition of community-living older adults in the St. Louis area.

3.7 Inclusion of Minorities

All of our studies actively encourage the participation of minorities in the research. Our minority recruiting typically matches the demographic composition of the Washington University community from which subjects will be recruited (78% white, 21% Black, <1 % Hispanic).

3.8 Inclusion of Children

We will not be including children in this study.

4. REFERENCES

1. Mangusan, R.F., et al., *Outcomes associated with postoperative delirium after cardiac surgery.* Am J Crit Care, 2015. **24**(2): p. 156-63.
2. Blumenthal, J.A., et al., *Depression as a risk factor for mortality after coronary artery bypass surgery.* Lancet, 2003. **362**(9384): p. 604-9.
3. Connerney, I., et al., *Relation between depression after coronary artery bypass surgery and 12-month outcome: a prospective study.* Lancet, 2001. **358**(9295): p. 1766-71.
4. McKenzie, L.H., J. Simpson, and M. Stewart, *A systematic review of pre-operative predictors of post-operative depression and anxiety in individuals who have undergone coronary artery bypass graft surgery.* Psychol Health Med, 2010. **15**(1): p. 74-93.
5. Takagi, H., et al., *Perioperative depression or anxiety and postoperative mortality in cardiac surgery: a systematic review and meta-analysis.* Heart Vessels, 2017. **32**(12): p. 1458-1468.
6. Abraham, J., et al., *A Qualitative Study of Perioperative Depression and Anxiety in Older Adults.* Am J Geriatr Psychiatry, 2020.
7. Kroenke, K., et al., *Patient Health Questionnaire Anxiety and Depression Scale: Initial Validation in Three Clinical Trials.* Psychosom Med, 2016. **78**(6): p. 716-27.
8. Freed, B., et al., *Assessing blinding in trials of psychiatric disorders: a meta-analysis based on blinding index.* Psychiatry Res, 2014. **219**(2): p. 241-7.
9. Levis, B., et al., *Accuracy of Patient Health Questionnaire-9 (PHQ-9) for screening to detect major depression: individual participant data meta-analysis.* BMJ, 2019. **365**: p. l1476.
10. Mielenz, T.J., L.F. Callahan, and M.C. Edwards, *Item response theory analysis of Centers for Disease Control and Prevention Health-Related Quality of Life (CDC HRQOL) items in adults with arthritis.* Health Qual Life Outcomes, 2016. **14**: p. 43.
11. Gregory, S.H., et al., *Abnormal preoperative cognitive screening in aged surgical patients: a retrospective cohort analysis.* Br J Anaesth, 2021. **126**(1): p. 230-237.
12. Oughli, H.A., et al., *Cognitive Improvement in Older Adults in the Year After Hip Fracture: Implications for Brain Resilience in Advanced Aging.* Am J Geriatr Psychiatry, 2018. **26**(11): p. 1119-1127.
13. Arias, F., et al., *Rapid In-Person Cognitive Screening in the Preoperative Setting: Test Considerations and Recommendations from the Society for Perioperative Assessment and Quality Improvement (SPAQI).* Perioper Care Oper Room Manag, 2020. **19**.
14. Puspitasari, A.J., et al., *A randomized controlled trial of an online, modular, active learning training program for behavioral activation for depression.* J Consult Clin Psychol, 2017. **85**(8): p. 814-825.
15. Stein, A.T., et al., *Looking beyond depression: a meta-analysis of the effect of behavioral activation on depression, anxiety, and activation.* Psychol Med, 2020: p. 1-14.
16. Choi, N.G., et al., *Improving Social Connectedness for Homebound Older Adults: Randomized Controlled Trial of Tele-Delivered Behavioral Activation Versus Tele-Delivered Friendly Visits.* Am J Geriatr Psychiatry, 2020.
17. Egede, L.E., et al., *Cost-Effectiveness of Behavioral Activation for Depression in Older Adult Veterans: In-Person Care Versus Telehealth.* J Clin Psychiatry, 2018. **79**(5).
18. Farhang, M., et al., *Impact of mind-body interventions in older adults with mild cognitive impairment: a systematic review.* Int Psychogeriatr, 2019. **31**(5): p. 643-666.
19. Lenze, E.J., et al., *Mindfulness-based stress reduction for older adults with worry symptoms and co-occurring cognitive dysfunction.* Int J Geriatr Psychiatry, 2014. **29**(10): p. 991-1000.
20. Wildes, T.S., et al., *Effect of Electroencephalography-Guided Anesthetic Administration on Postoperative Delirium Among Older Adults Undergoing Major Surgery: The ENGAGES Randomized Clinical Trial.* JAMA, 2019. **321**(5): p. 473-483.

21. Sinclair, S., et al., *Development and validation of a patient-reported measure of compassion in healthcare: the Sinclair Compassion Questionnaire (SCQ).* BMJ open, 2021. **11**(6): p. e045988.
22. Kanter, J.W., et al., *The Behavioral Activation for Depression Scale (BADS): psychometric properties and factor structure.* Journal of Psychopathology and Behavioral Assessment, 2007. **29**(3): p. 191-202.
23. Alexopoulos, G.S., et al., *"Engage" therapy: behavioral activation and improvement of late-life major depression.* The American Journal of Geriatric Psychiatry, 2016. **24**(4): p. 320-326.
24. Fakes, K.L., et al., *Development and Validation of the MiPrep Survey: An Instrument Assessing Patients' Perceived Preparation for Medical Interventions Including Medical Imaging, Radiotherapy, and Surgery.* Value in Health, 2019. **22**(6): p. 704-711.
25. Lamb, S.E., et al., *Development of a common outcome data set for fall injury prevention trials: the Prevention of Falls Network Europe consensus.* J Am Geriatr Soc, 2005. **53**(9): p. 1618-22.
26. Quality, A.f.H.R.a., *About CAHPS*, AHRQ, Editor. 2020: Rockville, MD.
27. Andersen, P.K. and R.G. Gill, *Cox's Regression Model for Coounting Processes: A Large Sample Study.* Ann Statist, 1982. **10**(4): p. 1100-1120.
28. Kroenke, K., et al., *The Patient Health Questionnaire Anxiety and Depression Scale (PHQ-ADS): Initial Validation in Three Clinical Trials.* Psychosom Med, 2016. **78**(6): p. 716-727.
29. Kroenke, K., et al., *Stepped Care to Optimie Pairn Care Effectiveness (SCOPE) Trial: study design and sample characteristics.* Contemp Cln Trials, 2013. **34:**: p. 270-281.
30. Kroenke, K., et al., *Telecare collaborative management of chronic pain in primary care: a randomized clinical trial.* JAMA, 2014. **312**(3): p. 240-248.

RESEARCH PROTOCOL

Behavioral Activation and Medication Optimization For Improving Perioperative Mental Health In Older Adults Undergoing Oncologic Surgery

Principal Investigator:

Benjamin D Kozower MD, MPH
Professor
Department of Surgery
Washington University School of Medicine
660 S. Euclid Ave, Campus Box 8234
Saint Louis, MO, 63110
Tel: 314-362-8089
Fax: 314-362-6288
E-mail: kozower@wustl.edu

Co-Investigators:

Joanna Abraham, PhD
Professor, Department of Anesthesiology and Institute for Informatics
Washington University School of Medicine
660 S. Euclid Ave, Campus Box 8054-50-02
Saint Louis, MO 63110
Tel: 314-362-5129
E-mail: joannaa@wustl.edu

Simon Haroutounian, PhD, MSc
Associate Professor and Division Chief, Department of Anesthesiology
Washington University School of Medicine

Katherine Clifton, MD
Assistant Professor, Department of Medicine - Oncology
Washington University School of Medicine

Kenneth Freedland, PhD
Professor, Department of Psychiatry
Washington University School of Medicine

Michael Avidan, MBBCh
Professor and Chair, Department of Anesthesiology
Washington University School of Medicine

Eric Lenze, MD
Professor and Chair, Department of Psychiatry
Washington University School of Medicine

Katherine Holzer, PhD
Assistant Professor, Department of Anesthesiology
Washington University School of Medicine

Thomas Kannampallil, PhD
Associate Professor, Department of Anesthesiology and Institute for Informatics
Washington University School of Medicine

J. Philip Miller, PhD
Professor
Division of Biostatistics
Washington University School of Medicine

Mary Politi, PhD
Professor, Department of Surgery
Washington University School of Medicine

Ana Baumann, PhD
Assistant Professor, Department of Surgery
Washington University School of Medicine

Bethany Pennington, PharmD
Assistant Professor,
Department of Anesthesiology
Washington University School of Medicine

Theresa Cordner
Senior Research Coordinator
Department of Anesthesiology
Washington University School of Medicine

202212097

Version Date: November 21, 2022
Version 2 Date: March 6, 2023
Version 3 Date: June 12, 2023
Version 4 Date: October 30, 2023

1. SYNOPSIS

**Study Title**	Behavioral activation and medication optimization for perioperative mental health in oncologic surgery
**Abbreviated title**	CPMH Oncologic R34 RCT
**Objective**	Using a Hybrid Type 1 Effectiveness-Implementation RCT design, we will test the effectiveness of a bundled behavioral activation and medication optimization in reducing symptoms of depression and anxiety in older adults undergoing oncologic surgery (compared with usual care), while examining implementation outcomes.
**Study Period**	Planned enrollment duration: 12-15 weeks (an initial visit; follow-up survey at 1 and 3 months)<br><br>Planned study duration: 2 years
**Number of Patients**	100
**Study Treatment**	Behavioral activation and medication optimization
**Study Design**	Hybrid Type 1 Effectiveness-Implementation prospective randomized controlled trial design.
**Inclusion and Exclusion Criteria**	<u>Inclusion criteria</u>:<br>• Adults ≥ 60 years<br>• Scheduled major thoracic, abdominal, or breast surgery for suspected or confirmed malignancy<br>• PHQ-ADS ≥ 10, indicating clinically significant depression or anxiety symptoms<br><u>Exclusion criteria:</u><br>• Barrier to communication (Unable to read, speak, and understand English)<br>• Severe cognitive impairment screened by the SBT (Short Blessed Test) ≥10<br>• Acutely suicidal<br>• Previous participation in this study or another CPMH study of the intervention bundle or its feasibility.
**Measurements**	<u>Baseline</u><br>PHQ-ADS<br>Quality of Life (CDC HRQOL-14)<br>Short Blessed Test (SBT)<br>Presence and intensity of preoperative pain<br><br><u>In-Hospital</u><br>Delirium (extracted from electronic health record retrospectively)<br>Medications of interest<br><br><u>Postoperative</u><br>PHQ-ADS

	Quality of life<br>Rehospitalization<br>Falls<br>Presence and intensity of Persistent post-surgical pain (PPSP)<br>Pain interference, using modified Brief Pain Inventory (BPI)<br>Medications of interest
**Statistical Methodology**	The study will use a modified Intent To Treat (mITT) approach where randomized patients who did not have the surgery or procedure will not be included in the analyses. We will compare the intra-group PHQ-ADS scores at 3 months after surgery as the primary outcome.<br><br>As exploratory outcomes, we will compare each the following outcomes between the groups: (occurrence, or change from baseline to 1-month, and 3-month follow-ups, as appropriate):<br><br>• Quality of life (CDC HRQOL-14)<br>• Length of stay (ICU & hospital)<br>• Delirium<br>• Rehospitalization<br>• Falls<br>• Persistent post-surgical pain presence and pain interference<br>• Medications of interest<br><br>The following measures will be collected from patients allocated to the intervention arm for implementation assessment:<br><br>• Reach<br>• Implementation potential (i.e., Acceptability of intervention; Appropriateness of intervention; Feasibility of intervention)<br>• Patient satisfaction and experiences with overall surgical experience and intervention bundle<br><br>A combined measure including modified versions of the following scales will be collected only from patients allocated to the intervention arm to assess engagement with our intervention bundle approximately 2 months after surgery:<br>• Sinclair Compassion Questionnaire-Short Form (SCQ-SF)<br>• Behavioral Activation for Depression Scale (BADS) Activation subscale<br>• MiPrep survey

2. STUDY PROTOCOL

2.1 Background and Significance

Inadequate management of preoperative mental health disorders often contributes to poor postoperative outcomes, including increased rates of readmission, delirium, falls, and mortality [2-5]. However, very little work has been done to improve perioperative mental health. In particular, there have not been any systematic efforts to identify evidence-based behavioral interventions that were originally developed for depression and anxiety in otherwise medically well psychiatric patients, adapt them for use in surgical populations, test their effectiveness in these patient populations, and study implementation strategies.

Consequently, we currently lack conclusive evidence on effectiveness of interventions specifically focused on the delivery of perioperative mental health care in older surgical patients. To address this understudied research area, the proposed study will focus on comparing a bundled intervention of Behavioral Activation (BA) and Medication Optimization (MO) (compared to usual care) among older adults undergoing oncologic surgery.

The mental health intervention bundle, (Surgical Wellness Program) composed of BA and MO, can mitigate anxiety and depression symptoms during the perioperative period [6]. Towards this end, we propose to test the effectiveness as well as the implementation potential of the intervention bundle in a Hybrid Type 1 Effectiveness-Implementation RCT Design.

2.1.1 Preliminary Data

In an IRB-approved study (IRB # 202101103), we enrolled 8 patients undergoing oncologic surgery to an open-label feasibility phase of the study, to establish recruitment feasibility, and finalize the details pertaining to the study protocol, participant screening, informed consent process, recruitment and follow-up, as well as finalize the workflow for medication optimization and behavioral activation.

Given that these objectives were attained, and reflected in the finalized methodology proposed in this study protocol, we are ready to launch our clinical trial to evaluate the effectiveness and implementation-potential of our intervention bundle using a Hybrid Type 1 Effectiveness-Implementation RCT design.

2.2 Objective

We will test the effectiveness of a bundled intervention comprised of behavioral activation and medication optimization in reducing symptoms of depression and anxiety in older adults undergoing oncologic surgery (compared with usual care), while examining implementation outcomes. The study will be conducted by the members of the newly established Center for Perioperative Mental Health (CPMH), supported by National Institute of Mental Health (NIMH).

2.3 Patient Selection

The study coordinators will screen the Thoracic, Breast, and Hepatobiliary Surgery clinic schedules, as well as the schedule for the Center for Preoperative Assessment and Planning (CPAP) at Barnes-Jewish Hospital for patients undergoing oncologic surgery. Prospective patients will also be identified by Epic workbench reports customized to limit results to those at least 60 years of age with an eligible surgery scheduled at BJH/WUSM. In addition, study collaborators, along with thoracic, breast, and hepatobiliary surgery research support teams, will refer patients from their clinics who meet the inclusion criteria sought from the EHR.

A member of the study team will obtain the patient's phone number, age and sex, as well as type of surgery, the surgeon's name and planned surgery date through Epic. A member of the study team will contact potentially eligible patients to screen for inclusion criteria.

2.3.1 Inclusion Criteria

Pre-screening:

To be considered for participation, patients will be screened using PHQ-4 questionnaire. Scores $\geq$3 will indicate positive pre-screen (i.e. can be considered for participation and formal screening). Scores < 3 will indicate negative screen (i.e. patient not considered for formal screening due to low risk for anxiety or depression).

Screening:

Each participant must meet all of the following criteria:

Inclusion

- Adults age ≥ 60 years
- Scheduled major thoracic, breast, or abdominal surgery for suspected or confirmed malignancy
- PHQ-ADS (Patient Health Questionnaire Anxiety and Depression Scale) ≥10 indicating clinically significant depression or anxiety symptoms.[7]

2.3.2 Exclusion Criteria

Participants will not be enrolled if any of the following criteria exist:

Exclusion

- Barrier to communication (Unable to read, speak, and understand English)
- Severe cognitive impairment screened by the SBT (Short Blessed Test) $\geq$10
- Acutely suicidal
- Considered ineligible per the discretion of the oncologic surgeon or study PI
- Previous participation in this study or another CPMH study of the intervention bundle or its feasibility.

2.4. Study Design

All eligible and consenting patients will be randomized in 1:1 ratio (with no stratifications) to receive either the intervention bundle or usual care.
Consenting participants (in both arms) will undergo a standard battery of assessments at baseline (prior to the intervention in the intervention group) and will be followed until approximately 3 months postoperatively. In conjunction with the effectiveness outcomes data, implementation outcomes related to reach, and implementation feasibility, acceptability, and appropriateness of the intervention bundle will be collected.

2.4.1 Study Procedures

Consented subjects will be asked to participate in the study for approximately 3 months after surgery. All participants must sign a consent document (e-consent or paper), indicating their consent,

approved by the Washington University School of Medicine Institutional Review Board, before they can participate in the study.

All participants will be asked to complete the following questionnaires at baseline (in person, remotely via a web-based survey, or via telephone):

- CDC HRQOL-14 Quality of Life survey
- PHQ-ADS (Patient Health Questionnaire Anxiety and Depression Scale)
- SBT (Short Blessed Test)
- Presence and intensity of preoperative pain
- Demographics survey
- Patient-reported falls

All participants will be followed for up to approximately 3 months after their scheduled procedure. They will complete follow-up surveys at approximately 1 month and 3 months after surgery by either web-based survey, mail, or via a telephone interview.

At the end of the study, all participants in the intervention arm will be invited to take part in a semi-structured telephone interview conducted by an experienced, qualitative researcher with whom they had no previous contact. The interviews will explore the participants' perceptions, attitudes, and experiences of the intervention bundle.

2.4.2 Minimization of Bias

Enrollment will not be based on sex or ethnic background. Standard of care will not be affected for any of the participants due to study participation. Completion of the outcomes will be entirely patient-reported, to minimize assessor's bias in interpreting patient responses.

2.4.3 Pre-Study Period

Patients who are ≥ 60 years of age undergoing oncologic surgery will be approached for consent to this study. Eligible patients will be invited to participate in the study, which will be conducted remotely and in-person. Potential candidates for the study will be provided a written and oral description of the study procedures, benefits, and potential risks as well as the opportunity to ask questions regarding the study. Participants are informed that their participation is voluntary and that they may refuse to participate or withdraw from the study at any time without penalty. Patients will be required to give informed consent prior to participation. A study team member who is experienced with cognitive testing will recruit the subjects to the study, and perform the SBT assessment.

Each subject who qualifies for entry into the study on the basis of inclusion/exclusion criteria and completion of informed consent will be assigned the next available patient number. This indicates enrollment in the study. Patients who are eligible but decline participation will be invited to complete an optional survey indicating reasons for decline.

2.4.4. Randomization

The randomization table will be generated by the study biostatistician, using a variable block sequence. The assignment of the treatment condition for each individual participant will be done by the REDCap randomization module after they have been determined to be eligible for the trial.

2.4.5. Blinding

Study staff responsible for administering ratings throughout the study are blinded. This blinding is kept throughout the study until data collection is complete. At the end of the follow-up, the study coordinator will complete a blinding question to try to guess the arm to which each participant was allocated. This will allow to determine whether the blinding procedures in the study were effective [8].

2.4.6 Study Period

Baseline Characterization of Participants: At enrollment, a battery of assessments will characterize participants. *Anxiety and depression* assessment will use the PHQ-ADS, as described above. The use of this tool for this population is supported by a recent meta-analysis suggesting that the PHQ9 (on which this tool is based) has good sensitivity and specificity in the diagnosis of depression in older adults [9]. *Quality of Life* (QoL) will be assessed using the CDC HRQOL-14 survey) [10]. The questions in this patient-reported survey correspond to the QoL domains of Healthy days (physical and mental), Activity Limitations, and Healthy Days Symptoms. *Cognitive function* assessment will be performed using the Short Blessed Test (SBT). SBT is a reliable test, with high inter-rater agreement, which is beneficial for characterizing cognitive functioning in the perioperative setting [11,12]. Recognizing the uncertainty associated with face-to-face data collection due to the COVID-19 pandemic, we selected tools such as the SBT because it can be delivered remotely [13].

All patients in both arms will receive resources (see Handouts for CPMH RCT SOC Group and Handouts for CPMH RCT Intervention Group) either by mail or email if they prefer. The resources include options for exploring brain health, supporting sleep and mental health, and introducing community resources for older adults.

Intervention Bundle: This will be initiated prior to or soon after the surgery and will be continued until approximately 3 months postoperatively.

The basic premise of *behavioral activation* is to help people with mental health problems to engage in activities that are reinforcing or meaningful and guided by their personal values [14]. Patients will be asked to check with their physicians if there is any question about the safety of any physical activities that are included in the behavioral activation plan. Behavioral activation as the core intervention allows for uniformity across participants from each cohort, yet enough flexibility for the actual components of behavioral activation to be individually adapted based on patient preferences. In a recent meta-analysis, behavioral activation programs improved depression and anxiety [15], as well as disability in older adults [16]. In addition, behavioral activation delivered by telehealth to older adults was cost-effective and lowered health care utilization costs over 1 year [17]. In addition to the core intervention of behavioral activation, study participants will tailor the intervention by choosing activities, per their preference, with demonstrated benefit in improving depression and anxiety symptoms in older adults [18,19]. Behavioral activation will generally begin pre-operatively, with sessions approximately every two weeks. After discharge, the behavioral intervention will continue, for 10-12 sessions, or out to approximately 3 months postoperatively. Patients in the intervention arm will receive information (Handouts for CPMH RCT Intervention Group) by mail or email if they prefer. The handouts include tools to support behavioral activation and optional activities.

Medications will be *reviewed and optimized*, as appropriate. In several clinical trials including thousands of older adults, we have developed and refined a process for this review and medication optimization, including adjusting suboptimal dosing for anti-depressants and discontinuing medications that are harmful for brain health (such as anticholinergics [20]). Medication optimization consists of a simple set of principles: reconcile patient's medications, identify the patient's likely need for, and interest in, a medication adjustment, make the adjustment, and assess the response to

that adjustment. Benzodiazepines, and strong centrally acting anticholinergics and antihistamines, are the focus of *deprescribing*, as they increase the risk for falls and delirium. We will follow the principles of shared decision-making with patients for medication changes. While the participant is an inpatient, the study pharmacy team will coordinate with the inpatient team to ensure that medication changes that were introduced preoperatively are maintained in-house and that no new inappropriate medications are initiated. After discharge, and up to approximately 3 months postoperatively, the interventionist (also referred to as a perioperative wellness partner) and pharmacy team will ensure that targeted medication changes are reconciled during transitions of care.

Methods

2.4.6 Observations and Measurements

2.4.6.3 Primary Outcome Measures

Effectiveness outcomes will include the primary outcome measure of PHQ-ADS, a validated composite scale of depression and anxiety symptoms [7], measured at 1 and 3 months after surgery. Three months post-operatively is the primary endpoint.

Process measure for target engagement

As a process measure of target engagement at approximately 2 months after surgery, we will use a combined measure including modified versions of the Sinclair Compassion Questionnaire-Short Form (SCQ-SF) [21], Behavioral Activation for Depression Scale (BADS) Activation subscale [22,23], the MiPrep survey [24], four questions specific to our Wellness Program, and one question to assess participants' understanding of how the study's target medications can affect their mental health, if applicable. The target engagement measure will be collected by survey, which participants in the intervention group can complete either online via REDCap link or by phone if they prefer, with an unblinded member of the study team.

2.4.6.4 Exploratory Outcome Measures

The exploratory outcomes will include QoL measured via the CDC HRQOL-14 survey, and other perioperative outcomes of interest, nominated by our stakeholders as key outcomes of interest, including:

(1) In-hospital: (i) Delirium incidence,[20], (ii) Length of Stay (hospital and ICU),(iii) information on the use of common medications for chronic pain, such as opioids, gabapentin and pregabalin.

(2) Post-discharge: (i) Falls, (ii) All-cause rehospitalization out to 90 days postoperative, (iii) persistent post-surgical pain (PPSP), (iv) information on the use of common medications for chronic pain, such as opioids, gabapentin and pregabalin.

(3) Process outcomes: For patients on the intervention arm, CAHPS modified survey will be administered after the intervention is complete.

(4) Implementation-potential surveys: Acceptability of Intervention Measure, Intervention Appropriateness Measure, Feasibility of Intervention Measure.

Table 2. Details on Study Outcomes

Effectiveness Outcomes	Data Source	Description	Data Collection Mechanism	Time points (Baseline, 1, 2, 3 month-

					**post-op or EOS)**
**Primary *(Clinical)***	Anxiety and Depression	Patient Health Questionnaire Anxiety and Depression Scale (PHQ-ADS)	A validated composite of symptoms of depression and anxiety	Patient Self-Report	Baseline; 1 mo and 3 mo
Exploratory (Clinical)	Quality of Life	The Health-Related Quality of Life CDC HRQOL-14 survey [10]	A patient-reported global health measure. The questions in this survey correspond to the QOL domains of Healthy days (physical and mental), Activity Limitations, and Healthy Days Symptoms	Patient Self-Report	Baseline; 1 mo and 3 mo
	In-hospital delirium incidence	Retrospective EHR chart review	.	EHR report, and Research Team Member review EHR for delirium-related information from the time of post-op inpatient stay.	In-hospital: at ICU and hospital floor
	Post-discharge falls	Patient Reported Falls	A Patent Reported Falls survey will be collected at 1 month and 3 months either by phone or REDCap link, at follow-up clinic appointments.	Patient Self-Report	Baseline; 1mo and 3mo
	Pain	Pain presence and severity at the site of surgery, and Brief Pain Inventory (BPI) pain interference scale	BPI is a well validated measure of pain severity and pain interference items.	Patient self-report	Baseline, 1 mo, 3 mo

	Length of stay (both hospital and ICU)	Research Data Core	Standardized reports on RDC warehouse will generate statistics of enrolled patients regarding the length of stay, rehospitalization, and related clinical metrics.	EHR report	In-hospital: at ICU and hospital discharge
	All-cause rehospitalization	EHR and patient		EHR, patient	Post-op: 1 mo and 3 mo
**Implementation and Process Outcomes**		**Data Source**	**Description**	**Data Collection Mechanism**	**Time points**
Reach		Enrollment Log	Reach of the study: patients who agreed to participate in the study out of total eligible to participate<br>Reach of the intervention bundle: patients who completed the interventions out of patients who agreed to participate	Electronic Health Record	End of Study
Implementation-Potential (I)		Acceptability of Intervention Measure, Intervention Appropriatenes s Measure, Feasibility of Intervention Measure	Acceptability, Feasibility and Appropriateness 4-item Likert scale survey	Patient Self-Report	Post-EOS
Patient satisfaction and experience		Modified-Consumer Assessment of Healthcare Providers and Systems (CAHPS)[25]	The modified CAHPS asks patients about their experience with the intervention bundle before surgery, during surgery, and after surgery.	Patient Self-Report	Post-EOS
**Process measure for target engagement**		**Data Source**	**Description**	**Data Collection Mechanism**	**Time points**

Target engagement measure	Combined measure of modified versions of the following scales: Sinclair Compassion Questionnaire-Short Form (SCQ-SF)[21], Behavioral Activation for Depression Scale (BADS) Activation subscale [22], the MiPrep survey[24]	The SCQ-SF is a 5-item patient-reported measure modified to assess compassion of the Wellness Provider; the BADS Activation Subscale (7 items) measures target engagement of BA in studies of geriatric depression [23]; and the MiPrep survey (3 items) assesses patients' preparation for medical interventions.	Patient Self-Report	Approximately 2 mo post-op

In addition to these outcomes, we will assess fidelity to our intervention bundle to ascertain the extent to which an intervention bundle is carried out, as intended and consistently across different settings, and patients. If the patient agrees to audio/video recording, the intervention sessions will be audio recorded (or audio/video if the patient prefers to meet by Zoom teleconference). To track this, we will use the recordings of these discussions between the patient and the perioperative wellness partner during the behavioral intervention and medication optimization sessions. We will capture data on patient interactions with the wellness partner, their delivery of the intervention bundle, any observed barriers and facilitators to the intervention adherence, acceptance and fit within the perioperative workflow. 10% of these sessions of patients and their wellness partners will be selected based on a convenience sample for evaluating fidelity of the bundle.

Lastly, we will also obtain stakeholder perspectives on the intervention bundle after the intervention period has been completed using semi-structured interviews. We will conduct interviews with patients who participated in the intervention, and the topics of discussions will be guided by an implementation science framework referred to as CFIR (Consolidated Framework of Implementation Research). The interviews will explore the participants' perceptions, attitudes, and experiences with the intervention bundle, intervention bundle acceptability and detailed accounts of participants' experiences after the intervention has been stopped with regards to intervention sustainability and maintenance. Interviews will be conducted via Zoom or telephone and will be digitally recorded and transcribed verbatim.

Study Calendar

	Time 0	Baseline/ Time 1	1 mo follow up	2 mo follow up	3 mo follow up	Post-EOS
Patient Consent	X					
Patient Screening	X					
Patient Baseline surveys		X				
Patient Follow-up 1 & 3 mo Surveys			X		X	
For Patients on the Intervention Arm:				X		

Process Target Engagement Measure						
For Patients on the Intervention Arm: Intervention-Patient Interviews and Implementation, Patient Satisfaction and Experience surveys						X
For Patients on the Intervention Arm: Behavioral Activation and Medication Review Intervention Sessions			Approximately 8-12 sessions as needed (per Pt preference) from Baseline, continuing for up to approximately 3 months post-op during study data collection, until EOS.			

2.4.6.5 Statistical Methods

Analyses were conducted in a modified intention-to-treat population, including all randomized participants except those whose surgery was canceled after randomization. The prespecified statistical analysis plan is available in the Statistical Analysis Protocol (Supplement 1 below). Internal consistency (Cronbach α) was calculated for the PHQ-9 and GAD-7 at baseline and 3 months within each cohort.

The primary outcome was analyzed separately for each cohort using repeated-measures mixed models with fixed effects for treatment group, time (baseline, 1 month, and 3 months), and their interaction. Least-squares mean changes from baseline to 3 months were compared between groups, with sensitivity analyses adjusting for baseline values. A prespecified, individual-patient, 2-stage, fixed-effect meta-analysis summarized treatment effects across the three cohorts. This is logically equivalent to a single fixed-effect model in which the study group is a fixed effect and the inference is whether there is a mean intervention effect in these three specific groups taken as a whole.

Other outcomes measured longitudinally were analyzed using analogous mixed models. Length of stay was analyzed using analysis of covariance; delirium, rehospitalization, and persistent postsurgical pain using modified Poisson regression with a log link; and falls using repeated-measures mixed models adjusted for baseline. Prespecified covariates were included where appropriate, and potential effect modification by sex was examined. Analyses were performed using SAS, version 9.4M8 (SAS Institute Inc, Cary, NC, USA).

All models were adjusted for sex assigned at birth, surgery type based on complexity, invasiveness, and length of recovery, and ASA (American Society of Anesthesiologists) physical status score, a validated measure of perioperative risk based on medical comorbidities that effectively discriminates between low- and high-risk patients. All pain models additionally included chronic pain and opioid use as covariates. A meta-analytical approach was used to allow the effects of covariates to differ across surgical cohorts. This approach also accommodated potential differences in residual error variance across cohorts, resulting in simpler models. It was particularly appropriate because the distributions of surgery type were not equivalent across the surgical cohorts.

2.4.6.6 Sample Size and Power of Primary Analysis:

In order to estimate the power of the primary outcome analysis we examined the manuscript which provided the original validity and reliability studies for the PHQ-ADS [26] and one of the trials which provided data for that study (SCOPE) [27,28]. At 3 months they observed a change from baseline divided by the standard deviation of the change of 0.43 which corresponds to a change of 3 points on

the PHQ-ADS. The PHQ-ADS authors estimate the minimal important clinical difference (MICD) from the standard error of measure (SEM) as 3-4 points. If we postulate a difference in the change scores of 2 points between the two groups, then with a power of 0.80 we would need 36 in each group. Since the SCOPE trial was a pain relief study without a requirement of anxiety or depression, we consider these estimates rough estimates. This trial will have 100 mITT patients (50 per arm) in order to account for any incomplete data among recruited participants. Recruitment will continue until 100 randomized patients have had their procedure or surgery.

2.5 Management of Intercurrent Events

2.5.1 Adverse Experiences

Based on the nature of this study, which involves surveys, a cognitive test, and a behavioral intervention, adverse events are exceedingly unlikely.

2.5.2 Premature Withdrawal

Subjects will be withdrawn if the investigator decides that discontinuation is in the best interest of the subject, or the subject requests withdrawal from the study. Additionally at PI discretion, they may be withdrawn if their surgery is cancelled or postponed (for clinical or non-clinical reasons) for more than approximately 3 months after baseline.

Participants who choose to end study participation early, may be asked to complete early termination assessments. Additionally, those in the intervention arm may choose to withdraw from that at any time, but will be allowed to continue with data collection follow-ups if they still want to participate.

2.5.3 Potential Risks

This study involves minimal risk to subjects. Unlikely but potential risks include increased risk of depression or anxiety, errors in medication recommendations or medication withdrawal symptoms, and breach of confidentiality. The risk of medication withdrawal (i.e., from benzodiazepines) is mitigated by slowly tapering rather than stopping these medications. Patients who are identified as being acutely suicidal will be excluded from the study. Nevertheless, since the rate of completed suicide in the USA remains high (i.e., about twice the rate of homicide) and most Americans who commit suicide suffer from depression, all participants eligible to participate in this study are statistically at a relatively higher risk for suicide than the general population. However, the participants' absolute risk for completing suicide during this brief study remains very low (i.e., about 1 in 3,000 to 10,000) and participation in the study does not create or increase the risk of completed suicide; rather, most experts believe that one of the most efficient ways to decrease suicidal risk in older depressed individuals is to treat their depression. Furthermore, all participants will be formally assessed frequently throughout the study. If the study personnel identify that a participant has become acutely suicidal, this participant will be referred to a mental health professional for further evaluation and treatment (see section: Procedures to Minimize Risks). This may lead to a clinical intervention that is lifesaving and may not have occurred had the participant not been participating in the study.

2.5.4 Procedures to Minimize Potential Risks

Medication Error Risk: Dr. Eric Lenze will supervise careful training and oversite of the intervention pharmacists making medication optimization recommendations to reduce the risk of medication error or medication withdrawal symptoms. Algorithms for tapering medications that should not be

abruptly discontinued will be employed. We have carried out this deprescribing protocol in several large RCTs and have not seen any problems occur.

Suicide risk management: As discussed above, at each assessment point, the research staff will assess for passive death wish, and suicidal ideation, intent or plan when they administer the PHQ-ADS. If a participant endorses suicidal ideation, intent, or plan, the rater will be trained to follow an operationalized protocol that has been developed to manage high-risk participants in other studies of depressed participants potentially at risk for suicide. This protocol has already been used successfully by Dr. Lenze's team to manage acutely suicidal patients. Briefly, the protocol entails a specific determination of the suicidal risk and prescribes a set of actions. For instance, when a participant is determined to be at high and immediate risks, the rater is instructed to stay with the participant until he or she has contacted a study psychiatrist (Dr. Lenze) to discuss the situation and to devise a plan. For this reason, raters will have cell phones and Dr. Lenze will be reachable via email and cell phone. In case of extreme emergency, raters are instructed to call their hospital security team or 911 for immediate help and to initiate commitment proceedings.

Patients will be asked to check with their clinician if there is any question about the safety of any physical activities that are included in the behavioral activation plan.

The risk of breach of confidentiality will be minimized by ensuring that protected health information will only be shared with members of the research team. Only data necessary to complete the study will be collected. Electronic data will be kept in a password-protected electronic database only assessable via password protected departmental computers. Patients may choose to not participate in the study or withdraw at any time with no penalty in terms of the care they receive.

2.5.5 Data and Safety Monitoring Plan

The specific monitoring plan for this investigation is commensurate with the risks and the size and complexity of the studies planned. Given the nature of the protocol, the risks are likely limited to breach of confidentiality. The Principal Investigator will be responsible for monitoring the data and assuring protocol compliance. The PI will meet with the clinical research coordinator and Data Manager bi-weekly to review enrollment, eligibility, participant follow-up and outcome assessment. The PI and study coordinator will monitor for breaches of confidentiality on an ongoing basis. Study-related adverse events will be reported per IRB guidelines. All serious adverse events (SAE) will be recorded and adjudicated by a CPMH Principal Investigator and reported per IRB guidelines. Any endorsement of suicidal ideation will be managed acutely as detailed in the section 2.5.4. If study staff becomes aware of a reportable adverse event, it will be reported to necessary stakeholders according to Washington University Human Protection Office guidelines. This type 1 hybrid randomized study does not require a Data and Safety Monitoring Board.

3. HUMAN SUBJECTS RESEARCH

3.1 Protection of Human Subjects

The study will be conducted under appropriate Washington University Institutional Review Board protocols and consent forms approvals. The study will be conducted under the supervision of the PI, who has extensive of experience in the conduct of human research studies.

3.2 Sources of Materials

Preoperative assessments along with preoperative and postoperative questionnaires regarding quality of life, depression, and anxiety will be obtained from all study participants. The results of follow-up questionnaires will be obtained via e-mail, mail, or telephone survey. Data will be collected at baseline, one, two, and 3 months following surgical procedure.

3.2.1 List of Protected Health Information Collected for Study

The HIPAA protected identifiers that will be collected for this study include:

- Name
- Dates
  - Date of birth
  - Date of services (surgical procedure)
- Contact information
  - Mailing address
  - Phone number
  - Electronic mail address
- Medical record number

3.2.2 Data Management

All paper data will be stored under lock and key (office, file cabinet) and all electronic data will be stored in REDCap, only accessible to the research team members. Surveys that are completed electronically will be administered via the REDCap server. All REDCap email correspondence will be sent using secure survey links. All survey information will be coded with the patient's unique study ID number and no personal health information that may directly identify patients will be included.

3.3 Recruitment and Informed Consent

Participants will be recruited either from the oncologic surgery clinics at Washington University or the Center for Preoperative Assessment and Planning (CPAP) at Barnes-Jewish Hospital/Washington University. Participants with pre-screening scores of PHQ-4 ≥3 will be recruited, and eligibility confirmed if PHQ-ADS ≥10. Retention strategies will include flexibility in scheduling follow-up visits and coordination of research visits with any in-person clinical visits. Subjects will be given verbal and then written descriptions of the study aims, procedures, risks, and benefits, and will be required to give informed consent. A member of the research team will be available to present study descriptions, informed consent, and answer any questions. Subjects are informed verbally and in writing that participation is voluntary and they may refuse to participate and may withdraw from the study at any time without penalty. Remuneration for each data collection study visit will be provided to encourage retention as well.

3.4 Potential Benefits of the Proposed Research to the Subjects and Others

Although the study is designed to improve symptoms of depression and anxiety and other outcomes of care, there is no guarantee that the study participants will receive any clinical benefits by participating in the study. This may lead to a clinical intervention that is lifesaving and may not have occurred had the participant not been participating in the study. Given the undertreatment of depression and anxiety in older adults, the minimal risks are acceptable in comparison to the potential benefits in improving the mental health of older adults undergoing surgery.

3.5 Remuneration

Patients will receive remuneration by a reloadable debit card of $25 for completion of each data collection study visit. Standard Care (control arm) participants will be compensated for their time at Baseline, 1, and 3 month post-op, up to $75 for completion of all data collection time points. Wellness Program (intervention arm) participants will be compensated for their time at Baseline, 1, and 3 month post-op, plus a post-EOS semi-structured interview, so up to $100 for completion of all data collection time points.

3.6 Inclusion of Women

Eligible individuals will be offered the opportunity to participate in the study regardless of gender or minority status. Thus, the sample composition will reflect the composition of community-living older adults in the St. Louis area.

3.7 Inclusion of Minorities

All of our studies actively encourage the participation of minorities in the research. Our minority recruiting typically matches the demographic composition of the Washington University community from which subjects will be recruited (78% white, 21% Black, <1 % Hispanic).

3.8 Inclusion of Children

We will not be including children in this study.

4. REFERENCES

1. Olive J.K., et al., *Impact of Psychiatric Comorbidities on Surgical Outcomes for Non-Small Cell Lung Cancer*. The Annals of Thoracic Surgery, 2022. **113**(3):p. 1008-14.
2. Huyan T., et al., *Perioperative dexmedetomidine reduces delirium in elderly patients after lung cancer surgery*. Psychiatria Danubina, 2019. **31**(1):p. 95-101.
3. Wada S., et al., *Preoperative anxiety as a predictor of delirium in cancer patients: a prospective observational cohort study*. World journal of surgery, 2019. **43**(1):p. 134-42.
4. Tuveri M., et al., *The prognostic role of fatigue, depression and anxiety on postoperative outcomes after pancreatectomy for pancreatic cancer. A prospective observational study (FAT-PRO study)*. Pancreatology, 2022. Epub ahead of print.
5. Housman B., et al., *Narrative review of anxiety and depression in patients with esophageal cancer: underappreciated and undertreated*. Journal of Thoracic Disease, 2021. **13**(5):p. 3160.
6. Abraham, J., et al., *A Qualitative Study of Perioperative Depression and Anxiety in Older Adults.* Am J Geriatr Psychiatry, 2020.
7. Kroenke, K., et al., *Patient Health Questionnaire Anxiety and Depression Scale: Initial Validation in Three Clinical Trials.* Psychosom Med, 2016. **78**(6): p. 716-27.
8. Freed, B., et al., *Assessing blinding in trials of psychiatric disorders: a meta-analysis based on blinding index.* Psychiatry Res, 2014. **219**(2): p. 241-7.
9. Levis, B., et al., *Accuracy of Patient Health Questionnaire-9 (PHQ-9) for screening to detect major depression: individual participant data meta-analysis.* BMJ, 2019. **365**: p. l1476.
10. Mielenz, T.J., L.F. Callahan, and M.C. Edwards, *Item response theory analysis of Centers for Disease Control and Prevention Health-Related Quality of Life (CDC HRQOL) items in adults with arthritis.* Health Qual Life Outcomes, 2016. **14**: p. 43.
11. Gregory, S.H., et al., *Abnormal preoperative cognitive screening in aged surgical patients: a retrospective cohort analysis.* Br J Anaesth, 2021. **126**(1): p. 230-237.
12. Oughli, H.A., et al., *Cognitive Improvement in Older Adults in the Year After Hip Fracture: Implications for Brain Resilience in Advanced Aging.* Am J Geriatr Psychiatry, 2018. **26**(11): p. 1119-1127.
13. Arias, F., et al., *Rapid In-Person Cognitive Screening in the Preoperative Setting: Test Considerations and Recommendations from the Society for Perioperative Assessment and Quality Improvement (SPAQI).* Perioper Care Oper Room Manag, 2020. **19**.
14. Puspitasari, A.J., et al., *A randomized controlled trial of an online, modular, active learning training program for behavioral activation for depression.* J Consult Clin Psychol, 2017. **85**(8): p. 814-825.
15. Stein, A.T., et al., *Looking beyond depression: a meta-analysis of the effect of behavioral activation on depression, anxiety, and activation.* Psychol Med, 2020: p. 1-14.
16. Choi, N.G., et al., *Improving Social Connectedness for Homebound Older Adults: Randomized Controlled Trial of Tele-Delivered Behavioral Activation Versus Tele-Delivered Friendly Visits.* Am J Geriatr Psychiatry, 2020.
17. Egede, L.E., et al., *Cost-Effectiveness of Behavioral Activation for Depression in Older Adult Veterans: In-Person Care Versus Telehealth.* J Clin Psychiatry, 2018. **79**(5).
18. Farhang, M., et al., *Impact of mind-body interventions in older adults with mild cognitive impairment: a systematic review.* Int Psychogeriatr, 2019. **31**(5): p. 643-666.
19. Lenze, E.J., et al., *Mindfulness-based stress reduction for older adults with worry symptoms and co-occurring cognitive dysfunction.* Int J Geriatr Psychiatry, 2014. **29**(10): p. 991-1000.
20. Wildes, T.S., et al., *Effect of Electroencephalography-Guided Anesthetic Administration on Postoperative Delirium Among Older Adults Undergoing Major Surgery: The ENGAGES Randomized Clinical Trial.* JAMA, 2019. **321**(5): p. 473-483.

21. Sinclair, S., et al., *Development and validation of a patient-reported measure of compassion in healthcare: the Sinclair Compassion Questionnaire (SCQ).* BMJ open, 2021. **11**(6): p. e045988.
22. Kanter, J.W., et al., *The Behavioral Activation for Depression Scale (BADS): psychometric properties and factor structure.* Journal of Psychopathology and Behavioral Assessment, 2007. **29**(3): p. 191-202.
23. Alexopoulos, G.S., et al., *"Engage" therapy: behavioral activation and improvement of late-life major depression.* The American Journal of Geriatric Psychiatry, 2016. **24**(4): p. 320-326.
24. Fakes, K.L., et al., *Development and Validation of the MiPrep Survey: An Instrument Assessing Patients' Perceived Preparation for Medical Interventions Including Medical Imaging, Radiotherapy, and Surgery.* Value in Health, 2019. **22**(6): p. 704-711.
25. Lamb, S.E., et al., *Development of a common outcome data set for fall injury prevention trials: the Prevention of Falls Network Europe consensus.* J Am Geriatr Soc, 2005. **53**(9): p. 1618-22.
26. Quality, A.f.H.R.a., *About CAHPS*, AHRQ, Editor. 2020: Rockville, MD.
27. Andersen, P.K. and R.G. Gill, *Cox's Regression Model for Coounting Processes: A Large Sample Study.* Ann Statist, 1982. **10**(4): p. 1100-1120.
28. Kroenke, K., et al., *The Patient Health Questionnaire Anxiety and Depression Scale (PHQ-ADS): Initial Validation in Three Clinical Trials.* Psychosom Med, 2016. **78**(6): p. 716-727.
29. Kroenke, K., et al., *Stepped Care to Optimie Pairn Care Effectiveness (SCOPE) Trial: study design and sample characteristics.* Contemp Cln Trials, 2013. **34:**: p. 270-281.
30. Kroenke, K., et al., *Telecare collaborative management of chronic pain in primary care: a randomized clinical trial.* JAMA, 2014. **312**(3): p. 240-248.

RESEARCH PROTOCOL

Behavioral Activation and Medication Optimization For Improving Perioperative Mental Health In Older Adults Undergoing Orthopedic Surgery

Principal Investigator:

Ryan P. Calfee, MD, MSc
Professor, Department of Orthopedic Surgery
Washington University School of Medicine
660 S. Euclid Ave, Campus Box 8233
Saint Louis, MO, 63110
Tel: 314-362-9369
Fax: 314-747-2599
E-mail: calfeer@wustl.edu

Co-Investigators:

Kimberly Bartosiak, MD
Assistant Professor, Department of Orthopedic Surgery
Washington University School of Medicine

Michael Avidan, MBBCh
Professor and Chair, Department of Anesthesiology
Washington University School of Medicine

Eric Lenze, MD
Professor and Chair, Department of Psychiatry
Washington University School of Medicine

Joanna Abraham, PhD
Associate Professor, Department of Anesthesiology and Institute for Informatics
Washington University School of Medicine

Katherine Holzer, PhD
Assistant Professor, Department of Anesthesiology
Washington University School of Medicine

Simon Haroutounian, PhD
Associate Professor
Division of Clinical and Translational Research
Department of Anesthesiology
Washington University School of Medicine

Kenneth Freedland, PhD
Department of Psychiatry

Washington University School of Medicine

Thomas Kannampallil, PhD
Associate Professor, Department of Anesthesiology and Institute for Informatics
Washington University School of Medicine

J. Philip Miller, PhD
Professor
Division of Biostatistics
Washington University School of Medicine

Mary Politi, PhD
Professor, Department of Surgery
Washington University School of Medicine

Ana Baumann, PhD
Assistant Professor, Department of Surgery
Washington University School of Medicine

Bethany Pennington, PharmD
Assistant Professor,
Department of Anesthesiology
Washington University School of Medicine

Theresa Cordner
Senior Research Coordinator
Department of Anesthesiology
Washington University School of Medicine

202301070

Version Date: January 12, 2023
Version 2 Date: August 03, 2023
Version 3 Date: October 30, 2023

1. SYNOPSIS

**Study Title**	Behavioral activation and medication optimization for perioperative mental health in orthopedic surgery
**Abbreviated title**	CPMH Orthopedic R34 RCT
**Objective**	Using a Hybrid Type 1 Effectiveness-Implementation RCT design, we will test the effectiveness of a bundled behavioral activation and medication optimization in reducing symptoms of depression and anxiety in older adults undergoing orthopedic surgery (compared with usual care), while examining implementation outcomes.
**Study Period**	Planned enrollment duration: 12-15 weeks (an initial visit; follow-up survey at 1 and 3 months)<br><br>Planned study duration: 2 years
**Number of Patients**	100
**Study Treatment**	Behavioral activation and medication optimization
**Study Design**	Hybrid Type 1 Effectiveness-Implementation prospective randomized controlled trial design.
**Inclusion and Exclusion Criteria**	<u>Inclusion criteria</u>:<br>• Adults ≥ 60 years<br>• Scheduled primary hip or primary knee arthroplasty procedure<br>• PHQ-ADS ≥ 10, indicating clinically significant depression or anxiety symptoms<br><u>Exclusion criteria:</u><br>• Barrier to communication (Unable to read, speak, and understand English)<br>• Severe cognitive impairment screened by the SBT (Short Blessed Test) >10<br>• Acutely suicidal<br>• Previous participation in this study or another CPMH study of the intervention bundle or its feasibility.
**Measurements**	<u>Baseline</u><br>PHQ-ADS<br>Quality of Life (CDC HRQOL-14)<br>Short Blessed Test (SBT)<br>Presence and intensity of preoperative pain<br><br><u>In-Hospital</u><br>Delirium (extracted from electronic health record retrospectively)<br>Medications of interest<br><br><u>Postoperative</u><br>PHQ-ADS<br>Quality of life<br>Rehospitalization

	Falls<br>Presence and intensity of Persistent post-surgical pain (PPSP)<br>Pain interference, using modified Brief Pain Inventory (BPI)<br>Medications of interest
**Statistical Methodology**	The study will use a modified Intent To Treat (mITT) approach where randomized patients who did not have the surgery or procedure will not be included in the analyses. We will compare the intra-group PHQ-ADS scores at 3 months after surgery as the primary outcome.<br><br>As exploratory outcomes, we will compare each the following outcomes between the groups: (occurrence, or change from baseline to 1-month, and 3-month follow-ups, as appropriate):<br>• Quality of life (CDC HRQOL-14)<br>• Length of stay (ICU & hospital)<br>• Delirium<br>• Rehospitalization<br>• Falls<br>• Persistent post-surgical pain presence and pain interference<br>• Medications of interest<br><br>The following measures will be collected from patients allocated to the intervention arm for implementation assessment:<br>• Reach<br>• Implementation potential (i.e., Acceptability of intervention; Appropriateness of intervention; Feasibility of intervention)<br>• Patient satisfaction and experiences with overall surgical experience and intervention bundle<br><br>A combined measure including modified versions of the following scales will be collected only from patients allocated to the intervention arm to assess engagement with our intervention bundle approximately 2 months after surgery:<br>• Sinclair Compassion Questionnaire-Short Form (SCQ-SF)<br>• Behavioral Activation for Depression Scale (BADS) Activation subscale<br>• MiPrep survey

2. STUDY PROTOCOL

2.1 Background and Significance

One in 10 American adults has symptomatic osteoarthritis affecting the lower extremity [1]. As a result, the number of total hip and knee arthroplasties has propelled osteoarthritis-related surgeries to the 4th leading cause for hospitalization in the United States [1]. As our population ages, the number of total hip and knee arthroplasties is projected to exceed 4 million procedures annually by 2030 [2]. With increasing numbers of primary arthroplasties, the number of revision surgeries will also rise toward 350,000 annually. Revision surgery is performed to address unsatisfactory outcomes, which occur frequently. Revision is more complex and carries a higher risk than primary arthroplasty. Up to 30% of patients report ongoing disability following hip arthroplasty [3], and 20% of patients have pain that limits function, despite technically sound total knee arthroplasty [4].

Mechanical prosthetic failure accounts for only 5% of poor outcomes following knee arthroplasty [5]. This pales in comparison to the 35% of revision knee arthroplasties performed for unexplained pain [6]. Such pain is invariably related to mental health and biopsychosocial factors, as 20-30% of patients demonstrate generalized anxiety or major depression before total hip and knee arthroplasty [7]. Across multiple studies, the strongest and most consistent variables associated with unsatisfactory pain and functional outcomes after hip and knee arthroplasty are comorbid mental health conditions, including anxiety, depression, and pain castrophization [8].

Despite the clear impact of mental health on hip and knee arthroplasty outcomes, research has yet to produce a consensus on an optimal mental health intervention. A 2018 systematic review of psychological interventions in total hip and knee arthroplasty identified only 7 randomized intervention trials, and concluded that this body of literature was, "still in its infancy."[9]. Many previous trials examining preoperative mental health interventions have not incorporated patient-specific tailored interventions, did not combine behavioral and pharmacological strategies, and often lacked multidisciplinary teams [9]. However, very little work has been done to improve perioperative mental health.

In particular, there have not been any systematic efforts to identify evidence-based behavioral interventions that were originally developed for depression and anxiety in otherwise medically well psychiatric patients, adapt them for use in surgical populations, test their effectiveness in these patient populations, and study implementation strategies.

Consequently, we currently lack conclusive evidence on effectiveness of interventions specifically focused on the delivery of perioperative mental health care in older surgical patients. To address this understudied research area, the proposed study will focus on comparing a bundled intervention of Behavioral Activation (BA) and Medication Optimization (MO) (compared to usual care) among older adults undergoing orthopedic surgery.

The mental health intervention bundle, (Surgical Wellness Program) composed of BA and MO, can mitigate anxiety and depression symptoms during the perioperative period [6]. Towards this end, we propose to test the effectiveness as well as the implementation potential of the intervention bundle in a Hybrid Type 1 Effectiveness-Implementation RCT Design.

2.1.1 Preliminary Data

In an IRB-approved study (IRB # 202101103), we enrolled 8 patients undergoing orthopedic surgery to an open-label feasibility phase of the study, to establish recruitment feasibility, and finalize the details pertaining to the study protocol, participant screening, informed consent process, recruitment and follow-up, as well as finalize the workflow for medication optimization and behavioral activation.

Given that these objectives were attained, and reflected in the finalized methodology proposed in this study protocol, we are ready to launch our clinical trial to evaluate the effectiveness and implementation-potential of our intervention bundle using a Hybrid Type 1 Effectiveness-Implementation RCT design.

2.2 Objective

We will test the effectiveness of a bundled intervention comprised of behavioral activation and medication optimization in reducing symptoms of depression and anxiety in older adults undergoing orthopedic surgery (compared with usual care), while examining implementation outcomes. The study will be conducted by the members of the newly established Center for Perioperative Mental Health (CPMH), supported by National Institute of Mental Health (NIMH).

2.3 Patient Selection

The study coordinators will screen the Orthopedic Surgery clinic schedule, as well as the schedule for the Center for Preoperative Assessment and Planning (CPAP) at Barnes-Jewish Hospital for patients undergoing primary hip and knee arthroplasty surgery. Prospective patients will also be identified by Epic workbench reports customized to limit results to those at least 60 years of age with an eligible surgery scheduled at BJH/WUSM, and Barnes-Jewish West County Hospital (BJWCH). In addition, study collaborators, along with orthopedic surgery clinical support team, will refer patients from their clinics who meet the inclusion criteria sought from the EHR. WUSM Orthopedic clinics routinely collect PROMIS data and include that in patients' medical record, and the PROMIS scores for self-reported depression and anxiety symptoms may also be utilized to further customize screening prospect reports.

A member of the study team will obtain the patient's phone number, age and sex, as well as type of surgery, the surgeon's name and planned surgery date through Epic. A member of the study team will contact potentially eligible patients to screen for inclusion criteria.

2.3.1 Inclusion Criteria

Pre-screening:

To be considered for participation, patients will be screened using PHQ-4 questionnaire. Scores ≥3 will indicate positive pre-screen (i.e. can be considered for participation and formal screening). Scores < 3 will indicate negative screen (i.e. patient not considered for formal screening due to low risk for anxiety or depression).

Screening:

Each participant must meet all of the following criteria:

Inclusion	• Adults age≥60 years<br>• Scheduled primary hip or knee arthroplasty procedure<br>• PHQ-ADS (Patient Health Questionnaire Anxiety and Depression Scale) ≥10 indicating clinically significant depression or anxiety symptoms.[7]

2.3.2 Exclusion Criteria

Participants will not be enrolled if any of the following criteria exist:

Exclusion	• Barrier to communication (Unable to read, speak, and understand English)<br>• Severe cognitive impairment screened by the SBT (Short Blessed Test) >10<br>• Acutely suicidal<br>• Considered ineligible per the discretion of the orthopedic surgeon or study PI<br>• Previous participation in this this study or another CPMH feasibility study of the intervention bundle or its feasibility.

2.4. Study Design

All eligible and consenting patients will be randomized in 1:1 ratio (with no stratifications) to receive either the intervention bundle or usual care.
Consenting participants (in both arms) will undergo a standard battery of assessments at baseline (prior to the intervention in the intervention group) and will be followed until approximately 3 months postoperatively. In conjunction with the effectiveness outcomes data, implementation outcomes related to reach, and implementation feasibility, acceptability, and appropriateness of the intervention bundle will be collected.

2.4.1 Study Procedures

Consented subjects will be asked to participate in the study for approximately 3 months after surgery. All participants must sign a consent document (e-consent or paper), indicating their consent, approved by the Washington University School of Medicine Institutional Review Board, before they can participate in the study.

All participants will be asked to complete the following questionnaires at baseline (in person, remotely via a web-based survey, or via telephone):

- CDC HRQOL-14 Quality of Life survey
- PHQ-ADS (Patient Health Questionnaire Anxiety and Depression Scale)
- SBT (Short Blessed Test)
- Presence and intensity of preoperative pain
- Demographics survey
- Patient-reported falls

All participants will be followed for up to approximately 3 months after their scheduled procedure. They will complete follow-up surveys at approximately 1 month and 3 months after surgery by either web-based survey, mail, or via a telephone interview.

At the end of the study, all participants in the intervention arm will be invited to take part in a semi-structured telephone interview conducted by an experienced, qualitative researcher with whom they had no previous contact. The interviews will explore the participants' perceptions, attitudes, and experiences of the intervention bundle.

2.4.2 Minimization of Bias

Enrollment will not be based on sex or ethnic background. Standard of care will not be affected for any of the participants due to study participation. Completion of the outcomes will be entirely patient-reported, to minimize assessor's bias in interpreting patient responses.

2.4.3 Pre-Study Period

Patients who are ≥ 60 years of age undergoing orthopedic surgery will be approached for consent to this study. Eligible patients will be invited to participate in the study, which will be conducted remotely and in-person. Potential candidates for the study will be provided a written and oral description of the study procedures, benefits, and potential risks as well as the opportunity to ask questions regarding the study. Participants are informed that their participation is voluntary and that they may refuse to participate or withdraw from the study at any time without penalty. Patients will be required to give informed consent prior to participation. A study team member who is experienced with cognitive testing will recruit the subjects to the study, and perform the SBT assessment.

Each subject who qualifies for entry into the study on the basis of inclusion/exclusion criteria and completion of informed consent will be assigned the next available patient number. This indicates enrollment in the study. Patients who are eligible but decline participation will be invited to complete an optional survey indicating reasons for decline.

2.4.4. Randomization

The randomization table will be generated by the study biostatistician, using a variable block sequence. The assignment of the treatment condition for each individual participant will be done by the REDCap randomization module after they have been determined to be eligible for the trial.

2.4.5. Blinding

Study staff responsible for administering ratings throughout the study are blinded. This blinding is kept throughout the study until data collection is complete. At the end of the follow-up, the study coordinator will complete a blinding question to try to guess the arm to which each participant was allocated. This will allow to determine whether the blinding procedures in the study were effective [8].

2.4.6 Study Period

Baseline Characterization of Participants: At enrollment, a battery of assessments will characterize participants. *Anxiety and depression* assessment will use the PHQ-ADS, as described above. The use of this tool for this population is supported by a recent meta-analysis suggesting that the PHQ9 (on which this tool is based) has good sensitivity and specificity in the diagnosis of depression in older adults [9]. *Quality of Life* (QoL) will be assessed using the CDC HRQOL-14 survey) [10]. The questions in this patient-reported survey correspond to the QoL domains of Healthy days (physical and mental), Activity Limitations, and Healthy Days Symptoms. *Cognitive function* assessment will be performed using the Short Blessed Test (SBT). SBT is a reliable test, with high inter-rater agreement, which is beneficial for characterizing cognitive functioning in the perioperative setting [11,12]. Recognizing the uncertainty associated with face-to-face data collection due to the COVID-19 pandemic, we selected tools such as the SBT because it can be delivered remotely [13].

All patients in both arms will receive resources (see Handouts for CPMH RCT SOC Group and Handouts for CPMH RCT Intervention Group) either by mail or email if they prefer. The resources include options for exploring brain health, supporting sleep and mental health, and introducing community resources for older adults.

Intervention Bundle: This will be initiated prior to or soon after the surgery and will be continued until approximately 3 months postoperatively.

The basic premise of *behavioral activation* is to help people with mental health problems to engage in activities that are reinforcing or meaningful and guided by their personal values [14]. Patients will be asked to check with their physicians if there is any question about the safety of any physical activities that are included in the behavioral activation plan. Behavioral activation as the core intervention allows for uniformity across participants from each cohort, yet enough flexibility for the actual components of behavioral activation to be individually adapted based on patient preferences. In a recent meta-analysis, behavioral activation programs improved depression and anxiety [15], as well as disability in older adults [16]. In addition, behavioral activation delivered by telehealth to older adults was cost-effective and lowered health care utilization costs over 1 year [17]. In addition to the core intervention of behavioral activation, study participants will tailor the intervention by choosing activities, per their preference, with demonstrated benefit in improving depression and anxiety symptoms in older adults [18,19]. Behavioral activation will generally begin pre-operatively, with sessions approximately every two weeks. After discharge, the behavioral intervention will continue, for 10-12 sessions, or out to approximately 3 months postoperatively. Patients in the intervention arm will receive information (Handouts for CPMH RCT Intervention Group) by mail or email if they prefer. The handouts include tools to support behavioral activation and optional activities.

Medications will be *reviewed and optimized*, as appropriate. In several clinical trials including thousands of older adults, we have developed and refined a process for this review and medication optimization, including adjusting suboptimal dosing for anti-depressants and discontinuing medications that are harmful for brain health (such as anticholinergics [20]). Medication optimization consists of a simple set of principles: reconcile patient's medications, identify the patient's likely need for, and interest in, a medication adjustment, make the adjustment, and assess the response to that adjustment. Benzodiazepines, and strong centrally acting anticholinergics and antihistamines, are the focus of *deprescribing*, as they increase the risk for falls and delirium. We will follow the principles of shared decision-making with patients for medication changes. While the participant is an inpatient, the study pharmacy team will coordinate with the inpatient team to ensure that medication changes that were introduced preoperatively are maintained in-house and that no new inappropriate medications are initiated. After discharge, and up to approximately 3 months postoperatively, the interventionist (also referred to as a perioperative wellness partner) and pharmacy team will ensure that targeted medication changes are reconciled during transitions of care.

Methods

2.4.6 Observations and Measurements

2.4.6.3 Primary Outcome Measures

Effectiveness outcomes will include the primary outcome measure of PHQ-ADS, a validated composite scale of depression and anxiety symptoms [7], measured at 1 and 3 months after surgery. Three months post-operatively is the primary endpoint.

Process measure for target engagement

As a process measure of target engagement at approximately 2 months after surgery, we will use a combined measure including modified versions of the Sinclair Compassion Questionnaire-Short Form (SCQ-SF) [21], Behavioral Activation for Depression Scale (BADS) Activation subscale [22,23], the MiPrep survey [24], and four questions specific to our Wellness Program, and one question to assess participants' understanding of how the study's target medications can affect their mental health, if applicable. The target engagement measure will be collected by survey, which participants in the

intervention group can complete either online via REDCap link or by phone if they prefer, with an unblinded member of the study team.

2.4.6.4 Exploratory Outcome Measures

The exploratory outcomes will include QoL measured via the CDC HRQOL-14 survey, and other perioperative outcomes of interest, nominated by our stakeholders as key outcomes of interest, including:

(1) In-hospital: (i) Delirium incidence,[20] (ii) Length of Stay (hospital and ICU), (iii) information on the use of common medications for chronic pain, such as opioids, gabapentin and pregabalin.

(2) Post-discharge: (i) Falls, (ii) All-cause rehospitalization out to 90 days postoperative, (iii) persistent post-surgical pain (PPSP), (iv) information on the use of common medications for chronic pain, such as opioids, gabapentin and pregabalin.

(3) Process outcomes: For patients on the intervention arm, CAHPS modified survey will be administered after the intervention is complete.

(4) Implementation-potential surveys: Acceptability of Intervention Measure, Intervention Appropriateness Measure, Feasibility of Intervention Measure.

Table 2. Details on Study Outcomes

**Effectiveness Outcomes**		**Data Source**	**Description**	**Data Collection Mechanism**	**Time points (Baseline, 1, 2, 3 month-post-op or EOS)**
**Primary *(Clinical)***	Anxiety and Depression	Patient Health Questionnaire Anxiety and Depression Scale (PHQ-ADS)	A validated composite of symptoms of depression and anxiety	Patient Self-Report	Baseline; 1 mo and 3 mo
Exploratory (Clinical)	Quality of Life	The Health-Related Quality of Life CDC HRQOL-14 survey [10]	A patient-reported global health measure. The questions in this survey correspond to the QOL domains of Healthy days (physical and mental), Activity Limitations, and Healthy Days Symptoms	Patient Self-Report	Baseline; 1 mo and 3 mo

	In-hospital delirium incidence	Retrospective EHR chart review	.	EHR report, and Research Team Member review EHR for delirium-related information from the time of post-op inpatient stay.	In-hospital: at ICU and hospital floor
	Post-discharge falls	Patient Reported Falls	A Patent Reported Falls survey will be collected at 1 month and 3 months either by phone or REDCap link, at follow-up clinic appointments.	Patient Self-Report	Baseline; 1mo and 3mo
	Pain	Pain presence and severity at the site of surgery, and Brief Pain Inventory (BPI) pain interference scale	BPI is a well validated measure of pain severity and pain interference items.	Patient self-report	Baseline, 1 mo, 3 mo
	Length of stay (both hospital and ICU)	Research Data Core	Standardized reports on RDC warehouse will generate statistics of enrolled patients regarding the length of stay, rehospitalization, and related clinical metrics.	EHR report	In-hospital: at ICU and hospital discharge
	All-cause rehospitalization	EHR and patient		EHR, patient	Post-op: 1 mo and 3 mo
**Implementation and Process Outcomes**		**Data Source**	**Description**	**Data Collection Mechanism**	**Time points**
Reach		Enrollment Log	Reach of the study: patients who agreed to participate in the study out of total eligible to participate<br>Reach of the intervention	Electronic Health Record	End of Study

		bundle: patients who completed the interventions out of patients who agreed to participate		
Implementation-Potential (I)	Acceptability of Intervention Measure, Intervention Appropriateness Measure, Feasibility of Intervention Measure	Acceptability, Feasibility and Appropriateness 4-item Likert scale survey	Patient Self-Report	Post-EOS
Patient satisfaction and experience	Modified-Consumer Assessment of Healthcare Providers and Systems (CAHPS)[25]	The modified CAHPS asks patients about their experience with the intervention bundle before surgery, during surgery, and after surgery.	Patient Self-Report	Post-EOS
**Process measure for target engagement**	**Data Source**	**Description**	**Data Collection Mechanism**	**Time points**
Target engagement measure	Combined measure of modified versions of the following scales: Sinclair Compassion Questionnaire-Short Form (SCQ-SF)[21], Behavioral Activation for Depression Scale (BADS) Activation subscale [22], the MiPrep survey[24]	The SCQ-SF is a 5-item patient-reported measure modified to assess compassion of the Wellness Provider; the BADS Activation Subscale (7 items) measures target engagement of BA in studies of geriatric depression [23]; and the MiPrep survey (3 items) assesses patients' preparation for medical interventions.	Patient Self-Report	Approximately 2 mo post-op
**Effectiveness Outcomes**	**Data Source**	**Description**	**Data Collection Mechanism**	**Time points (Baseline, 1, 2, 3 month-**

					**post-op or EOS)**
**Primary *(Clinical)***	Anxiety and Depression	Patient Health Questionnaire Anxiety and Depression Scale (PHQ-ADS)	A validated composite of symptoms of depression and anxiety	Patient Self-Report	Baseline; 1 mo and 3 mo
Exploratory (Clinical)	Quality of Life	The Health-Related Quality of Life CDC HRQOL-14 survey [10]	A patient-reported global health measure. The questions in this survey correspond to the QOL domains of Healthy days (physical and mental), Activity Limitations, and Healthy Days Symptoms	Patient Self-Report	Baseline; 1 mo and 3 mo
	In-hospital delirium incidence	Retrospective EHR chart review	.	EHR report, and Research Team Member review EHR for delirium-related information from the time of post-op inpatient stay.	In-hospital: at ICU and hospital floor
	Post-discharge falls	Patient Reported Falls	A Patent Reported Falls survey will be collected at 1 month and 3 months either by phone or REDCap link, at follow-up clinic appointments.	Patient Self-Report	Baseline; 1mo and 3mo
	Pain	Pain presence and severity at the site of surgery, and Brief Pain Inventory (BPI) pain interference scale	BPI is a well validated measure of pain severity and pain interference items.	Patient self-report	Baseline, 1 mo, 3 mo

	Length of stay (both hospital and ICU)	Research Data Core	Standardized reports on RDC warehouse will generate statistics of enrolled patients regarding the length of stay, rehospitalization, and related clinical metrics.	EHR report	In-hospital: at ICU and hospital discharge
	All-cause rehospitalization	EHR and patient		EHR, patient	Post-op: 1 mo and 3 mo
**Implementation and Process Outcomes**		**Data Source**	**Description**	**Data Collection Mechanism**	**Time points**
Reach		Enrollment Log	<u>Reach of the study</u>: patients who agreed to participate in the study out of total eligible to participate<br><u>Reach of the intervention bundle</u>: patients who completed the interventions out of patients who agreed to participate	Electronic Health Record	End of Study
Implementation-Potential (I)		Acceptability of Intervention Measure, Intervention Appropriatenes s Measure, Feasibility of Intervention Measure	Acceptability, Feasibility and Appropriateness 4-item Likert scale survey	Patient Self-Report	Post-EOS
Patient satisfaction and experience		Modified-Consumer Assessment of Healthcare Providers and Systems (CAHPS)[25]	The modified CAHPS asks patients about their experience with the intervention bundle before surgery, during surgery, and after surgery.	Patient Self-Report	Post-EOS
**Process measure for target <u>engagement</u>**		**Data Source**	**Description**	**Data Collection Mechanism**	**Time points**

Target engagement measure		Combined measure of modified versions of the following scales: Sinclair Compassion Questionnaire-Short Form (SCQ-SF)[21], Behavioral Activation for Depression Scale (BADS) Activation subscale [22], the MiPrep survey[24]	The SCQ-SF is a 5-item patient-reported measure modified to assess compassion of the Wellness Provider; the BADS Activation Subscale (7 items) measures target engagement of BA in studies of geriatric depression [23]; and the MiPrep survey (3 items) assesses patients' preparation for medical interventions.	Patient Self-Report	Approximately 2 mo post-op
**Effectiveness Outcomes**		**Data Source**	**Description**	**Data Collection Mechanism**	**Time points (Baseline, 1, 2, 3 month-post-op or EOS)**
**Primary *(Clinical)***	Anxiety and Depression	Patient Health Questionnaire Anxiety and Depression Scale (PHQ-ADS)	A validated composite of symptoms of depression and anxiety	Patient Self-Report	Baseline; 1 mo and 3 mo
Exploratory (Clinical)	Quality of Life	The Health-Related Quality of Life CDC HRQOL-14 survey [10]	A patient-reported global health measure. The questions in this survey correspond to the QOL domains of Healthy days (physical and mental), Activity Limitations, and Healthy Days Symptoms	Patient Self-Report	Baseline; 1 mo and 3 mo

	In-hospital delirium incidence	Retrospective EHR chart review	.	EHR report, and Research Team Member review EHR for delirium-related information from the time of post-op inpatient stay.	In-hospital: at ICU and hospital floor
	Post-discharge falls	Patient Reported Falls	A Patent Reported Falls survey will be collected at 1 month and 3 months either by phone or REDCap link, at follow-up clinic appointments.	Patient Self-Report	Baseline; 1mo and 3mo
	Pain	Pain presence and severity at the site of surgery, and Brief Pain Inventory (BPI) pain interference scale	BPI is a well validated measure of pain severity and pain interference items.	Patient self-report	Baseline, 1 mo, 3 mo
	Length of stay (both hospital and ICU)	Research Data Core	Standardized reports on RDC warehouse will generate statistics of enrolled patients regarding the length of stay, rehospitalization, and related clinical metrics.	EHR report	In-hospital: at ICU and hospital discharge
	All-cause rehospitalization	EHR and patient		EHR, patient	Post-op: 1 mo and 3 mo
**Implementation and Process Outcomes**		**Data Source**	**Description**	**Data Collection Mechanism**	**Time points**
Reach		Enrollment Log	<u>Reach of the study</u>: patients who agreed to participate in the study out of total eligible to participate<br><u>Reach of the intervention</u>	Electronic Health Record	End of Study

		bundle: patients who completed the interventions out of patients who agreed to participate		
Implementation-Potential (I)	Acceptability of Intervention Measure, Intervention Appropriateness Measure, Feasibility of Intervention Measure	Acceptability, Feasibility and Appropriateness 4-item Likert scale survey	Patient Self-Report	Post-EOS
Patient satisfaction and experience	Modified-Consumer Assessment of Healthcare Providers and Systems (CAHPS)[25]	The modified CAHPS asks patients about their experience with the intervention bundle before surgery, during surgery, and after surgery.	Patient Self-Report	Post-EOS
**Process measure for target engagement**	**Data Source**	**Description**	**Data Collection Mechanism**	**Time points**
Target engagement measure	Combined measure of modified versions of the following scales: Sinclair Compassion Questionnaire-Short Form (SCQ-SF)[21], Behavioral Activation for Depression Scale (BADS) Activation subscale [22], the MiPrep survey[24]	The SCQ-SF is a 5-item patient-reported measure modified to assess compassion of the Wellness Provider; the BADS Activation Subscale (7 items) measures target engagement of BA in studies of geriatric depression [23]; and the MiPrep survey (3 items) assesses patients' preparation for medical interventions.	Patient Self-Report	Approximately 2 mo post-op

In addition to these outcomes, we will assess fidelity to our intervention bundle to ascertain the extent to which an intervention bundle is carried out, as intended and consistently across different settings, and patients. If the patient agrees to audio/video recording, the intervention sessions will be

audio recorded (or audio/video if the patient prefers to meet by Zoom teleconference). To track this, we will use the recordings of these discussions between the patient and the perioperative wellness partner during the behavioral intervention and medication optimization sessions. We will capture data on patient interactions with the wellness partner, their delivery of the intervention bundle, any observed barriers and facilitators to the intervention adherence, acceptance and fit within the perioperative workflow. 10% of these sessions of patients and their wellness partners will be selected based on a convenience sample for evaluating fidelity of the bundle.

Lastly, we will also obtain stakeholder perspectives on the intervention bundle after the intervention period has been completed using semi-structured interviews. We will conduct interviews with patients who participated in the intervention, and the topics of discussions will be guided by an implementation science framework referred to as CFIR (Consolidated Framework of Implementation Research). The interviews will explore the participants' perceptions, attitudes, and experiences with the intervention bundle, intervention bundle acceptability and detailed accounts of participants' experiences after the intervention has been stopped with regards to intervention sustainability and maintenance. Interviews will be conducted via Zoom or telephone and will be digitally recorded and transcribed verbatim.

Study Calendar

<table>
<tr><th></th><th>Time 0</th><th>Baseline / Time 1</th><th>1 mo follow up</th><th>2 mo follow up</th><th>3 mo follow up</th><th>Post-EOS</th></tr>
<tr><td>Patient Consent</td><td>X</td><td></td><td></td><td></td><td></td><td></td></tr>
<tr><td>Patient Screening</td><td>X</td><td></td><td></td><td></td><td></td><td></td></tr>
<tr><td>Patient Baseline surveys</td><td></td><td>X</td><td></td><td></td><td></td><td></td></tr>
<tr><td>Patient Follow-up 1 & 3 mo Surveys</td><td></td><td></td><td>X</td><td></td><td>X</td><td></td></tr>
<tr><td><u>For Patients on the Intervention Arm</u>: Intervention-Patient Interviews and Implementation, Patient Satisfaction and Experience Surveys</td><td></td><td></td><td></td><td></td><td></td><td>X</td></tr>
<tr><td><u>For Patients on the Intervention Arm</u>: Process Target Engagement Measure</td><td></td><td></td><td></td><td>X</td><td></td><td></td></tr>
<tr><td><u>For Patients on the Intervention Arm</u>: Behavioral Activation and Medication Review Intervention Sessions</td><td></td><td></td><td colspan="3">Approximately 8-12 sessions as needed (per Pt preference) after Baseline, continuing for up to approximately 3 months post-op.</td><td></td></tr>
</table>

2.4.6.5 Statistical Methods

Analyses were conducted in a modified intention-to-treat population, including all randomized participants except those whose surgery was canceled after randomization. The prespecified statistical analysis plan is available in the Statistical Analysis Protocol (Supplement 1 below). Internal consistency (Cronbach α) was calculated for the PHQ-9 and GAD-7 at baseline and 3 months within each cohort.

The primary outcome was analyzed separately for each cohort using repeated-measures mixed models with fixed effects for treatment group, time (baseline, 1 month, and 3 months), and their

interaction. Least-squares mean changes from baseline to 3 months were compared between groups, with sensitivity analyses adjusting for baseline values. A prespecified, individual-patient, 2-stage, fixed-effect meta-analysis summarized treatment effects across the three cohorts. This is logically equivalent to a single fixed-effect model in which the study group is a fixed effect and the inference is whether there is a mean intervention effect in these three specific groups taken as a whole.

Other outcomes measured longitudinally were analyzed using analogous mixed models. Length of stay was analyzed using analysis of covariance; delirium, rehospitalization, and persistent postsurgical pain using modified Poisson regression with a log link; and falls using repeated-measures mixed models adjusted for baseline. Prespecified covariates were included where appropriate, and potential effect modification by sex was examined. Analyses were performed using SAS, version 9.4M8 (SAS Institute Inc, Cary, NC, USA).

All models were adjusted for sex assigned at birth, surgery type based on complexity, invasiveness, and length of recovery, and ASA (American Society of Anesthesiologists) physical status score, a validated measure of perioperative risk based on medical comorbidities that effectively discriminates between low- and high-risk patients. All pain models additionally included chronic pain and opioid use as covariates. A meta-analytical approach was used to allow the effects of covariates to differ across surgical cohorts. This approach also accommodated potential differences in residual error variance across cohorts, resulting in simpler models. It was particularly appropriate because the distributions of surgery type were not equivalent across the surgical cohorts.

2.4.6.6 Sample Size and Power of Primary Analysis:

In order to estimate the power of the primary outcome analysis we examined the manuscript which provided the original validity and reliability studies for the PHQ-ADS [26] and one of the trials which provided data for that study (SCOPE) [27,28]. At 3 months they observed a change from baseline divided by the standard deviation of the change of 0.43 which corresponds to a change of 3 points on the PHQ-ADS. The PHQ-ADS authors estimate the minimal important clinical difference (MICD) from the standard error of measure (SEM) as 3-4 points. If we postulate a difference in the change scores of 2 points between the two groups, then with a power of 0.80 we would need 36 in each group. Since the SCOPE trial was a pain relief study without a requirement of anxiety or depression, we consider these estimates rough estimates. This trial will target having 100 mITT patients (50 per arm) in order to account for any incomplete data among recruited participants. Recruitment will continue until 100 randomized patients have had their procedure or surgery.

2.5 Management of Intercurrent Events

2.5.1 Adverse Experiences

Based on the nature of this study, which involves surveys, a cognitive test, and a behavioral intervention, adverse events are exceedingly unlikely.

2.5.2 Premature Withdrawal

Subjects will be withdrawn if the investigator decides that discontinuation is in the best interest of the subject, or the subject requests withdrawal from the study. Additionally at PI discretion, they may be withdrawn if their surgery is cancelled or postponed (for clinical or non-clinical reasons) for more than approximately 3 months after baseline.

Participants who choose to end study participation early, may be asked to complete early termination assessments. Additionally, those in the intervention arm may choose to withdraw from that at any time, but will be allowed to continue with data collection follow-ups if they still want to participate.

2.5.3 Potential Risks

This study involves minimal risk to subjects. Unlikely but potential risks include increased risk of depression or anxiety, errors in medication recommendations or medication withdrawal symptoms, and breach of confidentiality. The risk of medication withdrawal (i.e., from benzodiazepines) is mitigated by slowly tapering rather than stopping these medications. Patients who are identified as being acutely suicidal will be excluded from the study. Nevertheless, since the rate of completed suicide in the USA remains high (i.e., about twice the rate of homicide) and most Americans who commit suicide suffer from depression, all participants eligible to participate in this study are statistically at a relatively higher risk for suicide than the general population. However, the participants' absolute risk for completing suicide during this brief study remains very low (i.e., about 1 in 3,000 to 10,000) and participation in the study does not create or increase the risk of completed suicide; rather, most experts believe that one of the most efficient ways to decrease suicidal risk in older depressed individuals is to treat their depression. Furthermore, all participants will be formally assessed frequently throughout the study. If the study personnel identify that a participant has become acutely suicidal, this participant will be referred to a mental health professional for further evaluation and treatment (see section: Procedures to Minimize Risks). This may lead to a clinical intervention that is lifesaving and may not have occurred had the participant not been participating in the study.

2.5.4 Procedures to Minimize Potential Risks

Medication Error Risk: Dr. Eric Lenze will supervise careful training and oversite of the intervention pharmacists making medication optimization recommendations to reduce the risk of medication error or medication withdrawal symptoms. Algorithms for tapering medications that should not be abruptly discontinued will be employed. We have carried out this deprescribing protocol in several large RCTs and have not seen any problems occur.

Suicide risk management: As discussed above, at each assessment point, the research staff will assess for passive death wish, and suicidal ideation, intent or plan when they administer the PHQ-ADS. If a participant endorses suicidal ideation, intent, or plan, the rater will be trained to follow an operationalized protocol that has been developed to manage high-risk participants in other studies of depressed participants potentially at risk for suicide. This protocol has already been used successfully by Dr. Lenze's team to manage acutely suicidal patients. Briefly, the protocol entails a specific determination of the suicidal risk and prescribes a set of actions. For instance, when a participant is determined to be at high and immediate risks, the rater is instructed to stay with the participant until he or she has contacted a study psychiatrist (Dr. Lenze) to discuss the situation and to devise a plan. For this reason, raters will have cell phones and Dr. Lenze will be reachable via email and cell phone. In case of extreme emergency, raters are instructed to call their hospital security team or 911 for immediate help and to initiate commitment proceedings.

Patients will be asked to check with their clinician if there is any question about the safety of any physical activities that are included in the behavioral activation plan.

The risk of breach of confidentiality will be minimized by ensuring that protected health information will only be shared with members of the research team. Only data necessary to complete the study will be collected. Electronic data will be kept in a password-protected electronic database only

assessable via password protected departmental computers. Patients may choose to not participate in the study or withdraw at any time with no penalty in terms of the care they receive.

2.5.5 Data and Safety Monitoring Plan

The specific monitoring plan for this investigation is commensurate with the risks and the size and complexity of the studies planned. Given the nature of the protocol, the risks are likely limited to breach of confidentiality. The Principal Investigator will be responsible for monitoring the data and assuring protocol compliance. The PI will meet with the clinical research coordinator and Data Manager bi-weekly to review enrollment, eligibility, participant follow-up and outcome assessment. The PI and study coordinator will monitor for breaches of confidentiality on an ongoing basis. Study-related adverse events will be reported per IRB guidelines. All serious adverse events (SAE) will be recorded and adjudicated by a CPMH Principal Investigator and reported per IRB guidelines. Any endorsement of suicidal ideation will be managed acutely as detailed in the section 2.5.4. If study staff becomes aware of a reportable adverse event, it will be reported to necessary stakeholders according to Washington University Human Protection Office guidelines. This type 1 hybrid randomized study does not require a Data and Safety Monitoring Board.

3. HUMAN SUBJECTS RESEARCH

3.1 Protection of Human Subjects

The study will be conducted under appropriate Washington University Institutional Review Board protocols and consent forms approvals. The study will be conducted under the supervision of the PI, who has extensive of experience in the conduct of human research studies.

3.2 Sources of Materials

Preoperative assessments along with preoperative and postoperative questionnaires regarding quality of life, depression, and anxiety will be obtained from all study participants. The results of follow-up questionnaires will be obtained via e-mail, mail, or telephone survey. Data will be collected at baseline, one, two, and 3 months following surgical procedure.

3.2.1 List of Protected Health Information Collected for Study

The HIPAA protected identifiers that will be collected for this study include:

- Name
- Dates
  - Date of birth
  - Date of services (surgical procedure)
- Contact information
  - Mailing address
  - Phone number
  - Electronic mail address
- Medical record number

3.2.2 Data Management

All paper data will be stored under lock and key (office, file cabinet) and all electronic data will be stored in REDCap, only accessible to the research team members. Surveys that are completed electronically will be administered via the REDCap server. All REDCap email correspondence will be sent using secure survey links. All survey information will be coded with the patient's unique study ID number and no personal health information that may directly identify patients will be included.

3.3 Recruitment and Informed Consent

Participants will be recruited either from the orthopedic surgery clinics at Washington University or the Center for Preoperative Assessment and Planning (CPAP) at Barnes-Jewish Hospital/Washington University. Participants with pre-screening scores of PHQ-4 ≥3 will be recruited, and eligibility confirmed if PHQ-ADS ≥10. Retention strategies will include flexibility in scheduling follow-up visits and coordination of research visits with any in-person clinical visits. Subjects will be given verbal and then written descriptions of the study aims, procedures, risks, and benefits, and will be required to give informed consent. A member of the research team will be available to present study descriptions, informed consent, and answer any questions. Subjects are informed verbally and in writing that participation is voluntary and they may refuse to participate and may withdraw from the study at any time without penalty. Remuneration for each data collection study visit will be provided to encourage retention as well.

3.4 Potential Benefits of the Proposed Research to the Subjects and Others

Although the study is designed to improve symptoms of depression and anxiety and other outcomes of care, there is no guarantee that the study participants will receive any clinical benefits by participating in the study. This may lead to a clinical intervention that is lifesaving and may not have occurred had the participant not been participating in the study. Given the undertreatment of depression and anxiety in older adults, the minimal risks are acceptable in comparison to the potential benefits in improving the mental health of older adults undergoing surgery.

3.5 Remuneration

Patients will receive remuneration by a reloadable debit card of $25 for completion of the following data collection study visits: Standard Care (control arm) participants will be compensated for their time at Baseline, 1, and 3 month post-op, up to $75 for completion of all data collection time points. Wellness Program (intervention arm) participants will be compensated for their time at Baseline, 1, and 3 month post-op, plus a post-EOS semi-structured interview, so up to $100 for completion of all eligible data collection time points.

3.6 Inclusion of Women

Eligible individuals will be offered the opportunity to participate in the study regardless of gender or minority status. Thus, the sample composition will reflect the composition of community-living older adults in the St. Louis area.

3.7 Inclusion of Minorities

All of our studies actively encourage the participation of minorities in the research. Our minority recruiting typically matches the demographic composition of the Washington University community from which subjects will be recruited (78% white, 21% Black, <1 % Hispanic).

3.8 Inclusion of Children

We will not be including children in this study.

4. REFERENCES

1. Murphy L, Helmick CG. The impact of osteoarthritis in the United States: a population-health perspective. *Am J Nurs.* 2012;112(3 Suppl 1):S13-19.
2. Kurtz S, Ong K, Lau E, Mowat F, Halpern M. Projections of primary and revision hip and knee arthroplasty in the United States from 2005 to 2030. *J Bone Joint Surg Am.* 2007;89(4):780-785.
3. Singh JA, Lewallen DG. Predictors of activity limitation and dependence on walking aids after primary total hip arthroplasty. *J Am Geriatr Soc.* 2010;58(12):2387-2393.
4. Wylde V, Bruce J, Beswick A, Elvers K, Gooberman-Hill R. Assessment of chronic postsurgical pain after knee replacement: a systematic review. *Arthritis Care Res (Hoboken).* 2013;65(11):1795-1803.
5. Manley M, Ong K, Lau E, Kurtz SM. Total knee arthroplasty survivorship in the United States Medicare population: effect of hospital and surgeon procedure volume. *J Arthroplasty.* 2009;24(7):1061-1067.
6. Roberts VI, Esler CN, Harper WM. A 15-year follow-up study of 4606 primary total knee replacements. *J Bone Joint Surg Br.* 2007;89(11):1452-1456.
7. Duivenvoorden T, Vissers MM, Verhaar JA, et al. Anxiety and depressive symptoms before and after total hip and knee arthroplasty: a prospective multicentre study. *Osteoarthritis Cartilage.* 2013;21(12):1834-1840.
8. Dave AJ, Selzer F, Losina E, et al. The association of pre-operative body pain diagram scores with pain outcomes following total knee arthroplasty. *Osteoarthritis Cartilage.* 2017;25(5):667-675.
9. Bay S, Kuster L, McLean N, Byrnes M, Kuster MS. A systematic review of psychological interventions in total hip and knee arthroplasty. *BMC Musculoskelet Disord.* 2018;19(1):201.
10. Abraham, J., et al., *A Qualitative Study of Perioperative Depression and Anxiety in Older Adults.* Am J Geriatr Psychiatry, 2020.
11. Kroenke, K., et al., *Patient Health Questionnaire Anxiety and Depression Scale: Initial Validation in Three Clinical Trials.* Psychosom Med, 2016. **78**(6): p. 716-27.
12. Ritchie, J. and J. Lewis, *Qualitative research practice: a guide for social science students and researchers*. 2003, London: Sage Publications.
13. Freed, B., et al., *Assessing blinding in trials of psychiatric disorders: a meta-analysis based on blinding index.* Psychiatry Res, 2014. **219**(2): p. 241-7.
14. Levis, B., et al., *Accuracy of Patient Health Questionnaire-9 (PHQ-9) for screening to detect major depression: individual participant data meta-analysis.* BMJ, 2019. **365**: p. l1476.
15. Mielenz, T.J., L.F. Callahan, and M.C. Edwards, *Item response theory analysis of Centers for Disease Control and Prevention Health-Related Quality of Life (CDC HRQOL) items in adults with arthritis.* Health Qual Life Outcomes, 2016. **14**: p. 43.

16. Gregory, S.H., et al., *Abnormal preoperative cognitive screening in aged surgical patients: a retrospective cohort analysis.* Br J Anaesth, 2021. **126**(1): p. 230-237.

17. Oughli, H.A., et al., *Cognitive Improvement in Older Adults in the Year After Hip Fracture: Implications for Brain Resilience in Advanced Aging.* Am J Geriatr Psychiatry, 2018. **26**(11): p. 1119-1127.

18. Arias, F., et al., *Rapid In-Person Cognitive Screening in the Preoperative Setting: Test Considerations and Recommendations from the Society for Perioperative Assessment and Quality Improvement (SPAQI).* Perioper Care Oper Room Manag, 2020. **19**.

19. Puspitasari, A.J., et al., *A randomized controlled trial of an online, modular, active learning training program for behavioral activation for depression.* J Consult Clin Psychol, 2017. **85**(8): p. 814-825.

20. Stein, A.T., et al., *Looking beyond depression: a meta-analysis of the effect of behavioral activation on depression, anxiety, and activation.* Psychol Med, 2020: p. 1-14.

21. Choi, N.G., et al., *Improving Social Connectedness for Homebound Older Adults: Randomized Controlled Trial of Tele-Delivered Behavioral Activation Versus Tele-Delivered Friendly Visits.* Am J Geriatr Psychiatry, 2020.

22. Egede, L.E., et al., *Cost-Effectiveness of Behavioral Activation for Depression in Older Adult Veterans: In-Person Care Versus Telehealth.* J Clin Psychiatry, 2018. **79**(5).

23. Farhang, M., et al., *Impact of mind-body interventions in older adults with mild cognitive impairment: a systematic review.* Int Psychogeriatr, 2019. **31**(5): p. 643-666.

24. Lenze, E.J., et al., *Mindfulness-based stress reduction for older adults with worry symptoms and co-occurring cognitive dysfunction.* Int J Geriatr Psychiatry, 2014. **29**(10): p. 991-1000.

25. Wildes, T.S., et al., *Effect of Electroencephalography-Guided Anesthetic Administration on Postoperative Delirium Among Older Adults Undergoing Major Surgery: The ENGAGES Randomized Clinical Trial.* JAMA, 2019. **321**(5): p. 473-483.

26. Lamb, S.E., et al., *Development of a common outcome data set for fall injury prevention trials: the Prevention of Falls Network Europe consensus.* J Am Geriatr Soc, 2005. **53**(9): p. 1618-22.

27. Manos, R.C., J.W. Kanter, and W. Luo, *The behavioral activation for depression scale-short form: development and validation.* Behav Ther, 2011. **42**(4): p. 726-39.

28. Quality, A.f.H.R.a., *About CAHPS*, AHRQ, Editor. 2020: Rockville, MD.

29. Andersen, P.K. and R.G. Gill, *Cox's Regression Model for Coounting Processes: A Large Sample Study.* Ann Statist, 1982. **10**(4): p. 1100-1120.

30. Kroenke, K., et al., *The Patient Health Questionnaire Anxiety and Depression Scale (PHQ-ADS): Initial Validation in Three Clinical Trials.* Psychosom Med, 2016. **78**(6): p. 716-727.

31. Kroenke, K., et al., *Stepped Care to Optimie Pairn Care Effectiveness (SCOPE) Trial: study design and sample characteristics.* Contemp Cln Trials, 2013. **34:**: p. 270-281.

32. Kroenke, K., et al., *Telecare collaborative management of chronic pain in primary care: a randomized clinical trial.* JAMA, 2014. **312**(3): p. 240-248.

STATISTICAL ANALYSIS PLAN

Perioperative mental health intervention for depression and anxiety in older adults undergoing surgery

Version: 3

Authors: Michael Yingling, Sr Statistical Data Analyst
J. Philip Miller, Professor of Biostatistics
Torie Gettinger, Assistant Professor of Psychiatry
Yi Zhang, Sr Statistical Data Analyst
Katherine Holzer, Assistant Professor of Anesthesiology
Simon Haroutounian, Associate Professor of Anesthesiology
Ryan Calfee, Professor of Orthopedic Surgery
Benjamin Kozower, Professor of Surgery
Joanna Abraham, Professor of Anesthesiology
Michael Avidan, Professor of Anesthesiology
Eric Lenze, Professor of Psychiatry

Date: April 2025

Table of Contents

SECTION	PAGES
1. INTRODUCTION	68
2. ANALYSIS OBJECTIVES	68
3. ANALYSIS SETS/POPULATIONS/SUBGROUPS	68
4. DATA SOURCE	69
5. RANDOMIZATION	72
6. BLINDING	72
7. MINIMIZATION OF BIAS	72
8. ENDPOINTS AND COVARIATES	72
9. HANDLING OF MISSING VALUES AND OTHER DATA CONVENTIONS	72
10. STATISTICAL PROCEDURES	72
10.1 Primary/Other Outcome Analyses	
10.2 Subgroup Analyses	
10.3 Exploratory Analyses	
10.4 Power Analysis	
11. QUALITY CONTROL PLANS	75
12. PROGRAMMING PLANS	75
13. FUNDING AND ACKNOWLEDGEMENTS	75
14. REFERENCES	75

Abbreviations

CPMH	Center for Perioperative Mental Health
NIMH	National Institute of Mental Health
BA	Behavioral Activation
MO	Medication Optimization
RCT	Randomized Controlled Trial
PHQ-ADS	Patient Health Questionnaire Anxiety and Depression Scale
mITT	Modified Intent-to-Treat
BPI	Brief Pain Inventory
PPSP	Persistent Postsurgical Pain

1 INTRODUCTION

Inadequate management of preoperative mental health disorders often contributes to poor postoperative outcomes[1], including increased rates of readmission, delirium, falls, and mortality[3-5,29]. However, very little work has been done to improve perioperative mental health. In particular, there have not been any systematic efforts to identify evidence-based behavioral interventions that were originally developed for depression and anxiety in otherwise medically well psychiatric patients, adapt them for use in surgical populations, test their effectiveness in these patient populations, and study implementation strategies.

The proposed study will focus on comparing a perioperative mental health intervention of behavioral therapy focused on Behavioral Activation (BA), plus Medication Optimization (MO), compared to enhanced usual care, among older adults undergoing cardiac, orthopedic or oncologic procedures with anesthesia (N=300; n=100 each surgical type). We chose to use enhanced usual care to provide some harmonization between the participants in the usual care arm. Providing resources to participants in both arms increases the possibility of benefit for all participants, which may potentially improve recruitment and retention.

We hypothesize that the mental health intervention can mitigate anxiety and depression symptoms during the perioperative period[30]. Towards this end, we propose to test the effectiveness as well as the implementation potential of the intervention in a Hybrid Type 1 Effectiveness-Implementation RCT Design.

This study includes adults aged 60 and older with current depression/anxiety symptoms (PHQ-ADS ≥10) and that are undergoing major orthopedic, cardiac or oncologic procedures. Each patient is randomized to the perioperative mental health intervention vs enhanced usual care.

2 ANALYSIS OBJECTIVES

Aim 1. Examine the effects of a perioperative mental health intervention, composed of a wellness program and medication optimization, on depression/anxiety symptoms compared to enhanced usual care in patients undergoing a scheduled cardiac, orthopedic or oncologic surgical procedure.

H1: A greater decrease in depression/anxiety symptoms will occur in patients randomized to the perioperative mental health intervention compared to patients randomized to enhanced usual care.

3 ANALYSIS SETS/ POPULATIONS/SUBGROUPS

We will randomize older adults aged 60 to either the perioperative mental health intervention or enhanced usual care. The modified intent-to-treat analyses will include all patients that underwent an orthopedic, cardiac or oncologic surgical procedure. The goal target in the mITT group is 300 patients, with approximately 100 patients in each surgical cohort. Patients can choose to refuse any component of this study.

4 DATA SOURCE

Below is a schedule of outcome measures.

**Effectiveness Outcomes**		**Data Source**	**Description**	**Data Collection Mechanism**	**Time points**
Primary (Clinical)	3-month change in anxiety and/or depression symptoms	PHQ-ADS[31]	A validated composite of symptoms of depression and anxiety	Patient self-report	Baseline; 1mo and 3 mo
Other (Clinical)	1- and 3-month change in quality of life	CDC HRQOL-14 survey[32]	A patient-reported global health measure. The questions in this survey correspond to the QOL domain of Healthy Days Symptoms	Patient self-report	Baseline; 1 mo and 3 mo
	In-hospital delirium incidence	Retrospective EHR chart review	The Chart-based Delirium Identification Instrument (CHART-DEL)[33] will be used by trained research assistants.	EHR report, and research team member review EHR for delirium-related information from the time of post-op inpatient stay.	In-hospital: at ICU and hospital floor
	Falls	Patient reported falls	Modified patient reported falls survey[34]	Patient self-report	Baseline; 1mo and 3mo
	Persistent post-surgical pain (PPSP)	Pain presence and severity at the site of surgery, and	PPSP/BPI are well-validated measures of pain severity and pain interference items	Patient self-report	1 mo, 3 mo

		Brief Pain Inventory (BPI)[35] pain interference scale			
	Length of stay (both hospital and ICU)	Research data core	Standardized reports on research data core warehouse will generate statistics of enrolled patients regarding the length of stay, rehospitalization, and related clinical metrics.	EHR report	In-hospital: at ICU and at hospital discharge
	All-cause rehospitalization	Research data core		EHR report	Post-op: 1 mo and 3 mo
**Implementation and Experience Outcomes**		**Data Source**	**Description**	**Data Collection Mechanism**	**Time points**
Reach		Enrollment Log	Reach of the study: proportion and representativeness of patients who agreed to participate in the study out of total eligible to participate[36] Reach of the perioperative mental health intervention*: proportion of participants who completed the intervention out of participants who agreed to participate.	EHR and study data	EOS
Intervention acceptability*		Acceptability of Intervention Measure[37]	4-item Likert scale survey measuring whether the interventions are agreeable to surgical older patient participants.	Patient self-report	EOS

Intervention feasibility*	Feasibility of Intervention Measure[37]	4-item Likert scale survey measuring whether the interventions can be successfully carried out in perioperative settings.	Patient self-report	EOS
Intervention appropriateness*	Intervention Appropriate-ness Measure[37]	4-item Likert scale survey measuring the perceived fit of the interventions by the participants to address perioperative anxiety and/or depression symptoms.	Patient self-report	EOS
Patient satisfaction and experience*	Modified-CAHPS[38]	The modified CAHPS asks patients about their experience with the intervention before surgery, during surgery, and after surgery.	Patient self-report	EOS

Note. PHQ-ADS = Patient Health Questionnaire Anxiety and Depression Scale; CDC HRQOL = Centers for Disease Control and Prevention Health-Related Quality of Life survey; EHR = electronic health record; ICU = intensive care unit; CAHPS = Consumer Assessment of Healthcare Providers and Systems survey; EOS = end of study; *Applicable to intervention arm participants only

5 RANDOMIZATION

All eligible and consented participants will be randomized in a 1:1 ratio to receive either the perioperative mental health intervention or enhanced usual care. The study biostatistician generates the randomization table using a variable block sequence. The assignment of the treatment condition for each individual participant will be done by the REDCap® randomization module[39,40] after confirming trial eligibility. For participants assigned to the intervention, a randomization table will be generated using a similar block sequence design, and the data manager will manually select the individual intervention team member according to the randomization table and record that information in REDCap®. This process will trigger an automatic notification via REDCap® to the assigned interventionists.

6 BLINDING

Research coordinators responsible for collecting outcome data throughout the study will be blinded until data collection is complete. At the end of the 3-month follow-up, the research coordinators will complete a blinding question to guess the arm to which each participant was allocated. This will allow the team to determine the effectiveness of the blinding procedures [41]. Upon completing this form, it will be locked by the data manager, and the participant's assignment will be revealed to the research coordinators to conduct the end-of-study interviews with the participants in the intervention arm.

7 MINIMIZATION OF BIAS

Enrollment will not be based on sex or ethnic background. Standard Care will not be affected for any of the patients due to study participation. Completion of the outcomes will be entirely patient-reported, to minimize the assessor's bias in interpreting patient responses.

8 ENDPOINTS AND COVARIATES

Primary outcome
The primary outcome of the study is the change in PHQ-ADS from baseline to 3-month visit. All models will be adjusted for covariates which strongly associate with the outcome. These covariates include sex at birth, surgery type, and ASA (American Society of Anesthesiologists) score. In addition, all pain models will include the additional covariates of chronic pain and opioid use.

9 HANDLING OF MISSING VALUES AND OTHER DATA CONVENTIONS

The data is regularly reviewed to identify missing data. All models will be fit with SAS or R using a maximum likelihood criteria which utilizes all available information yielding optimal solutions in the face of missing data as long as it is missing at random. Multiple imputation will be used where the statistical model does not already provide appropriate protection for data missing at random.

- For the clinical assessments, if the scale consisted of 6-10 items and a participant is missing one item, then the average of the other items will be used to fill in the missing item. If more than 1 item is missing, then that participant's data will not be used for that scale. For a 1-5 item scale, any missing data will exclude that participant's data for that scale. For scales with greater than 10 items, a 10% cutoff rule will be employed for missing data.

10 STATISTICAL PROCEDURES

10.1 Primary Outcome Analyses

The primary outcome of the study is the change in PHQ-ADS from baseline to 3-month visit. In each surgical cohort, a mixed model repeated measures ANOVA with a treatment group by time point design will be run to determine the overall impact of the intervention on the change in the PHQ-ADS. The results will be synthesized using a prespecified, individual patient, two-stage meta-analysis of the three trials[42], and the plan is for this to be the primary analytic strategy to demonstrate effectiveness of the intervention across all surgical subgroups. This is logically equivalent to a single fixed-effect model where the study group is a fixed-effect and the inference is whether there is an average intervention effect in these three specific groups taken as a whole.

The population used for the primary analysis is a modified Intent to Treat (mITT) group consisting of those randomized participants who actually undergo the surgery (ie, for randomized participants in whom the surgery is cancelled or indefinitely delayed, they are not part of the analysis sample). Participants will still be included in the analysis if they died post-operatively or were lost to follow-up, prior to the end of the trial. Statistical significance will be set at 0.05 and relevant changes in PHQ-ADS will be estimated along with 95% confidence intervals. Any data available at baseline, 1 month or 3 months are included in the repeated measures model. The three-month response is the primary outcome and is tested with an appropriate contrast to compare the change between the two groups from baseline to 3 months.

Implementation outcomes

For the implementation outcomes, we will present the means and standard deviations for each of the implementation surveys (acceptability, feasibility, and appropriateness of the interventions) and the CAHPS patient experience survey. Study reach will be presented as the proportion of patients who agreed to participate in the study out of the total eligible patients to participate. Intervention reach will be reported as the proportion of participants who completed the perioperative mental health intervention out of participants who agreed to participate.

Intervention process variables

We will report the following averages: number of wellness program sessions, duration of sessions, number of preoperative sessions, number of postoperative sessions, and session modality. Additionally, for the medication optimization, we will report averages for number of medications at baseline and the number of medications eligible for optimization (de-prescribing and dose escalation). By including the number of sessions as a covariate in each cohort's model used in the primary analysis we can examine the relationship between the number of sessions and the change in PHQ-ADS which will allow us to determine the minimum number of sessions needed to ascertain the initial efficacy. This will be used to inform the development of a fully powered RCT. We will assess multiple components of fidelity to the perioperative mental health intervention to examine the extent to which the intervention is carried out by our intervention team as intended and consistently across different settings. We will report summary information for outcomes of optimization. Regarding the intervention fidelity, we will report summary results of the fidelity checklist in the areas of treatment delivery, treatment receipt, and enactment of treatment skills for both the wellness program and medication optimization, including the proportion of participants who followed the components of the intervention as intended.

Target engagement

We will conduct a survey at 2 months after surgery to measure target engagement of our wellness program and medication optimization. The survey contains questions on the various components of our intervention and includes the Behavioral Activation for Depression (BADS) activation subscale,[22] the Sinclair Compassion Questionnaire[43] to evaluate the level of compassion and attention provided by the interventionists, the MiPrep scale[24] to determine the level at which participants felt prepared for surgery, and one question to assess participants' understanding of how the study's target medications can affect their mental health, if applicable. There are 5 response options for each question ranging from strongly

disagree to strongly agree. This survey will be used to assess the components of the intervention believed to be responsible for the change in depression and anxiety over the course of the study period.

10.2 Sub-Group Analyses

Participants who are not taking a target medication will only receive the behavioral therapy component of our intervention. We will analyze these participants in a sub-group analysis, using the same mixed model described in the primary outcome analysis to evaluate the effect of the behavioral therapy component only. This is exploratory to examine the effect size of the behavioral component by itself.

10.3 Other Exploratory Analyses

Other outcomes measured on the same schedule as the PHQ-ADS will be analyzed utilizing the same mixed model described in the primary outcome analysis. For length of stay (ICU and hospital), a simple analysis of covariance will compare the two groups where we will identify the relevant covariates during the planning phase. Delirium, re-hospitalization and meaningful persistent postsurgical pain will use a modified Poisson model with a log link function and appropriate covariates. Falls data will be analyzed first by providing a frequency table of the severity of the fall for each group (Intervention Vs Usual Care). Then, a repeated measures mixed-model will be employed to assess the effects of time and group on the total number of falls. Finally, fall severity will be assessed using a repeated measures ordinal multinomial logistic regression. For these last two models, the baseline value will be treated as a covariate. For all analyses, we will examine the potential moderating effect of patients' sex. The statistical analysis plan will be finalized prior to the analysis of the trials.

10.4 Power

We based sample size on the need to have adequate power to examine intervention effects within each of the individual surgical subgroups (n=100 each), as well as the planned meta-analysis (N=300). To estimate the power, we examined the manuscript that provided the original validity and reliability studies for the PHQ-ADS[44] and one of the trials that provided data for that study (Stepped Care to Optimize Pain care Effectiveness (SCOPE)).[45,46] The PHQ-ADS authors estimate the minimal clinically important difference (MCID) from the standard error of measure as 3-4 points.[44] Since the SCOPE trial was a pain relief study without a requirement for anxiety or depression, we conducted a blinded sample size reassessment after 125 participants had completed the 3-month assessment in the 3 cohorts. The average change in the PHQ-ADS was 6.7 with a standard deviation of 8.8 (d= 0.76). These 125 subjects were from 135 in the mITT group, or 7% dropout. With the chosen sample size of 50 mITT subjects in each group, we estimated the power for a simple t-test between the two groups of size 46, a 2-tailed significance level of 0.05, and a difference between the two groups of >4 points as shown in figure 1 below. These power calculations are somewhat conservative as they are based on a simple t-test and we will be using a repeated measures ANOVA for the actual analysis.

Figure 1: Power calculation curve suggesting a power of at least 55% for groups of size 46, calculated from a mITT sample size of 50 in each group with a 7% dropout, and a difference between groups >4 points.

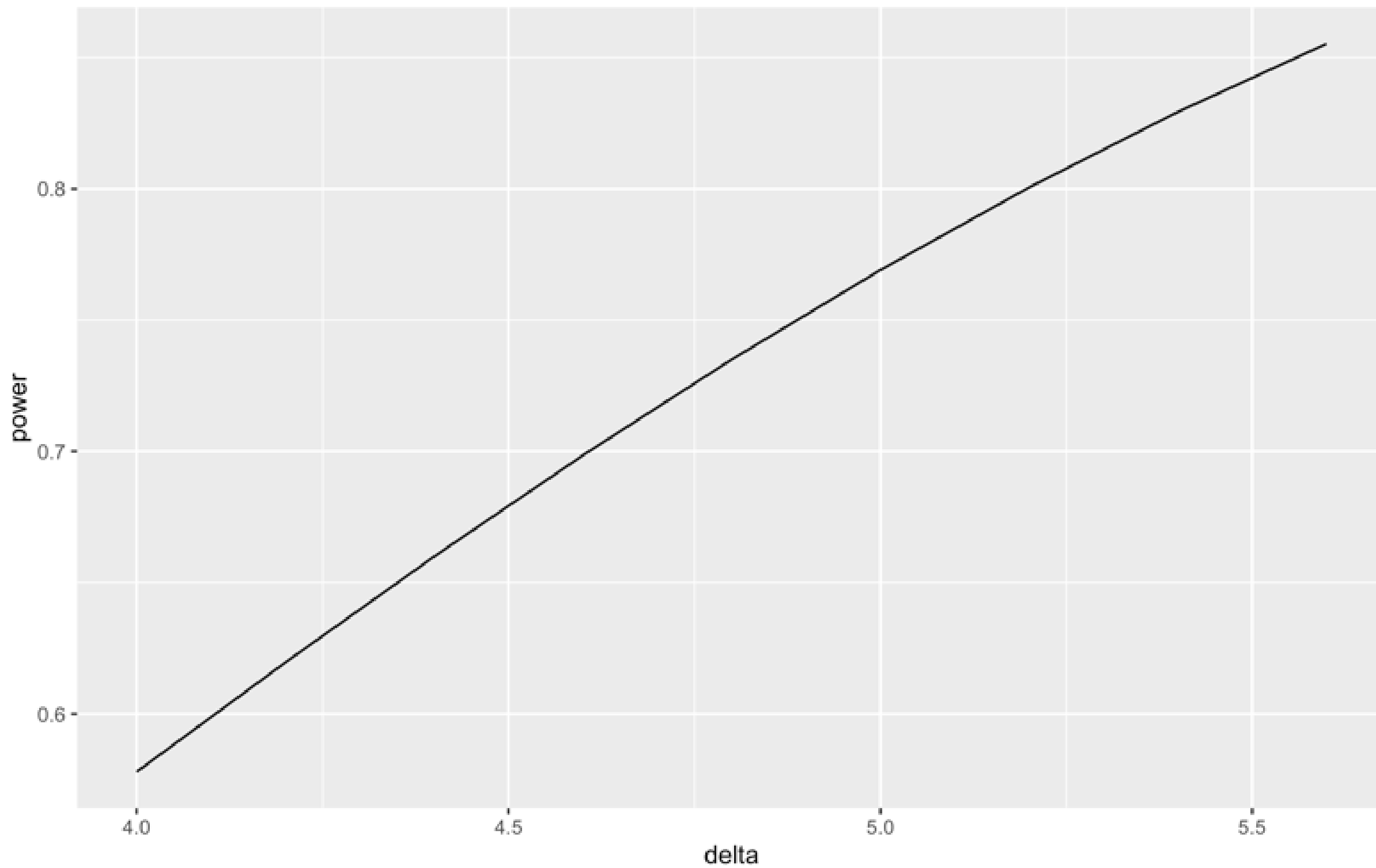

For the meta-analysis, a t-test for a difference of 4 points with 3*46 subjects in each group and a SD of 8.8 results in a power of .94; a t-test for a difference of 3 points results in a power of .81.

11 QUALITY CONTROL PLANS

Data quality will be assessed using REDCap validation methods and ongoing review of data (e.g., identification of missing data, outliers, etc.). Range and consistency checks will also be employed.

12 PROGRAMMING PLANS

Statistical software (SAS and R) will be used to produce results and raw data will be available for accompanying papers to be submitted for publication (following approval by Principal Investigators).

13 FUNDING AND ACKNOWLEDGMENTS

This research is supported by a grant from the National Institute of Mental Health (P50MH122351), which established the Center for Perioperative Mental Health at Washington University.

14 REFERENCES

1. Mangusan RF, Hooper V, Denslow SA, Travis L. Outcomes associated with postoperative delirium after cardiac surgery. *Am J Crit Care*. Mar 2015;24(2):156-63. doi:10.4037/ajcc2015137
2. Blumenthal JA, Lett HS, Babyak MA, et al. Depression as a risk factor for mortality after coronary artery bypass surgery. *Lancet*. Aug 23 2003;362(9384):604-9. doi:10.1016/S0140-6736(03)14190-6
3. Connerney I, Shapiro PA, McLaughlin JS, Bagiella E, Sloan RP. Relation between depression after coronary artery bypass surgery and 12-month outcome: a prospective study. *Lancet*. Nov 24 2001;358(9295):1766-71. doi:10.1016/S0140-6736(01)06803-9

4. McKenzie LH, Simpson J, Stewart M. A systematic review of pre-operative predictors of post-operative depression and anxiety in individuals who have undergone coronary artery bypass graft surgery. *Psychol Health Med*. Jan 2010;15(1):74-93. doi:10.1080/13548500903483486
5. Takagi H, Ando T, Umemoto T, Group A. Perioperative depression or anxiety and postoperative mortality in cardiac surgery: a systematic review and meta-analysis. *Heart Vessels*. Dec 2017;32(12):1458-1468. doi:10.1007/s00380-017-1022-3
6. Abraham J, Meng A, Siraco S, et al. A Qualitative Study of Perioperative Depression and Anxiety in Older Adults. *Am J Geriatr Psychiatry*. Mar 2 2020;doi:10.1016/j.jagp.2020.02.010
7. Kroenke K, Wu J, Yu Z, et al. Patient Health Questionnaire Anxiety and Depression Scale: Initial Validation in Three Clinical Trials. *Psychosom Med*. 2016 Jul-Aug 2016;78(6):716-27. doi:10.1097/PSY.0000000000000322
8. Freed B, Assall OP, Panagiotakis G, et al. Assessing blinding in trials of psychiatric disorders: a meta-analysis based on blinding index. *Psychiatry Res*. Oct 30 2014;219(2):241-7. doi:10.1016/j.psychres.2014.05.023
9. Levis B, Benedetti A, Thombs BD, Collaboration DESD. Accuracy of Patient Health Questionnaire-9 (PHQ-9) for screening to detect major depression: individual participant data meta-analysis. *BMJ*. Apr 9 2019;365:l1476. doi:10.1136/bmj.l1476
10. Mielenz TJ, Callahan LF, Edwards MC. Item response theory analysis of Centers for Disease Control and Prevention Health-Related Quality of Life (CDC HRQOL) items in adults with arthritis. *Health Qual Life Outcomes*. Mar 12 2016;14:43. doi:10.1186/s12955-016-0444-4
11. Gregory SH, King CR, Ben Abdallah A, Kronzer A, Wildes TS. Abnormal preoperative cognitive screening in aged surgical patients: a retrospective cohort analysis. *British journal of anaesthesia*. Jan 2021;126(1):230-237. doi:10.1016/j.bja.2020.08.026
12. Oughli HA, Chen G, Philip Miller J, et al. Cognitive Improvement in Older Adults in the Year After Hip Fracture: Implications for Brain Resilience in Advanced Aging. *Am J Geriatr Psychiatry*. 11 2018;26(11):1119-1127. doi:10.1016/j.jagp.2018.07.001
13. Arias F, Wiggins M, Urman RD, et al. Rapid In-Person Cognitive Screening in the Preoperative Setting: Test Considerations and Recommendations from the Society for Perioperative Assessment and Quality Improvement (SPAQI). *Perioper Care Oper Room Manag*. Jun 2020;19doi:10.1016/j.pcorm.2020.100089
14. Puspitasari AJ, Kanter JW, Busch AM, et al. A randomized controlled trial of an online, modular, active learning training program for behavioral activation for depression. *J Consult Clin Psychol*. Aug 2017;85(8):814-825. doi:10.1037/ccp0000223
15. Stein AT, Carl E, Cuijpers P, Karyotaki E, Smits JAJ. Looking beyond depression: a meta-analysis of the effect of behavioral activation on depression, anxiety, and activation. *Psychol Med*. Mar 6 2020:1-14. doi:10.1017/S0033291720000239
16. Choi NG, Pepin R, Marti CN, Stevens CJ, Bruce ML. Improving Social Connectedness for Homebound Older Adults: Randomized Controlled Trial of Tele-Delivered Behavioral Activation Versus Tele-Delivered Friendly Visits. *Am J Geriatr Psychiatry*. Mar 2 2020;doi:10.1016/j.jagp.2020.02.008
17. Egede LE, Dismuke CE, Walker RJ, Acierno R, Frueh BC. Cost-Effectiveness of Behavioral Activation for Depression in Older Adult Veterans: In-Person Care Versus Telehealth. *J Clin Psychiatry*. Aug 28 2018;79(5)doi:10.4088/JCP.17m11888
18. Farhang M, Miranda-Castillo C, Rubio M, Furtado G. Impact of mind-body interventions in older adults with mild cognitive impairment: a systematic review. *Int Psychogeriatr*. May 2019;31(5):643-666. doi:10.1017/S1041610218002302
19. Lenze EJ, Hickman S, Hershey T, et al. Mindfulness-based stress reduction for older adults with worry symptoms and co-occurring cognitive dysfunction. *Int J Geriatr Psychiatry*. Oct 2014;29(10):991-1000. doi:10.1002/gps.4086
20. Wildes TS, Mickle AM, Ben Abdallah A, et al. Effect of Electroencephalography-Guided Anesthetic Administration on Postoperative Delirium Among Older Adults Undergoing Major Surgery: The ENGAGES Randomized Clinical Trial. *JAMA : the journal of the American Medical Association*. Feb 5 2019;321(5):473-483. doi:10.1001/jama.2018.22005
21. Sinclair S, Hack TF, MacInnis CC, et al. Development and validation of a patient-reported measure of compassion in healthcare: the Sinclair Compassion Questionnaire (SCQ). *BMJ open*. 2021;11(6):e045988.
22. Kanter JW, Mulick PS, Busch AM, Berlin KS, Martell CR. The Behavioral Activation for Depression Scale (BADS): psychometric properties and factor structure. *Journal of Psychopathology and Behavioral Assessment*. 2007;29(3):191-202.

23. Alexopoulos GS, Raue PJ, Gunning F, et al. “Engage” therapy: behavioral activation and improvement of late-life major depression. *The American Journal of Geriatric Psychiatry*. 2016;24(4):320-326.
24. Fakes KL, Hall AE, Carey ML, et al. Development and validation of the MiPrep survey: an instrument assessing patients' perceived preparation for medical interventions including medical imaging, radiotherapy, and surgery. *Value in Health*. 2019;22(6):704-711.
25. Quality AfHRa. *About CAHPS*. 2020. https://www.ahrq.gov/cahps/about-cahps/index.html
26. Kroenke K, Wu J, Bair MJ, Kean J, Stump T, Monahan PO. The Patient Health Questionnaire Anxiety and Depression Scale (PHQ-ADS): Initial Validation in Three Clinical Trials. *Psychosom Med*. 2016;78(6):716-727.
27. Kroenke K, Krebs E, Wu J, et al. Stepped Care to Optimie Pairn Care Effectiveness (SCOPE) Trial: study design and sample characteristics. *Contemp Cln Trials*. 2013;34::270-281.
28. Kroenke K, Krebs EE, Wu J, Yu J. Telecare collaborative management of chronic pain in primary care: a randomized clinical trial. *JAMA*. 2014;312(3):240-248.
29. Blumenthal JA, Lett HS, Babyak MA, et al. Depression as a risk factor for mortality after coronary artery bypass surgery. *The Lancet*. 2003;362(9384):604-609.
30. Abraham J, Meng A, Siraco S, et al. A Qualitative Study of Perioperative Depression and Anxiety in Older Adults. *The American Journal of Geriatric Psychiatry*. 2020/03/02/ 2020;doi:https://doi.org/10.1016/j.jagp.2020.02.010
31. Kroenke K, Wu J, Yu Z, et al. The patient health questionnaire anxiety and depression scale (PHQ-ADS): Initial validation in three clinical trials. *Psychosomatic medicine*. 2016;78(6):716.
32. Mielenz TJ, Callahan LF, Edwards MC. Item response theory analysis of Centers for Disease Control and Prevention Health-Related Quality of Life (CDC HRQOL) items in adults with arthritis. *Health Qual Life Outcomes*. 2016;14(1):43.
33. Inouye SK, Leo-Summers L, Zhang Y, Bogardus Jr ST, Leslie DL, Agostini JV. A chart-based method for identification of delirium: validation compared with interviewer ratings using the confusion assessment method. *Journal of the American Geriatrics Society*. 2005;53(2):312-318.
34. Kronzer VL, Jerry MR, Abdallah AB, et al. Preoperative falls predict postoperative falls, functional decline, and surgical complications. *EBioMedicine*. 2016;12:302-308.
35. Cleeland C, Ryan K. Pain assessment: global use of the Brief Pain Inventory. *Annals of the Academy of Medicine, Singapore*. 1994;23(2):129-138.
36. Glasgow RE, Vogt TM, Boles SM. Evaluating the public health impact of health promotion interventions: the RE-AIM framework. *American journal of public health*. 1999;89(9):1322-1327.
37. Weiner BJ, Lewis CC, Stanick C, et al. Psychometric assessment of three newly developed implementation outcome measures. *Implement Sci*. 2017;12:108. doi:10.1186/s13012-017-0635-3
38. Crofton C, Darby C, Farquhar M, Clancy C. The CAHPS® hospital survey: development, testing, and use. *Joint Commission Journal on Quality and Patient Safety*. 2005;31(11):655-659.
39. Harris PA, Taylor R, Minor BL, et al. The REDCap consortium: building an international community of software platform partners. *Journal of biomedical informatics*. 2019;95:103208.
40. Harris PA, Taylor R, Thielke R, Payne J, Gonzalez N, Conde JG. A metadata-driven methodology and workflow process for providing translational research informatics support. *J Biomed Inform*. 2009;42(2):377-81.
41. Freed B, Assall OP, Panagiotakis G, et al. Assessing blinding in trials of psychiatric disorders: a meta-analysis based on blinding index. *Psychiatry research*. 2014;219(2):241-247.
42. Riley RD, Lambert PC, Abo-Zaid G. Meta-analysis of individual participant data: rationale, conduct, and reporting. *BMJ*. 2010;340:c221. doi:10.1136/bmj.c221
43. Sinclair S, Hack T, MacInnis C, et al. Development and validation of a patient-reported measure of compassion in healthcare: the Sinclair Compassion Questionnaire (SCQ). *BMJ Open*. 2021;11(6):e045988.
44. Kroenke K, Wu J, Yu Z, et al. Patient health questionnaire anxiety and depression scale: initial validation in three clinical trials. *Psychosom Med*. 2016;78(6):716-727.
45. Kroenke K, Krebs E, Wu J, et al. Stepped Care to Optimize Pain care Effectiveness (SCOPE) trial study design and sample characteristics. *Contemporary clinical trials*. 2013;34(2):270-281.
46. Kroenke K, Krebs EE, Wu J, Yu Z, Chumbler NR, Bair MJ. Telecare collaborative management of chronic pain in primary care: a randomized clinical trial. *JAMA*. 2014;312(3):240-248.
