# Supplementary material for: Perioperative Depression and Anxiety Care in Older Patients: A Randomized Clinical Trial
Source: JAMA Netw Open. 2026 Sep 9;9(9):e2632619. doi: 10.1001/jamanetworkopen.2026.32619 (PMC13559599; doi:10.1001/jamanetworkopen.2026.32619)
Supplement: Supplementary file 2 [file jamanetwopen-e2632619-s002.pdf]

Supplemental Online Content

Abraham J, Holzer KJ, Haroutounian S, et al. Perioperative depression and anxiety in older patients: a randomized clinical trial. *JAMA Netw Open*. 2026;9(9):e2632619. doi:10.1001/jamanetworkopen.2026.32619

eFigure 1. Perioperative mental health intervention
eMethods 1. Perioperative mental health intervention manual of procedures (MOP) and interventionist training
eTable 1. Intervention delivery process measures
eTable 2. Cronbach alphas at baseline and 3 months for each surgical sub-population for depression (PHQ-9) and anxiety (GAD-7) scales
eTable 3. Impact of intervention on health-related quality of life
eTable 4. Incidences of delirium and all-cause readmissions
eTable 5. Impact of intervention on persistent postsurgical pain
eTable 6. Intervention impact on falls
eTable 7. Impact of intervention on length of hospital stay and length of ICU stay
eFigure 2. Intervention reach
eTable 8. Psychological management reach details
eTable 9. Pharmacoligical optimization reach details
eFigure 3. Acceptability, appropriateness, and feasibility of psychological management
eFigure 4. Acceptability, appropriateness, and feasibility of pharmacological optimization
eTable 10. Patient satisfaction with psychological management
eTable 11. Patient satisfaction with pharmacological optimization
eTable 12. Intervention implementation fidelity to psychological management and pharmacological optimization
eTable 13. Summary of planned and completed psychological management activities
eTable 14. Summary of pharmacological interventions
eTable 15. Medication types within pharmacological optimization sessions
eTable 16. Intervention delivery for intervention patients
eTable 17. Patient and interventionist experiences with the intervention
eTable 18. Target engagement survey scores
eMethods 2. Perioperative mental health intervention implementation evaluation methods: data collection and analysis
eReferences

This supplemental material has been provided by the authors to give readers additional information about their work.

© 2026 Abraham J et al. *JAMA Network Open*.

Abbreviations

PMH	Perioperative Mental Health
BA	Behavioral Activation
MO	Medication Optimization
RCT	Randomized Clinical Trial
EUC	Enhanced Usual Care
LSM	Least Squares Mean
SD	Standard Deviation
SE	Standard Error
OR	Odds Ratio
PHQ-ADS	Patient Health Questionnaire Anxiety and Depression Scale
SCQ-SF	Sinclair Compassion Questionnaire-Short Form
BADS	Behavioral Activation Depression Scale
PIM	Potentially Inappropriate Medication

© 2026 Abraham J et al. *JAMA Network Open*.

eFigure 1. Perioperative Mental Health Intervention.

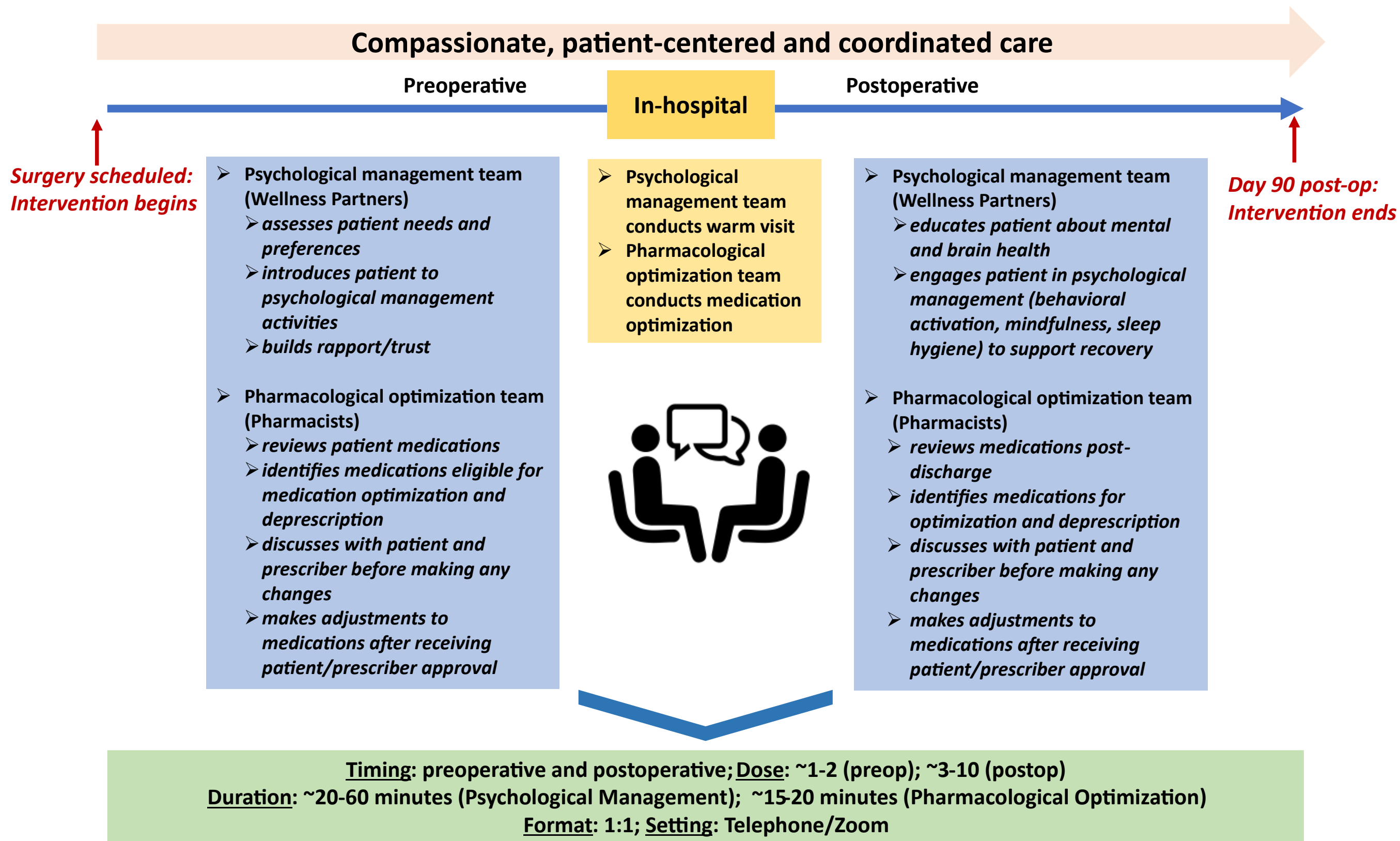

© 2026 Abraham J et al. *JAMA Network Open*.

eMethods 1. Psychological management intervention manual of procedures (MOP) and interventionist training. (*Note: pharmacological management is referred to as medication optimization in the manual of procedures and patient facing materials*.)

Center for Perioperative Mental Health
Wellness Program Manual

1. Overview

The Center for Perioperative Mental Health Wellness Program integrates two main components, medication optimization and a psychosocial component using a supportive approach and behavioral activation principles and techniques, to prepare patients for surgery and to promote an enhanced recovery process.

Medication optimization aims to a) reduce the use of problematic medications that increase risk for falls and delirium in older adults and b) increase sub-therapeutic doses of antidepressant medications to therapeutic levels. Medication optimization during the perioperative period also entails monitoring medications throughout the hospital stay to ensure harmful medications are not initiated and that patients are quickly re-started on any antidepressant medications that may have been held during surgery. Medication adjustments are made using a collaborative approach to decision-making and with the interest of helping the patient to feel and think their best throughout the surgical period. Full details of medication optimization can be found in the (separate) Medication Optimization SOP.

Behavioral activation is an evidence-based treatment for depression that aims to engage individuals in activities that are personally rewarding. We have adapted behavioral activation for older adult surgical patients who are experiencing symptoms of anxiety and/or depression prior to surgery. Behavioral activation techniques are used to support the patient in an individualized, active recovery from surgery, which encourages patients to gradually re-engage in the important activities in their life and cope with the stressors of the surgical period.

Behavioral activation will begin prior to or soon after the surgery, with the initial sessions focused on engagement and assessing the patient's needs and concerns. Patients who present with prominent worry and anxiety (indicated by responses on PHQ-ADS or in discussion) will be invited to work on activity plans for self-directed mindfulness practice. Patients with significant sleep disruption will be provided with resources on sleep hygiene and encouraged to develop activity plans for improving sleep routines. Patients with concerns about their cognition will be provided with resources on evidence-based cognitive training programs and supported in engaging in activity plans for cognitive training exercises. Patients may also choose other kinds of enjoyable and/or productive activities as well.

The Wellness Program is designed to be flexible and patient-centered and can be carried out remotely via phone or secure web conference, with optional in-person visit/s while the patient is hospitalized. It is designed for patients undergoing major cardiac, oncologic, and orthopedic surgeries. The Wellness Program is designed to be delivered by a masters-level social worker or counselor, referred to as a Wellness Partner. The Wellness Program begins soon after the patient's surgery is scheduled and continues for approximately 3 months after the surgery. Sessions typically occur on a weekly basis initially and then reduce to every two weeks. Frequency of sessions can be adjusted according to the patient's needs and preferences. Session duration is approximately 40 minutes per session but can be adjusted between 20 and 60 minutes depending on patient needs and treatment goals.

The style of the Wellness Program is warm, supportive, and compassionate. The program is semi-structured, providing opportunity for individualizing therapy to meet the patient's changing needs before and after surgery.

2. Introductory Call

Upon receiving the referral, contact the patient within 1-3 days to introduce yourself and arrange their first session.

- Introduce yourself, and the Wellness Program generally. This can be brief since you'll go into more detail in Session 1, but answer any questions that the patient has.
- Verify you have their correct contact information and mailing address.
- Discuss their preferred contact method and time of day for calls generally.
- Would they like any reminders or confirmations about scheduled sessions?
- Do they prefer phone or zoom sessions?
- Do they expect that they would like a visit when they are in hospital?
- Let them know when they can expect to receive their materials in the mail.

- ➢ Arrange date, time, and modality for their first session, accommodating pharmacy team member availability.
- Ask the patient to have a complete list of their medications (or all of their pill bottles) available to review with the pharmacy team member for their first session.

> *CASSANDRA: Hi Jim, my name is Cassandra Williams. I'm calling with the Center for Perioperative Mental Health at Washington University. I'm looking forward to working with you in the Wellness Program.*
> *Today I just wanted to introduce myself, confirm a few things with you, and set a time for your first session. Before we do that, what questions do you have for me about the program?*

3. Session 1 *Assess and Engage*

Session 1 has 3 objectives: (a) to orient the patient to the Wellness Program and discuss expectations for working together, (b) to assess the patient's need for medication optimization and develop a personalized medication optimization plan in conjunction with the pharmacy team member (described briefly below and in Medication Optimization SOP), and (c) to demonstrate interest in the patient as a person and begin to understand what is important (what matters most) to the patient.

1. Preparing for the Session

- ➢ Review the information collected at screening and baseline:
  - o PHQ-ADS (see appendix):
    - ▪ Depression Symptom Severity (PHQ-9)
    - ▪ Anxiety Symptom Severity (GAD-7)
- ➢ Arrange for pharmacy team member to be available to join the call to discuss medications. If a pharmacy team member is not available, the wellness partner can perform this task in collaboration with pharmacy team. Refer to Medication Optimization SOP.

2. Opening:

- ➢ Review agenda and seek patient's input.
- Ask the patient to retrieve a complete list of their medications (or all of their pill bottles).

3. Orienting to the Wellness Program and Clarifying Expectations

Wellness Partner: Provide a brief and interactive introduction to the Wellness Program. Key points to address:

- ➢ This is a program to support mental and physical health (overall wellness) around the time of surgery. This can be a time when people aren't feeling their best (mentally or physically).
- ➢ One way that we do this is by having regular sessions to provide support as you prepare for and recover from surgery.
  - o We will focus on things you can do to feel the best you can.
- ➢ My role is to be your Wellness Partner: to partner with you and guide you to promote your well-being. My background is in (social work / counseling) and working on research studies.
- ➢ The other way we work to support your mental health is by taking a close look at any medications that can affect your brain.
- ➢ Turn things over to the pharmacy team member to continue.

4. *Introduction to Medication Optimization*

Pharmacy Team Member: Refer to Medication Optimization SOP

5. Getting to Know the Patient

Wellness Partner: Ask open-ended questions and express interest in the patient and their story. Assess the following areas. Sample language is provided in italics.

1. **Health and the current surgery:** Ask about the upcoming surgery as well as their related and other health concerns, from their perspective. How have their health conditions affected their life and functioning?
   Tell me about the surgery you have coming up.

How has your health been otherwise?
How has that affected your day-to-day life and functioning?

2. **Mental health experiences:** Summarize the depression and anxiety symptoms the patient reported at baseline, making sure to address sleep and stress specifically. Ask which symptoms are the most bothersome. Find out how long the patient has been experiencing them and how they have impacted their life. Ask about their coping strategies.
I see you've been experiencing some days with a low mood, low energy, trouble sleeping, and trouble concentrating. Which one of those problems has been bothering you the most?
Would you say you've been feeling stressed, or like it's hard to relax?
Tell me about the trouble you've been having with your sleep.
How long has that been going on?
How has that impacted your life?
What have you been doing to cope with that?

3. **Current symptoms of depression and anxiety:** Assess with brief symptom assessment:
These questions are intended to assist in monitoring progress. If the exact wording or response options don't resonate with the patient, different questions can be used.

4. **Activation and activities:** Ask the patient about how they typically spend their time. Ask whether that is ideal for them or if there are things they would like to be doing but have stopped or are unable to. Get a sense for whether they are high or low in activation.
Tell me about how you usually spend your time? What's a typical day like for you?
Is that ideal for you, or are there some things you have stopped doing?
Has your medical condition (and/or treatments) interfered with your usual activities?
Would you say you're the type of person who likes to keep pretty busy? Or do you find yourself with a lot of down time?

5. **Cognitive concerns:** Ask whether the patient has any concerns about their thinking abilities or memory. Ask about mentally stimulating activities that the patient does.
Have you been worried about your mind isn't as sharp as you'd like it to be? Are you worried about your memory getting worse?
Do you like to do things that challenge your brain, make you think, or require a lot of focus?

6. **Hopes and aspirations:** Ask what the patient hopes for in their recovery from surgery, and what they hope to get out of the Wellness Program.
Tell me in your words – what would be a good outcome from you, from the surgery? What are you hoping to be able to do after you recover?
What helped you decide to participate in this program? What are you hoping to get out of our work together? How can I best support you during this time?

Other areas to explore if conversation is limited:

7. **Concerns and fears:**
Do you have any concerns or fears as you approach this surgery?

8. **Social supports and stressors:**
Tell me about the people in your life. Do you feel like you have enough support?
Do you have anything especially stressful or difficult going on in your life? Either something new or something that's been going on a long time?

9. **Other positive and negative experiences in their life:**
What are the things in your life that are going well?
What are the things in your life that are not going so well?

Make the connection: Summarize and validate the patient's responses and convey how you think the Wellness Program could help them with their concerns.

© 2026 Abraham J et al. *JAMA Network Open*.

6. Next Session:

Typically, Session 2 would occur in approximately one week, but you may schedule it sooner in order for it to take place before their surgery.

Decide on the format (phone / zoom), date, and time for the next session.

Let the patient know when they can expect to receive the Wellness Program materials in the mail. It can be helpful for the patient to review and fill out the "What are Your Wellness Goals?" form for discussion at the next session.

Health and the current surgery

CASSANDRA: Tell me about the surgery you have coming up.

JIM: I'm having a triple bypass and an artery replaced in three weeks.

CASSANDRA: I see. How are you feeling going into it?

JIM: Well, I can't say I'm excited about it. More like terrified. But I know that I need it and I hope afterwards that it helps me to feel better.

CASSANDRA: That makes sense that you're feeling hopeful, and also worried at the same time. You say you'd like to feel better – how have you been feeling lately?

JIM: The biggest problem is I really don't have much energy to do things; and anything that I do, I get fatigued and winded really easily. Things I'd never give a second thought to before, like tying my shoes gets me short of breath. Then there's the things I'm afraid to do because if I get an episode of chest pain, it's really scary and uncomfortable.

CASSANDRA: I see, that does sound scary. It sounds like your heart condition has had a big impact on your daily life. Can you tell me more about how things have changed for you since you've been dealing with this?

JIM: The most significant change is that I've been on medical leave from work for the past month while we've been sorting all of this out. My job can get physical. I'd noticed things were harder than they used to be, and after work was over I wasn't good for much of anything, I just had to rest. Now being on leave, I feel pretty useless. I had planned on being able to work another 5 years at least so I worry a lot about what happens if I can't get back to work.

Mental health experiences

CASSANDRA: I can see how being away from work could cause worry and feeling badly about yourself. That's actually what I wanted to ask you about next. I noticed on the surveys we did with you on Tuesday that you'd been feeling irritable, worrying a lot, having trouble relaxing, trouble concentrating, and some trouble with your sleep. Did I miss anything important?

JIM: No, that about sums it up.

CASSANDRA: How long has that been going on?

JIM: Well truthfully, I have always been a worrier and when I'm feeling stressed, I tend to have a short fuse. So those are not new experiences for me. But usually I can set aside my worries and relax, or pay attention when I need to. It's only been lately those things have been more of a struggle. The trouble with my sleep is new too.

CASSANDRA: OK, I hear you. So what has been going on with your sleep?

JIM: I don't know if it's because I'm not as active during the day, but I have the hardest time falling asleep at night. It's gotten to where I don't even think about going to bed until nearly midnight because I'd rather watch the late show than lie awake for an hour or two. These meds I started on have me getting up to go to the bathroom once or twice a night. Then it's a coin toss if I'm able to get back to sleep right away, or I might be lying awake again for an hour or so.

CASSANDRA: That sounds exhausting! So between the sleep, irritability, worrying, trouble focusing, and trouble relaxing, which has been bothering you the most?

JIM: I think if I could get better sleep, maybe some other things would improve. But the most worrying to me is having trouble focusing. I just really don't feel like myself and I worry I'll make mistakes.

Activation and activities

CASSANDRA: Getting back to what you said earlier – it sounds like normally you would be working. Do you work full time? How would you normally spend your free time?

JIM: I work full time but I do four ten hour shifts. I usually work Tuesday through Friday. I like to spend most of my time off outside if I can. Our property is out in the country so it's real easy to get out and fish, hunt, hike, go four-wheeling…whatever you feel like. Up until this season I did all of our mowing and yard work myself. There's always something to do out here, whether it's for fun or something that has to get done.

Cognitive concerns

CASSANDRA: I know you mentioned having some trouble focusing. Have you been worried about your memory, or feeling like you're not as sharp as you used to be?
JIM: That's true, I definitely don't feel as sharp as I used to. It's like my brain is just as tired as my body is. I do worry about what happens if I go back to work and I still feel this fuzzy.
CASSANDRA: A lot of people have that same concern. What kinds of things do you do that challenge your brain – in other words, what gives your mind a workout?
JIM: My job in the machine shop requires a lot of focus, quick thinking, and problem solving.

Hopes and aspirations

CASSANDRA: Shifting gears a little, I want to talk about your hopes for the future. What would be a good outcome from you, from the surgery? What are you hoping to be able to do after you recover?
JIM: Well, I need to be able to get back to work for at least a few more years, practically speaking. I really just want to get back to living life – getting out and enjoying the outdoors with my family and friends.
CASSANDRA: I'd like to see that for you too. It sounds like you guys have a good time together. What were you hoping for when you signed up for this program?
JIM: If I'm being honest, I really just thought it'd be nice having someone to talk to about this. I know my family is worried about me so I try to put on a brave face for the most part.

4. Session 2 *Develop Personalized Wellness Plan*

The main objective of Session 2 is to develop a shared understanding of the Wellness Program and how it can be helpful for the patient during the surgical period. The Wellness Partner should follow up on any medication optimization recommendations for the patient. Following up on medication optimization recommendations can typically occur briefly at the start of the call. It may be helpful for the pharmacy team member to join the first few minutes. Refer to Medication Optimization SOP. The Wellness Partner should learn about the patient's daily activities. If there are areas from Session 1 that were not assessed fully, they should be revisited. Use the "What are Your Wellness Goals?" handout to guide discussion about helpful activities to support recovery. If the patient's goals become well-focused, consider setting a quick wellness activity goal.

1. Preparing for the Session

- Review the information gathered about the patient during Session 1.
- Review the medication optimization recommendations.

2. Opening:

Invite patient to retrieve the "What are Your Wellness Goals?" handout from their materials.

- Review agenda and seek patient's input (in other words, ask whether there is anything else they'd like to make sure to discuss).
- Check in on the patient's health and recovery.
- Brief symptom assessment.
- If pharmacy team member is not attending the session, ask patient if they have had any medication changes, including OTC. Relay any changes to assigned pharmacy team member as needed.

3. Medication Optimization: Follow-Up

Refer to Medication Optimization SOP

4. Developing a Shared Understanding and Conveying the Approach

1. Summarize and review the information the patient described during Session 1, including both negative circumstances as well as strengths and aspirations.
2. Assess current symptoms with brief symptom assessment.
3. Ask if they have noticed any changes in what they're doing or how they're feeling in anticipation of the surgery or as a result of the surgery. Are there things they are doing more or less of? Have those changes caused any problems?

4. Validate the patient's experiences. Having surgery is a major stressor and can lead to all sorts of different emotions and reactions. It is completely normal to experience a wide range of emotions.
5. Learn about the patient's current and ideal daily routines, activities, and preferences. Discuss how day-to-day activities impact their mood.
6. Discuss how the Wellness Program may be helpful to them:
   a. *The Wellness Program can help you take an active role in your own recovery by planning activities that help you feel better (instead of just waiting or hoping to feel better). We would work towards a gradual, purposeful re-engagement with your normal life and activities*.
   b. Introduce targeted new activities based on the patient's needs, where the surgical period presents an opportunity to try new activities to feel happier and healthier:
      i. [if worry, stress, and/or anxiety are prominent]: *Mindfulness has been shown to help people cope with worrying and stress.*
      ii. [if patient has significant sleep disruption]: *We can discuss those resources you received with evidence-based strategies for improving your sleep. We can work together on trying them out.*
      iii. [cognitive concerns]: *We can discuss those resources you received about challenging your brain so you can maintain or improve your memory and thinking skills.*
      iv. Decrease in enjoyable and/or productive activities: We can focus on other activities that you enjoy, that give you a feeling of accomplishment or satisfaction, or that help you overcome some challenges that you may be facing.
7. Invite patient to consider what they may want to work towards together. Use this to transition to discussing the "What are Your Wellness Goals" handout.

5. What are Your Wellness Goals?

Introduce this page as a way to explore activities to support their recovery. It's easiest if the patient has the form in front of them during the session, but if they don't have the materials (or have limited vision or literacy), the Wellness Partner can verbally present the material.

- ➢ Ask about each activity, including whether it is something that they already do and would like to continue doing or do more often after surgery, something they'd like to start doing for the first time or start doing again, or if it is something they don't want to do during their recovery.
- ➢ Ask about the relevant details, context, and significance for activities that they want to continue, start, or restart. Identify and discuss anything that the patient is curious about or doesn't understand.
- ➢ Assess for other important activities that the patient may want to do or work towards during their recovery.
- ➢ Based on what you've learned and discussed, identify one or two activities to begin with to focus on together.
  If the patient is eager to get started, they can set a quick wellness activity goal to complete before the next session. This quick goal setting does not need to go to the level of detail that will occur each session beginning in Session 3. This can be a brief discussion just to get started. Depending on the goal, you may invite them to keep track of the outcome informally.

6. Next Session:

Typically, Session 3 would occur in approximately one week.
If the patient set a quick activity goal, provide a reminder and encouragement.

Developing a shared understanding and conveying the approach

CASSANDRA: Thanks for taking time for our session today. I really appreciated what you shared with me last week. Today I'd like to discuss in more detail what your goals are and how we can work together on them. Before we do, is there anything else you'd like to be sure we discuss today?
JIM: That sounds fine.
CASSANDRA: Ok, great. Last time we talked, you told me about how it's been challenging for you lately while you've been on leave from work. It sounded like it's been hard for you to do a lot of the things that you enjoy and that make you feel worthwhile. Some of the problems that stood out to me were the trouble you've had with your sleep, feeling fuzzy, like it's hard to concentrate, and feeling tense or irritable. At the same time, I heard a lot of strengths – you have a job that challenges you and leaves you with time off to enjoy your hobbies. And it sounds like you have lots

© 2026 Abraham J et al. *JAMA Network Open*.

of people in your life who enjoy spending time with you. I know we talked for a while, but does that all ring true to you?
JIM: Yep, you pretty much got to the heart of it.
CASSANDRA: Good. Well, while we start thinking more about our work together, I want to ask you a little more about how things have been for you during this time leading up to your surgery. Have you noticed any other changes in how you're feeling, or what you're doing? Is there anything you're doing more of or less of than usual?
JIM: Well, like we talked about before, I find it's hard to relax. I always feel like I should be doing something, but I have all of these days where there's really nothing to do. At least, nothing that I can do anyway. My wife says I should just rest and try to enjoy the time off but I feel guilty being home all day when she goes off to work. Then she asks me about my day and I honestly have no idea where the time went.
CASSANDRA: I can definitely understand losing track of time when you don't have as much going on. How have you been spending the days lately? I guess it's been pretty different than your norm.
JIM: Well, a big difference is I sleep in until around 8:30. I normally would wake up at 6. But seeing as I don't get to sleep until 12 or 1 in the morning, I feel like I may as well. I'll usually make myself some coffee and toast and take it out on the patio while the dogs come out with me. Then I'll head in to take a shower and get dressed. After that I'll just watch TV or read news articles on my tablet. Maybe do some laundry or something to try to help out. I'll sit out with the dogs again at lunch time and then come back in and watch some more TV or maybe doze off for a few minutes. Then I'll go and pick up my two youngest grandkids from school at 3. That's been fun. They usually have their grandparents on the other side to do that but I asked if they wanted a few weeks off while I'm home from work. Then when I get home around 5:30or 6, I'm pretty beat and my wife and I just eat dinner, watch some TV, and go to bed.
CASSANDRA: Thanks for walking me through that. I'd like to understand a little more how you're feeling as you're doing each of those things. You mentioned being with your grandkids after school had been fun – what's your mood been like then? Have you had any of that worry or irritability?
JIM: You know, that's an interesting question. You might expect being around kids to be kind of irritating at least some of the time. They're at a really good age though, they're in 1st grade and 3rd grade and they're always on good behavior for me. That's been the best part of the day.
CASSANDRA: That's so nice you're able to enjoy spending that time together. What about when you're sitting out with your dogs? How are you feeling then?
JIM: Well, at first it's nice to get outside and see them out their enjoying themselves. But that's where I do I do start to get sort of restless. I'll look over at the garage and think of all the things in there that need fixing. I'll notice all the hedges getting overgrown and the potted plants that will need to come inside soon. You know, stuff like that. I mean honestly, I could do some of it but I fatigue so quick it would take me forever. And I really do get afraid of having more episodes of chest pain. I just worry something would happen and they wouldn't be able to help me fast enough because I'm here all alone during the day. So I just sit.
CASSANDRA: I can hear how much it bothers you not being up for those chores and that fear of being alone and needing help. It's very normal to have those kinds of feelings. Let me ask you about the other big part of your day. How are you feeling when you're watching TV and reading news articles on your tablet?
JIM: Honestly, pretty bored and zoned out. That's when I feel the most fuzzy and unfocused. Or I'll get annoyed with what I'm watching or reading.
CASSANDRA: It seems like those activities are more just to pass time than things you really enjoy?
JIM: Yeah, you could say that. I mean normally, it would just be an hour or two before bed and that I would look forward to after a long day. But I'm not used to having all this time on my hands.
CASSANDRA: That makes sense, this is a big change for you! I have a lot of hope for you that we'll be able to work together to find more meaningful ways for you to spend this time, and that you can have a peaceful recovery before transitioning back to your normal life. We'll have to talk a little more about what to prioritize first, but if sleep is the biggest concern for you that's definitely something we could work on improving. It sounds like being bored is a problem for you: we could work on finding ways for you to be more engaged in interesting things, even though

© 2026 Abraham J et al. *JAMA Network Open*.

you're not feeling your best. I also wanted to mention to you that one approach people have found helpful when dealing with stress and worry is Mindfulness. This is where you work to become more aware in the present moment, without having judgements about what you're experiencing. If any of those approaches sound helpful to you, there's information about them in the materials that we mailed to you. Did you receive those yet?
JIM: Yeah, I got them a few days ago but I didn't look over them yet.

What are Your Wellness Goals?

CASSANDRA: Great, I'm glad they arrived. If you have a minute to go get them, there's one thing I was hoping we could look at together. Could you turn to the page that says "What Are Your

	Continue doing or do more often	Start doing or start doing again	Don't want to do
Spending time with my spouse, friends, or family.	X		
Mindfulness, meditation, or relaxation techniques.			X
Religious or spiritual practices.			X
Maintaining my thinking and memory skills.		X	
Challenging my brain with brain training exercises.		X	
Improving the amount or quality of my sleep.		X	
Pursuing a hobby.		X	
Going out in the community for errands or socializing.			X
Doing paid or volunteer work.			
Taking care of the home or yard.		X	
Exercising or working out.			X
Cooking at home.			X
Healthy Eating.			X

Wellness Goals" at the top?
JIM: Ok, sure. I got it right here.
Jim and Cassandra discuss the What are Your Wellness Goals handout. This is how Jim's looks when they're finished.

CASSANDRA: This is great, it really helps to see what's important to you. Out of those activities that you want to start doing or start doing again – are there one or two that you'd really like to focus on now?
JIM: I think for now, what would help me the most is doing something to get my brain more active. I do think it's turning into applesauce with all this TV. I used to really like reading historical fiction but I just kind of got away from it.
With my hobbies, some of them I really wouldn't feel comfortable doing right now. But I realized there really is nothing keeping me from going fishing a few times before the surgery.
CASSANDRA: I'm so glad you're getting some ideas from this. Would you like to set a goal to complete one of those activities before we meet again next week?
JIM: You know what, I think I'll plan to go fishing over the weekend.

Session 3 *Planning Your Wellness Activities*
This session begins with assessing current symptoms and checking in on the patient's health and recovery. If the patient set a quick wellness activity goal in the previous session, discuss how it went. Following the previous session's discussion about the patient's priorities and wellness goals, the primary aim of this session is to further define the patient's wellness goals and, concretely plan at least one wellness activity for the patient to complete before the next session. Include a discussion of how to record progress. Use "Planning Your Wellness Activities" as a guide.

© 2026 Abraham J et al. *JAMA Network Open*.

7. Opening:

Invite patient to retrieve the "Planning Your Wellness Activities" handout from their materials.

- Review agenda and seek patient's input.
- Check in on the patient's health and recovery.
- Brief symptom assessment.
- If pharmacy team member is not attending the session, ask patient if they have had any medication changes, including OTC. Relay any changes to assigned pharmacy team member as needed.

8. Reviewing the Wellness activity goal

If the patient set a quick wellness activity goal during Session 2, discuss how it went.

- What progress did they make in achieving their goal?
- What challenges or barriers did they encounter?
- How did they feel before / during / after?

Take these experiences into account during the discussion about planning wellness activities.

9. Planning Your Wellness Activities

This is a collaborative process between the patient and Wellness Partner. Planning wellness activities should be clear, concrete, and focused on something the patient *does*. Planning activities should consider the difficulty of the task for the patient in their current circumstances. The "Planning Your Wellness Activities" handout should be used as a guide for discussion and for the patient to record their goals.

1. Introduce the concept of planning in the Wellness Program:
    a. "I'd also like to work with you each week to make a plan for doing something important to you to help you feel your best. You can make a different plan each week, or continue towards the same goal for several weeks in a row."
2. Collaboratively identify activities to work on, using the "What are Your Wellness Goals" activities as a starting point. Provide additional encouragement if the patient could benefit from mindfulness, cognitive training, or sleep hygiene activities.
3. Assess the difficulty of the activity in the current context. Break tasks into smaller parts if needed.
4. Develop a concrete plan for engaging in at least one activity. Ideally this plan should identify what the patient is going to do, where, when, and with whom they are going to do it. It is fine to keep the plan more flexible for patients who prefer it. When you follow-up, you can assess whether it would be more helpful to change the level of specificity / flexibility.
    a. The patient may use multiple copies of the "Planning Your Wellness Activities" handout if they would like to plan multiple activities. This can be helpful in later sessions once they have had some success that they want to build on.
5. Ask whether anything could get in the way or make it difficult for them to enact their plan. Brainstorm solutions to these obstacles. Ask whether anything could help make it easier for them to achieve their goal.
6. Discuss how to record progress towards the goal, using the space provided on the "Planning Your Wellness Activities" handout or another method preferred by the patient. Ideally, gain commitment from the patient to record their progress towards their goal for the next session.
    a. Optionally, some patients may like to record using the "Wellness Journal" handout.

1. Sample wellness activity plans for optional components:

Reviewing the handouts is a great first step. This can be done in a session, or the patient can review on their own.

- Mindfulness:
    - Start each day with a mindful check in before having coffee.
    - Download Healthy Minds app and use it 3 times a week after dinner.
    - Order MBSR Every Day and read one practice each day before bed.
- Improving Sleep:
    - Update sleep diary every morning for two weeks.
    - Sit out on porch for 30 minutes every morning.
    - Set a 'get ready for bed' alarm on phone for 10pm every night.
- Brain training / mental stimulation:
    - Use Happy Neuron for 30 minutes on Monday, Wednesday, and Friday.

© 2026 Abraham J et al. *JAMA Network Open*.

- Try a different Happy Neuron game each weekday.
- Call friend to discuss current events after reading the Sunday paper.

10. Next Session:

Typically, Session 4 would occur in approximately one week.
Encourage the patient in their goal/s and keeping track of the outcome.

Reviewing the Wellness Activity Goal

CASSANDRA: At our last session, you set a goal to go fishing over the weekend. Tell me how that went for you.
JIM: Well, I mentioned it to my wife and she wanted to go along. She invited our two older grandkids. So we had a pretty fun day of it and gave my son and his wife a day to spend together too.
CASSANDRA: I'm sure they enjoyed that! How was it for you?
JIM: I really enjoyed it. I was glad I got to spend time with them before I go into this surgery.
CASSANDRA: I'm so glad you made that happen. Did you run into any challenges along the way?
JIM: You know, as soon as we got off the phone, I realized that I didn't really want to be out there on the lake all by myself. And I wanted an extra hand loading the boat back up on the trailer. I just kind of mentioned it to my wife, that I was worried about going by myself, and she said she'd been missing doing those kinds of things with me too. I didn't realize. So I guess my challenge was thinking I had to do it by myself.
CASSANDRA: That's a great lesson to learn – that you don't have to do everything yourself!

Planning Your Wellness Activities

CASSANDRA: Your goal to go fishing was a great way to get started in the Wellness Program. I'll be working with you at each session to make a plan for you to do something that helps you feel your best. You can make a different plan each week, or continue towards the same goal for several weeks in a row. How does that sound to you?
JIM: That should be fine.
CASSANDRA: Great! I think the first thing is to decide what's most important to you right now, and we can go from there. Last week when we talked, your two main priorities were to spend more time on your hobbies and to get your brain more active.
JIM: Yeah that's right. I think what I could really use is a hobby I can do even when I'm at home recovering and can't go anywhere.
CASSANDRA: Maybe one that gets your brain more active too?
JIM: Exactly.
CASSANDRA: Now, I just want to make sure. Is that more important to you than trying to improve your sleep?
JIM: Yeah I thought about that too. The sleep bothers me, but even though it takes longer, I still am getting enough sleep. I'd rather focus on doing something positive during the day.
CASSANDRA: There are plenty of options for that! To me it makes the most sense to start with something you know you enjoy. Last week you mentioned you used to read a lot of historical fiction. Is that something you might want to pick up again?
JIM: Yes, I think that would be good.
CASSANDRA: Great! Let's put some thought into what goes into that. I think it would help if I knew - what's kept you from reading lately?
JIM: That's a good question. It sounds silly, but I have a friend who likes the same kinds of books that I do. We used to always trade books. He moved out of state a few years ago so we don't get to see each other much anymore. I just kind of stopped after that. I liked getting recommendations from him and having someone to talk with about the books – that's what made it more fun for me.
CASSANDRA: That doesn't sound silly to me! It sounds like you enjoyed reading, but you also enjoyed having someone to share it with. When you think about getting back into reading, is that social aspect pretty important to you?
JIM: Yeah, I think that's why I wasn't able to stick with it after my friend left.
CASSANDRA: I see. It sounds like for you to get back into reading, we'll need to find a way for you to share that hobby again. What ideas do you have?

© 2026 Abraham J et al. *JAMA Network Open*.

JIM: Well… I'm not usually one for talking on the phone. But I could see if my friend wants to talk books with me from time to time.
CASSANDRA: That sounds like a great idea. How do you feel about it? Can you think of any problems you might run into?
JIM: I suppose he could say that he doesn't have time to talk on the phone, or he hasn't been reading lately either.
CASSANDRA: That's true. Well, there's only one way to find out! I have some other ideas if this reading buddy doesn't work out. I think we're getting closer to making a plan for you to start reading again. It sounds like the first step is making a phone call to your friend, right?
JIM: Yeah, that's right.
CASSANDRA: Sometimes just picking up the phone doesn't feel as easy as it seems like it should. Are you feeling like that at all?
JIM: I know what you mean, but I'm feeling good about this one. It'll be nice to see what he's been up to.
CASSANDRA: Great! So you're going to call your friend before we meet again next week to see if he'd like to stay in touch regularly to discuss books with you.
JIM: Right, and I'll ask him what he's been reading lately so I can pick something up from the library.
CASSANDRA: Great idea. I find when making these plans, it can help to get specific about when exactly you're going to do it. Because sometimes, if we don't plan that out, time can slip away from us. When do you think would be a good time to call your friend?
JIM: That makes sense. I think I'll call him tomorrow morning while I'm sitting outside with the dogs.
CASSANDRA: That sounds like a great plan to me. Some people find it helpful to write down their plans using the "Planning Your Wellness Activities" page in the materials. It might be worth a try. I'm looking forward to hearing how it goes for you!

5. Special Topic: First Post-Op Session

The first session after surgery will typically occur within one week after the patient is discharged from the hospital. The Wellness Partner should ask the patient how they're doing after the surgery, including both the physical and mental aspects of their recovery. This session should include review of the patient's wellness goals in the context of their current abilities and the discharge instructions from their surgeon.
Incorporate this topic into whichever session the patient is due for.

1. Preparing for the Session

- Review the discharge instructions from the surgeon *(Chart review □ Media)*

2. Opening:

Invite patient to retrieve the "What are Your Wellness Goals?" & "Planning Your Wellness Activities" handouts from their materials.

- Review agenda and seek patient's input.
- Check in on the patient's health and recovery.
- Brief symptom assessment.
- If pharmacy team member is not attending the session, ask patient if they have had any medication changes, including OTC. Relay any changes to assigned pharmacy team member as needed.

3. Post-Operative Recovery – Getting Started

1. Ask the patient how they are doing after the surgery, including both physical and mental aspects of their recovery. Validate and address any concerns the patient brings up.
    a. Be aware that for the oncologic surgery patients, the surgery itself and the resulting pathology analyses are often diagnostic as well as interventional. This means these patients are often receiving news about what was found during their surgery and what it means for their prognosis and further treatment. Be prepared to be with the patient if they received difficult news.
2. Re-assess how the patient is doing in these areas and consider activity planning accordingly:

© 2026 Abraham J et al. *JAMA Network Open*.

   a. Worry, stress, anxiety: Consider mindfulness.
   b. Sleep disruption: Sleep hygiene / sleep self-help strategies.
   c. Cognitive concerns or boredom: Brain training
3. Re-visit the “What are Your Wellness Goals?” handout to assess for any shifts in priorities. Check that their goals are compatible with their discharge instructions.
4. Transition into discussing the patient’s wellness goals.

4. Planning Your Wellness Activities

(same as Session 3)
This is a collaborative process between the patient and Wellness Partner. Planning wellness activities should be clear, concrete, and focused on something the patient *does*. Planning activities should consider the difficulty of the task for the patient in their current circumstances. The “Planning Your Wellness Activities” handout should be used as a guide for discussion and for the patient to record their goals.

1. Collaboratively identify activities to work on.
2. Assess the difficulty of the activity in the current context. Break tasks into smaller parts if needed.
3. Develop a concrete plan for engaging in at least one activity. Ideally this plan should identify what the patient is going to do, where, when, and with whom they are going to do it. It is fine to keep the plan more flexible for patients who prefer it. When you follow-up, you can assess whether it would be more helpful to change the level of specificity / flexibility.
   a. The patient may use multiple copies of the “Planning Your Wellness Activities” handout if they would like to plan multiple activities. This can be helpful in later sessions once they have had some success that they want to build on.
4. Ask whether anything could get in the way or make it difficult for them to enact their plan. Brainstorm solutions to these obstacles. Ask whether anything could help make it easier for them to achieve their goal.
7. Discuss how they will record progress towards the goal, using the space provided on the “Planning Your Wellness Activities” handout or another method preferred by the patient. Optionally, some patients may like to record using the “Wellness Journal” handout.

5. Next Session:

Typically, Session 4 would occur in approximately one week.
Encourage the patient in their goal/s and keeping track of the outcome.

First Post-Op Session

CASSANDRA: Jim, I was so glad to see that you made it home on Thursday. How did the surgery go for you?
JIM: The doc says the surgery went just fine and I didn’t have any big problems at the hospital. So I feel pretty good about things even though I’ve been feeling pretty lousy.
CASSANDRA: I’m glad to hear everything at the hospital went smoothly. I’m sorry to hear you’ve been feeling lousy – what’s been going on?
JIM: I mean, I hate to complain. It’s really nothing out of the ordinary. I feel really fatigued and just rough all around. I don’t feel like I can do everything all by myself just yet, which I hate. The pain isn’t so bad as long as I don’t make any sudden movements. I did start to get my appetite back in the past few days which is nice.
CASSANDRA: What I’m hearing is that even though there’s nothing that concerns you about how you’re feeling, it’s still tough to experience it.
JIM: Yeah.
CASSANDRA: How have you been feeling in terms of your mood, anxiety, and emotions? JIM: On the one hand, I feel a big sense of relief because I survived the surgery and it went well. I feel in a very real way that I was given new lease on life. But on the other hand, I have worries that something could still go wrong. And, I feel sort of impatient because I just want to be better already, but I know it takes time.
CASSANDRA: Yeah. That makes sense to me. What you’re going through is really challenging. I have a lot of hope for you that things will get better – but this stage can definitely be hard. Can you tell me more about the worrying that you mentioned? What’s that been like for you?
JIM: It comes and goes. I can usually talk myself out of it, and if not, my wife can.

© 2026 Abraham J et al. *JAMA Network Open*.

CASSANDRA: Would you say that while you've had some worries, it's been manageable? In other words, not a big problem for you?
JIM: Right.
CASSANDRA: One of your other concerns when we talked before your surgery was your sleep. How has that been for you lately?
JIM: Actually, that's been better since I got home from the hospital. I get tired and can fall asleep right away around 10. I do wake up a bit during the night but I'm able to get back to sleep pretty quickly.
CASSANDRA: It sounds like you're getting good rest. I'm glad to hear that! Have you been doing any reading?
JIM: Yeah, I made sure to bring a book to the hospital, although I don't think I even cracked it while I was there. Since I've been home, I've been able to read quite a bit more.
CASSANDRA: That's great to hear. Have you had any trouble focusing while you've been reading?
JIM: Sort of. What I notice is that I can't concentrate too long before I need to just rest my eyes and mind for a little bit. So I might read for 30 or 40 minutes and then just relax for a few minutes before I get back to it.
CASSANDRA: I see. It seems like that may be related to the fatigue you've been experiencing while you recover.
JIM: Yeah, exactly. Even my mind is tired.
CASSANDRA: I think the approach you've been taking with it makes a lot of sense- Reading when you can and resting when you need to. Over time, I bet you'll be able to do more without needing to rest. Keeping your mind occupied will help keep you sharp and it can be a good distraction when you're not feeling your best.
JIM: I hope so.
CASSANDRA: If you're feeling up for it, I'd like to look back at the list of wellness goals we discussed a few weeks ago, before your surgery, and see if any of your priorities have changed. You'd said you wanted to continue spending time with the people in your life. and that you wanted to start working your brain more, pursuing your hobbies, get back to your job, and doing your yard work. At that time, you wanted to prioritize your hobby, fishing, and getting back to a hobby that worked your brain, reading. Has any of that changed for you, now that you're on the other side of your surgery?
JIM: No, that's still what I'd like to do. I just need to be realistic about when I'm able to do some of them.
CASSANDRA: Definitely. Now of those goals, what makes sense for you to focus on now?
JIM: I'm feeling good about the reading, that's been going well so I want to stick with it. I'll keep doing my Sunday phone with my friend. I think what might help me now is trying to reach out a little bit more to my family and friends. I told my wife I didn't want a lot of visitors right away, but now I'm starting to feel like there's a few people I wouldn't mind seeing.
CASSANDRA: That sounds like a great idea! Let's talk more about what that will look like for you.

6. Middle Sessions

Depending on timing, the patient may have up to 12 post-operative sessions. After the initial content is introduced in Sessions 1-3, the remaining sessions have the same format until the final session. Typically, patients have 10 sessions total.
These sessions continue to build upon the patient's activity goals and circumstances. At any point, you can revisit the "What Are Your Wellness Goals?" handout

1. Preparing for the Session

- Review notes from the previous session, in particular the goals the patient set.

© 2026 Abraham J et al. *JAMA Network Open*.

2. Opening:

Invite patient to retrieve the “Planning Your Wellness Activities” handout (and “Wellness Journal” if applicable) from their materials.

- Review agenda and seek patient’s input.
- Check in on the patient’s health and recovery.
- Brief symptom assessment.
- If pharmacy team member is not attending the session, ask patient if they have had any medication changes, including OTC. Relay any changes to assigned pharmacy team member as needed.

3. Structure of session

1. Review and discuss progress on wellness activity planning.
    a. Review progress using the “Planning Your Wellness Activities” handout (or the “Wellness Journal”, or other method used to record progress)
    b. Commend any progress towards the planned activity.
    c. Assess the impact of completing the activity. How did they feel during? After? What does it mean for them that they completed it (or didn’t)?
    d. Identify and problem-solve any barriers to completing the scheduled activity.
    e. If they did not record their activities, see manual section “What if they didn’t track anything?” Continue with reviewing progress and barriers to activity and to tracking.
2. Assess for potential targets for new wellness activity goals:
    a. Ask whether the patient’s daily routines were different than normal.
    b. Ask whether there is anything the patient has needed to do but avoided starting. Are there things you would like to do but that cause difficult feelings or feel overwhelming? Have your emotions gotten in the way of doing anything?
3. Plan new wellness goals (details below)

4. Planning Your Wellness Activities

(same as Session 3)
This is a collaborative process between the patient and Wellness Partner. Planning wellness activities should be clear, concrete, and focused on something the patient *does*. Planning activities should consider the difficulty of the task for the patient in their current circumstances. The “Planning Your Wellness Activities” handout should be used as a guide for discussion and for the patient to record their goals.

1. Collaboratively identify activities to work on.
2. Assess the difficulty of the activity in the current context. Break tasks into smaller parts if needed.
3. Develop a concrete plan for engaging in at least one activity. Ideally this plan should identify what the patient is going to do, where, when, and with whom they are going to do it. It is fine to keep the plan more flexible for patients who prefer it. When you follow-up, you can assess whether it would be more helpful to change the level of specificity / flexibility.
    a. The patient may use multiple copies of the “Planning Your Wellness Activities” handout if they would like to plan multiple activities. This can be helpful in later sessions once they have had some success that they want to build on.
4. Ask whether anything could get in the way or make it difficult for them to enact their plan. Brainstorm solutions to these obstacles. Ask whether anything could help make it easier for them to enact their plan.
5. Discuss how they will record progress towards the goal, using the Planning Your Wellness Activities” or “Wellness Journal” handout, or another method preferred by the patient.

5. Next Session:

Encourage the patient in their goal/s and keeping track of the outcome.
During this time, sessions may continue on a weekly basis or reduce to every other week.

Review and Discuss Progress on Wellness Activity Planning

CASSANDRA: At our last session, you made a plan to invite your son and his family over to visit. How did that go for you?

© 2026 Abraham J et al. *JAMA Network Open*.

JIM: It worked out great. They came over on Sunday. I even helped my wife a little with straightening up and cooking dinner. My son had dropped by a few times before but I hadn't seen his kids since before the surgery. The only sad thing about it is I couldn't do all the things we usually do when they come over. Instead of going out and riding four wheelers all over we just watched the football game.
CASSANDRA: I'm so glad you were able to visit with them. Tell me about the effect that visit had on your week?
JIM: It was nice having something to look forward to. Beforehand, I was a little worried that I would get too tired, or that the kids would get bored inside the house. But during, we just had a nice time together. The kids brought over a couple puzzles and board games. And they go to bed pretty early still anyway so they left at 8.
CASSANDRA: Great job making that happen! It sounds like it was a nice night for everyone.
JIM: Yeah it gives me more confidence that I can start getting together with people again, even if it's just at the house for now.
CASSANDRA: That's wonderful to hear. Now, looking back on what it was like to carry out your plan – was there anything that made it challenging?
JIM: Really just my own worrying. Worrying about being too tired, worrying the kids would be bored. Worrying they were too busy. Worrying my wife wouldn't want the hassle. Come to find out, no one minded at all. They just knew I'd wanted more privacy in those early days so they were trying to give it to me.
CASSANDRA: I see. So, what helped you to overcome the worrying and start getting the plan together?
JIM: I started off by talking to my wife about it. Once I had her reassurance, it was easy.
CASSANDRA: I'm glad she able to help you get past your worries. Now, you planned to continue with reading for an hour each day and talking to your friend once a week. How did that go for you?
JIM: That one's been pretty good I think.
CASSANDRA: Yeah? Did you keep track like we talked about? How many days did you read for at least an hour?
JIM: Oh. Uh, no I didn't really write anything down. I read a lot most days but I might have missed a few when we had more going on or I didn't feel so good. I haven't missed a Sunday calling my buddy though. We usually talk for 20 minutes or so.
CASSANDRA: That's wonderful to hear that you've kept up with your friend and your reading. How has that been for you?
JIM: It's been good. I definitely like it as part of my morning and evening routine to do some reading. I started thinking I'd also like to see if I can find some good books on local history and start learning more about my family history. I think I've got a cousin who is interested in that.
CASSANDRA: It's amazing to hear that you've fit this new hobby into your life, and that it is growing and getting you interested in new things. It sounds like there where a few days where you weren't able to read like you planned. Can you tell me more about those days?
JIM: Yeah I think it was just that on Sunday my wife sat with me in the morning and we planned out who was going to do what to get ready. Then in the evening I was worn out so I just went straight to bed.
CASSANDRA: That makes sense that you didn't have much time or energy to read on that day. Were there any other days where you either didn't read at all?
JIM: I did have one other day since I last talked with you. My wife went over and spent the day with some of our friends. I wanted her to go, but I couldn't go with her because I'm still not really supposed to make any unneeded trips. All day I just was in a funk and it made me feel so childish too. I mean, it shouldn't be a big deal. But I just felt so down on myself and discouraged. Poor me, and all that.
CASSANDRA: That sounds like a hard day.
JIM: Yeah it was. I realized that's the only time I knew for sure I was missing out on something. Of course, I realize people's lives are going on without me, but I wasn't really aware of it at the time it was going on like that.
CASSANDRA: That's a tough feeling – to feel left out and discouraged. I can tell you're really yearning to be living your normal life again.

© 2026 Abraham J et al. *JAMA Network Open*.

JIM: That's it exactly. Most days, it's ok and I know it's just temporary. I can see how much better I've gotten and am so grateful to have my heart working better again. That day was just a rough one for me.
CASSANDRA: Yeah definitely. So I wonder – what kept you from reading? Do you think it would have helped you to feel better?
JIM: I think it probably would have. But I just honestly didn't feel like doing much of anything. I kind of had in mind that I wanted to be productive and do some of the things around the house that have been stacking up. But I just really couldn't focus well. I ended up watching some TV and taking a nap.
CASSANDRA: I see. So to sum up, it sounds like reading has been going well. One day you missed for a positive reason, and one day you missed because of difficult emotions.
JIM: That's right.

Review and discuss experience with tracking planned activities

CASSANDRA: Let's talk a little about keeping track of your progress with reading. What happened with that?
JIM: Nothing happened, that's the problem, I guess. I got your sheet right here on the side table where I put it after our last session. I'd think of it from time to time but I think I do ok just keeping it in my head.
CASSANDRA: You do have a pretty good memory! Writing it down can help you to better see your progress and patterns and can help you to see when things are interfering with your goals. I bet some days you're reading much more than an hour – it'd be great to know that for sure!
JIM: Ok, I could try that again.
CASSANDRA: Is there anything you can think of that might make it easier for you to do that?
JIM: Yeah, I think I might just keep a notepad and pen by my book. That way I can keep the log like you said, and also write down if there's something I want to be sure and mention when I talk to my friend.
CASSANDRA: That sounds like a good plan. I can't wait to hear how it goes. So I feel like I heard pretty well about a high point and low point from the last few weeks. Were there any other days you wanted to tell me about?
JIM: No, the rest were pretty normal.

Assess for potential targets for new wellness activity goals

CASSANDRA: Got it. I think by now I know your routine pretty well. In terms of how you're spending your time, it sounds like I could usually find you: reading, sitting outside, watching TV, reading news stories, and if it's Sunday, talking to your buddy. Anything else?
JIM: Well, I try to help out just keeping up with dishes, folding clothes, and taking care of the bills and mail. Fixing some basic meals. That kind of thing.
CASSANDRA: Ok, great! Is that a change since the last time we talked?
JIM: Yeah I guess it is. I've just been trying to do a little more here and there to take some of the stress off my wife and feel like I'm useful again.
CASSANDRA: That sounds like big progress to me. How do feel when you're doing those tasks?
JIM: It feels good, and I do notice some things are starting to get easier. I'm glad I can take some of the load off my wife because she has helped me a lot over the past few months.
CASSANDRA: I bet she's happy for many reasons you're able to do those things again! Tell me about how your mood has been with your other activities.
JIM: I always feel best when I'm outside reading. And of course if I'm visiting or talking with family or friends. With TV, I switched to just watching classic movies, sports, and the nightly news. I finally figured out I don't like flipping channels or watching anything else really. So if none of those are on, I turn it off. Now I don't get so bored and irritated watching TV anymore. And I don't read as many news stories since I get most of it by watching the nightly news. I will sometimes look up something if I'm interested to read more.
CASSANDRA: Wow, so I'm hearing that you've made some little tweaks to some of those things that lowered your mood so that you're able to enjoy them again?
JIM: Yeah, you could say that.
CASSANDRA: Great thinking! Besides those changes, were there any ways that your daily routines have been different than normal?
JIM: No, I think that's about it.

© 2026 Abraham J et al. *JAMA Network Open*.

CASSANDRA: And, is there anything that you've needed to do but had a hard time starting on?
JIM: Nothing really urgent, but while we were talking I did realize I've been kind of putting off seeing most of our friends. I think I am finally feeling up for it. We have two other couples we're pretty close with and I think it'd be nice to have them over soon.
CASSANDRA: That's a great idea! That brings us to our next topic – planning new wellness goals for the next few weeks.

7. Final Session

The final session occurs approximately 3 months after the date of surgery. The final session is typically Session 10, but if the patient reaches 10 sessions and is not yet 3 months post-op, continue with bi-weekly sessions and number sequentially up to a maximum of 12 post-operative sessions.

1. Preparing for the Session

- Review notes from all sessions and progress towards goals.

2. Opening:

Invite patient to retrieve the "Planning Your Wellness Activities" handout (and "Wellness Journal" if applicable) from their materials.

- Review agenda and seek patient's input.
- Check in on the patient's health and recovery.
- Brief symptom assessment.
- If pharmacy team member is not attending the session, ask patient if they have had any medication changes, including OTC. Relay any changes to assigned pharmacy team member as needed.

3. Structure of session

1. Review the wellness activity goal/s the patient set at the previous session.
2. Review progress from initial session. Commend progress.
3. Ask how the progress has impacted the patient's life, emotions, and relationships. Or in other words, what have those changes meant for the patient? Provide reinforcement and encouragement.
4. Discuss what practices the patient would like to 'keep,' and address any barriers to doing so.
5. Assess need for ongoing mental health treatment and assist with referrals. Refer the patient back to the resources provided and discuss if any additional resources or referrals will be helpful.

Review Progress from Initial Session and Discuss Impact of Progress

CASSANDRA: I was just looking back through my notes at all of the progress you've made since our first session together. What has stood out for you?
JIM: I think I'm the most pleased with how the reading has really taken off. And not just because I enjoy reading. I'm really happy I was able to get back in touch with my friend. Before, I wouldn't have called him up just because, but now we talk all the time. I also got to talking with my cousin who does all this genealogy and family history stuff. We're going to get together soon so he can show me what he's been working on.
CASSANDRA: It's amazing how this one change has led to other positive changes and even strengthened some of your relationships. It's so incredible you were able to add something new in your life, even when you weren't feeling your best.
JIM: Ha, well that's true. But honestly, if I wasn't feeling bad I probably would have never slowed down enough to do any of that.
CASSANDRA: I'm so glad that you found a meaningful way to use all of this recovery time. Looking back, one of the big reasons for setting the goals around reading in the first place was because you felt your thinking wasn't at its best. You told me you were having a hard time focusing and just feeling sort of fuzzy. Has that changed for you?
JIM: Yeah that has definitely gotten better over time. The real test will be when I go back to work next week.

© 2026 Abraham J et al. *JAMA Network Open*.

CASSANDRA: Getting back to work – that was a big reason you wanted to feel sharp again. You've made so much progress mentally and physically since your surgery. How are you feeling about the progress you've made?
JIM: I'm really happy with the progress I've made. In some ways I feel better than I did before the surgery. I'm still not quite where I'd like to be, especially in building back my strength and stamina, but I can see myself getting there.
CASSANDRA: I noticed that the other activity plans you made, besides reading, were about reconnecting with the people in your life. How have you been feeling about the progress you've made in reconnecting socially?
JIM: That has been interesting. One thing I realized is that I like to be in control of how people see me. There's only a small group who I allowed to see me when I was at my worst. I didn't realize that mattered to me but… I guess it does. So reconnecting was a bit of a process because I wanted to feel more like myself again before I was around certain people. I've been seeing more people now that I've been feeling better and can go out again, and it actually feels pretty normal. It feels like I'm getting my life back and can actually appreciate it more.
CASSANDRA: Wow, Jim, thank you for sharing that with me. When you think back on the changes you've made during your recovery – what have they meant for you in your life?
JIM: Doing so much more reading has shown me that I don't always have to be busy doing something. I am excited to get back to doing the things that I used to but now I know I can relax for a day and it's not the end of the world.

Practices to Continue

CASSANDRA: Now that we're reaching the end of our time together, I'm curious if you'll continue with any of the changes that you've made? Is reading something you'd like to keep up with once I stop asking about it?
JIM: Yeah definitely, because my friend will still be asking me about it too. I won't have as much time during the week, but definitely on the weekend.
CASSANDRA: Besides being busy with work, can you think of anything that could make it hard for you to keep up with reading?
JIM: Well, maybe getting in a rut with finding new books to read. But I ended up meeting one of the librarians the other day and I think he'll have some good recommendations for me too.
CASSANDRA: It sounds like you've got a good plan for making sure reading can remain a part of your life! What about the social goals you had set? Is making plans with people something that will continue to be important to you?
JIM: Yeah, but honestly, that's already getting a lot easier. We're starting just to get back in our normal kinds of things. It was harder when I couldn't participate in everything, but now that I can, it comes a lot more naturally.

Assess Needs and Provide Referrals

CASSANDRA: You've really shown so much resilience and strength and I've enjoyed hearing about your recovery and your life. My last task is to make sure you feel well supported in your mental health going forward. I haven't heard anything from you that makes me concerned that you're in urgent need of mental health care. Even so, I'd like to ask – do you feel you could benefit from a referral for a psychiatrist or therapist?
JIM: I don't really think that's something I need right now. But if I do, I'll ask my doctor about it.

8. Key intervention skills

1. Demonstrate compassion

Compassion has been defined by health researchers as "a virtuous response that seeks to address the suffering and needs of a person through relational understanding and action.[1]" In the Wellness Program, compassion forms the foundation on which the other elements can be built. Wellness Partners can demonstrate compassion during the Wellness Program in the following ways:

- Convey a genuine interest in the patient as a whole person.

---

[1] Sinclair S, McClement S, Raffin-Bouchal S, et al. Compassion in health care: An empirical model. Journal of Pain and Symptom Management. 2016;51(2):193-203. doi:10.1016/j.jpainsymman.2015.10.009

© 2026 Abraham J et al. *JAMA Network Open*.

- Ask open-ended questions.
- Seek to understand the patient's experiences from their perspective.
- Choose language that is kind, warm, and supportive.
- Summarize what you have heard from the patient to check for and convey understanding.
- Show empathy and validation for experiences that are difficult. Allow space to experience and process difficult emotions.
- Celebrate every accomplishment, any amount of progress, and any good news. Be encouraging (a cheerleader).
- Normalize the patient's experiences. There are a wide range of normal emotional and behavioral reactions during the surgical period. Whatever the patient is going through, it makes complete sense.
- Promptly validate and address any concerns the patient brings up.
- Visiting the patient while they are in the hospital is an important way to show compassion and enhance connection.

2. Problem solving

The surgical period can present new challenges for patients, while stress, fatigue, and pain can compromise their normal problem-solving capacities. Patients may bring up such problems while sharing updates about their week, or when developing and discussing activity plans. Some common types of problems during the surgical period are difficulties navigating the health system to receive needed care, difficulty managing activities of daily living, and financial difficulties. However, other types of problems can arise during the surgical period, and are made more difficult by the timing.

When a patient brings up a problem they are having, the first thing to do is actively listen and validate the patient's experience. Follow the patient's lead when it comes to discussing potential solutions. Sometimes when discussing a frustrating problem or one with emotional weight, patients just want to express themselves and feel heard. They may not be ready to discuss solutions, or they may already have a plan for solving the problem. If unclear, ask whether they would like to talk through some ideas for solving the problem.

If patients would like to discuss potential solutions, continue with a structured problem-solving approach[2]:

1. **Identify the problem:** Work together to define the problem as concretely and narrowly as possible, making sure to see the problem from the patient's perspective. Discuss what the patient has done already to try to solve the problem.
2. **Brainstorm potential solutions:** During brainstorming, all ideas are listed out without evaluation. Even if an idea is very clearly not preferred or not feasible, listing it out while brainstorming helps expand the creative process of idea generation. Initial ideas should be generated by the patient. The Wellness Partner can contribute a few ideas. It helps if the patient or Wellness Partner write down the ideas generated.
    a. **Ideas for problems with navigating the healthcare system:** For issues related to the surgery, the primary point of contact is the surgeon's office. For health conditions being managed by a specialist, the patient should contact that specialist (e.g., oncologist, cardiologist). In other cases, the patient's primary care physician can field and direct any questions. Sometimes patients have difficulties getting responses back from doctor's offices. In this case being persistent can help as well as trying different times of day and different methods (e.g., using the patient portal).
    b. **Ideas for managing activities of daily living:** Patients may have very short-term difficulties with some tasks that can be overcome with some additional assistance from a caregiver or home health worker. Others may benefit from evaluation and sessions with a physical therapist or occupational therapist. A home health worker can assist with things like bathing, wound care, and taking medications. Their surgeon can assess these needs and refer them for these services. Patients may also want to explore options for assistance with household chores.
    c. **Ideas for financial difficulties:** Medical bills and taking time away from work can cause financial strain. Patients whose main difficulty is medical bills can contact the billing office to discuss a payment plan for medical bills. For patients whose needs are more widespread, they may be referred to inpatient or outpatient social work to assess needs and connect with resources. A few

[2] Lorig K, McGowan PO, Huebner TLB. Living a Healthy Life with Chronic Conditions Self-Management of Heart Disease, Arthritis, Diabetes, Depression, Asthma, Bronchitis, Emphysema and Other Physical and Mental Health Conditions. Boulder, CO: Bull Pub. Co.; 2013.

© 2026 Abraham J et al. *JAMA Network Open*.

key resources in the US are United Way (dial 211) and Area Agencies on Aging (1-800-677-1116).

3. **Rank top solutions**: The patient should identify what they feel is the best potential solution for solving their problem. Then, identify a 'Plan B' in case the top choice does not end up working (and potentially, a 'Plan C').
4. **Make a plan:** Guide the patient in developing a plan to enact the top solution. This process is similar to activity planning. The plan should be specific and feasible in the patient's current circumstances. Discuss barriers and facilitators.
5. **Enact the plan:** Hopefully, the patient will enact the plan after the session.
6. **Assess:** Find out if the patient enacted the plan and if it solved the problem. If it did not, revisit step 4 and adjust the plan, or revisit step 3 and move on to the Plan B. If the problem persists after a few attempts, revisit step 2 and identify additional resources for generating potential solutions. This process can be repeated as needed, but ultimately, some problems may be unsolvable. Although this can be difficult to accept, going through a systematic process together can help patients feel as though they've at least given their very best attempt.

3. Motivation for Change

In working with the patient during the surgical period, a need for the patient to change a behavior may become apparent. The principles of motivational interviewing, summarized from Miller et al, 1992[3] are helpful for discussing attitudes towards change in a collaborative, non-confrontational way. Motivational Interviewing does not assume that a patient is already motivated to change their behavior; the goal is to evoke and build the motivation for change. Patients often have ambivalence towards change: there are reasons their current behaviors have persisted so far unchanged. This is normal and should be discussed openly without judgement.

- **Express empathy:** Meet the patient where they are, with the understanding that they (and only they) possess the capacity to change. The Wellness Partner's role is to support the patient in the process of change while respecting their freedom of choice. Reflective listening is an important way of expressing empathy.
- **Develop discrepancy:** Change can occur once a patient realizes there is a discrepancy "between where they are and where they want to be." This realization causes discomfort that the patient may seek to resolve by changing their behavior. The Wellness Partner can help to develop this discrepancy by sharing observations and asking about consequences of the patient's behavior should they continue unchanged.
- **Avoid argumentation:** Direct persuasion often leads to defensiveness, where the patient takes the role of arguing against change. This is damaging to the relationship because it shows the patient the Wellness Partner really doesn't understand where they are coming from. Ultimately, this leads to more resistance to changing behaviors. Instead, the Wellness Partner should evoke from the patient their own reasons for change.
- **Roll with resistance:** Resistance to the Wellness Partner's recommendations or requests can occur. Resistance can be reduced or magnified by the Wellness Partner's response to resistance. Avoid confrontational strategies that attempt to meet the resistance head on (such as persuasion, judgement, or arguing). Instead, show the patient you hear what they are saying and gently invite alternative perspectives.
- **Support Self-Efficacy:** In order to change, the patient must believe that they can. The Wellness Partner should support and encourage the patient in developing the hope that they can change their behavior.

4. Working with the patient's family and caregiver

The Wellness Program is designed as a one-on-one intervention with the patient. There are opportunities to engage a family member / caregiver according to the patient's preferences and goals. Family members and caregivers can play in important role in supporting the patient's wellness goals. In some cases, patients may need direct support from a family member or caregiver in order to carry out their activity plans (e.g., transportation). In other cases, involving another person in an activity plan can provide accountability and make it more enjoyable. Typically, the

[3] Miller WR National Institutes of Health (U.S.) National Institute on Alcohol Abuse and Alcoholism (U.S.). Motivational Enhancement Therapy Manual : A Clinical Research Guide for Therapists Treating Individuals with Alcohol Abuse and Dependence. Rockville Md: U.S. Dept. of Health and Human Services Public Health Service National Institutes of Health National Institute on Alcohol Abuse and Alcoholism; 1999. http:://purl.fdlp.gov/GPO/gpo37581. Accessed September 8 2022.

© 2026 Abraham J et al. *JAMA Network Open*.

Wellness Partner will encourage the patient to have these discussions with their family members or caregivers. However, if a need arises, a family members or caregiver can join a portion of a session at the patient's invitation.

5. Working with the Pharmacy Team

The role of the pharmacy team is limited to the scope described in the SOP: reducing the use of problematic medications that increase risk for falls and delirium in older adults and increasing sub-therapeutic doses of antidepressant medications. It is important to use clear and precise language about the role of the pharmacy team member when speaking with the patient. Here are some examples:

**YES, say this**	**NO, don't say this**
I have a member of our pharmacy team, Jane, on the call with us to:<br>- *Review your medications to look for any that could be affecting your brain or mood*<br>- *Provide an update about the plan you discussed last time.*	*I have a member of our pharmacy team, Jane, on the call with us to:*<br>- *Go over all of your medications to make sure they are safe for you to take*<br>- *Answer any questions you have about your medications and any side effects you may be having*
Do you need to speak with the pharmacist today about the plan to lower your dose of oxybutynin?	*Do you have any questions about your medications for the pharmacist?*
That is a good question about whether you are receiving the correct dose of your heart medication. I don't know the answer but we could make a plan for how to get it sorted out. What have you tried so far?	*That is a good question about whether you are receiving the correct dose of your heart medication. I will ask our pharmacy team to find out for you.*

9. Special considerations for surgical patients

1. Orthopedic Patients

The orthopedic population includes patients scheduled for total knee arthroplasty or total hip arthroplasty. The primary diagnosis for these patients is typically osteoarthritis. These patients often wait up to several months before their surgery occurs. The typical hospital duration is quite short, with most patients being discharged 1 day after surgery. The orthopedic department asks patients to identify a "joint coach" or primary caregiver and attend a joint education class to prepare for surgery. Patients typically receive outpatient physical therapy and complete exercises at home to strengthen around the new joint. Patients generally can expect improved mobility 1-2 months following surgery, although they may still experience limitations if other joints are also affected by osteoarthritis.

2. Cardiac Patients

The cardiac population includes patients scheduled for major cardiac surgery. The most common procedures include coronary artery bypass grafts (also known as CABG or bypass surgery), and valve replacement. The primary diagnosis for these patients is typically coronary artery disease. These surgeries typically occur 2-3 weeks after being evaluated in clinic. Patients routinely spend a few days post-operatively in the ICU, then typically spend another 4-5 days in the hospital before being discharged. Patients are often discharged home to be cared for by family members. Patients without a family caregiver or who became debilitated may be discharged to a rehab facility. Patients usually follow up with their surgeon 4 weeks after surgery and also follow up with their cardiologist and primary care provider. Some patients may be recommended for a cardiac rehabilitation program.

3. Oncologic Patients

The oncologic population includes patients scheduled for removal of different types of tumors, masses, and cysts. In some cases, the patients have a cancer diagnosis already, but in other cases a diagnosis will be determined after pathology analysis. These surgeries typically occur approximately 3-4 weeks after a clinic consultation, but sometimes the wait is longer. The average patient is driving from 3 hours away. Surgeries are complex and often last 8-10 hours. Most patients stay in the hospital between 3 and 7 days post-operatively, but a significant proportion will stay for 1 to 2 weeks. Some patients receive courses of chemotherapy before undergoing surgery, and patients may begin chemotherapy as early as 4-6 weeks after surgery. Patients will follow up with an oncologist. The prognosis is highly variable. Some patients will experience significant complications and spend 1-2 months in the

© 2026 Abraham J et al. *JAMA Network Open*.

hospital and rehabilitation. More typically, patients can expect a complete recovery 6-8 weeks following surgery. As noted in the “First Post-Op Session,” oncologic surgery often serves both interventional and diagnostic purposes. This means that post-operatively these patients are often receiving news about what was found during their surgery and what it means for their prognosis and further treatment. Be prepared to be with the patient if they received difficult news.

4. Visiting Patients in Hospital

Patients have expressed appreciation for a visit from the Wellness Partner during their hospitalization. Often this is the only opportunity to meet in person and doing so enhances connection. It’s important to check with the patient ahead of time to provide the opportunity to decline a hospital visit. For patients in the ICU, make sure to check with their nurse before visiting. Patients will not typically have enough time, energy, or privacy for a formal Wellness Session while they are hospitalized. Rather, the purpose of this visit is to show interest in how the patient is doing. If the patient is having any difficulties during the hospitalization, the Wellness Partner can help to problem solve and identify the appropriate person on the patient’s care team to assist.

10. Addressing Common Questions and Concerns

1. Making Activity Plans:

- **“I like to just do things when I feel like it.”** Use this as an invitation to discuss the concept of acting according to a plan vs a feeling, while acknowledging that acting according to a feeling may work perfectly well for them for some or all activities. For example, a Wellness Partner might say: *“This is ok if it works for you. But if you find that there are things that are important to you that you never ‘feel like’ doing, or don’t feel like doing as often as you’d like to, we can try a different approach for those things. You might find when you act according to a plan instead of a feeling, over time, you feel like doing the activity more often.”*
- **“I lost the materials.”** This happens a lot because some patients are away from home for a long time and have help from others retrieving and sorting mail. Just send them again.
- **“I can’t physically do the things that I really want to do**.” Show empathy to the patient – this is a difficult experience. Explore the activity and the barriers to engaging in it with the patient. Are there other ways of engaging with the hobby that the patient is able to do now (and would enjoy)? If not, learn about the function of this activity for the patient and explore alternatives. What is it that they got out of it and enjoyed about it? Can they try out any new activities to seek out a similar experience? This can take some trial and error, and creativity.
- **“My only goal is to lose 50 pounds / walk again / get cleared to go back to work.”** These are examples of an outcome–based goal. Help the patient focus on what they can do to achieve that goal, starting from where they are now. Assess whether other resources are needed to achieve the goal.
- **“I want to watch less TV / call my friends more**.” These are great goals to strive for but the frequencies are vague. Assess how often the patient is doing these things now, hopefully using their activity log to record. Try to set a clear, measurable reasonable goal for moving forward and a plan for measuring progress. If the patient wants to decrease or stop certain activities, it can help to set goals for alternative activities.
- **“What do you mean by ‘mental health’ anyway? How could that be more important than this operation I’m having?”** This provides an opportunity to convey an integrative approach to health and well-being. For example, a Wellness Partner might say: “*What we mean by mental health is health in terms of one’s mental state, emotions, and thinking. Mental health can influence physical health, but it can get overlooked in medical settings. We want to make sure we pay attention to your mental health, and how it is related to your physical health, so that you can live a full, healthy, happy life.”*
- **“Are you saying I’m a depressed person? I’m not crazy or anything.”** Sometimes patients will make comments that reflect internalized stigma against people with mental health conditions. It is important to note that while the Wellness Program was developed for surgical patients with elevated depression and/or anxiety *symptoms*, it is not designed to assess or treat a specific mental health *disorder.* Feelings of depression and anxiety are normal reactions to stressful situations like surgery, but they can negatively impact health and quality of life. We want to offer support not because we think there is something wrong with them or that they are incapable, but because the circumstance is challenging.

© 2026 Abraham J et al. *JAMA Network Open*.

2. Completing Activity Plans:

The overall approach when there are barriers to achieving activity plans is first to normalize. These things happen, and changing what we do can be very hard. Then, seek to understand what happened. Finally, invite discussion of possible solutions to achieve a favorable result next time. The type of solutions the Wellness Partner suggests should be based on the reasons the patient gives for not achieving the goal.

- **"I didn't remember"** or **"I didn't remember at the right time."** This can happen when the new behavior is not cued by anything in the patient's environment. Try asking "*Can you think of anything that could help you remember?*" Solutions could include a cell phone reminder, visual reminder, or arranging the environment to cue the behavior (for example, setting the materials in plain view). This is most sustainable when the patient implements the reminder, but if necessary, the Wellness Partner can offer a reminder between sessions.
- **"I don't know how to do that."** The patient may lack skills, knowledge, or experience to engage in the new behavior. They may express this with reluctance or feelings of uncertainty about whether the new activity is for them. This just means that the activity plan needs to include ways for them to build the necessary knowledge. Try asking questions like "*What do you know about ___? How do you usually like to learn new things? Do you know anyone who could show you how?"* Solutions would need to be tailored to the activity but could include visiting the library, watching some YouTube videos, or consulting an expert.
    - Note that deficits in social skills may interfere with or inhibit some activities but may not be explicitly stated by the patient. In the case that social skills interfere with planned activities, sessions can incorporate instruction in social skills, modeling, role-playing, and assigning real-world practice.
    - Patients may also lack skills in problem-solving, or they may encounter a problem that they don't know how to solve. In this case, structured problem solving can be done in session (refer to Key Intervention Skill: Problem Solving section).
- **"I was too tired / hurting too badly."** Physical pain and fatigue are common in medically ill surgical patients. Pain and fatigue may be caused by the surgery, anesthesia, and co-morbid medical conditions. Anxiety, depression, and stress also contribute to pain and fatigue. Overexertion and inactivity can both exacerbate pain and fatigue, so achieving a balance of rest and activity is crucial. Refer patient to the Activity-Rest Cycle handout. Discuss pain and fatigue symptom cycles as they relate to the patient's experience. Discuss activity - rest cycle (pacing) as an alternative: alternating planned activity with regular rest periods. Keep track of activity and rest periods and their impact on pain and fatigue levels using the activity pacing form or another method the patient prefers. Prolonged pain that interferes with daily activities should be assessed by their medical provider.

3. Tracking Progress: What if they didn't track anything? What if they don't want to track?

Often, patients will state that they did not keep track of their progress on their activity plans. Patients provide different reasons for this, from forgetting, to losing the form, to just not wanting to do it. It's important to continue to emphasize the importance of keeping some sort of record, while providing flexibility on exactly how the patient carries it out. Keeping a log between sessions allows for more accuracy in tracking progress towards goals and can help identify patterns and barriers. The specific format and methods for doing so should be adapted to the patient's habits and preferences. Keeping a written log relates to the empirical problem-solving approach of Behavioral Activation, where we encourage the patient to try something out and any resulting information is useful. If the first introduction of tracking progress is not successful, revisit it later on in the program once the patient has some experience making activity plans. It may be helpful to work with the patient to develop a specific plan to help them with activity logging. Where can they put the materials to make it convenient? What can they do to remind them to record their progress? When in their day would be a natural time to do it?

11. Sequencing variations:

The duration of the Wellness Program can vary due to variations in the pre-operative period; however the post-operative period is limited to 3 months. Here are some suggestions for pacing the intervention in various circumstances:

- **Very short pre-operative period:** It is relatively common that there is only enough time for 1 pre-operative session. In this case, just conduct Session 1, assess the patient's interest in a hospital visit, and continue with Session 2 along with the first post-op session content.

© 2026 Abraham J et al. *JAMA Network Open*.

- **No pre-operative sessions:** Sometimes the pre-operative period is so short there is not enough time for any pre-operative sessions. In other cases, patients are not available or interested during the lead-up to their surgery. This happens most with patients who have significant work or caregiving obligations. In these cases, make an effort to reach the patient to introduce yourself. If you are able to reach the patient, ask whether they would appreciate a hospital visit. If you are not able to reach the patient, consider a hospital visit anyway. Then begin with Session 1 after the patient is discharged from the hospital.
- **Long pre-operative period:** Some patients, especially orthopedic patients, may have 1-2 months of lead time before their surgery date. As long as the participant is engaged, conduct up to a maximum of approximately 6 sessions ahead of the surgery, then continue afterwards. If the participant is not well engaged, you can propose re-connecting after the patient's surgery and continuing then. More typically, 2-4 pre-operative sessions would be adequate.
- **Postponed/rescheduled surgery:** Surgeries can get postponed and rescheduled for clinical and non-clinical reasons. It's good to check in with the patient to discuss their feelings about this as it can be frustrating to experience. As long as the participant is engaged, conduct up to a maximum of 8 sessions ahead of the surgery, then continue afterwards.
- **Withdrawals due to cancelled/postponed surgery**: These RCTs will use the modified intention-to-treat approach and participants will be withdrawn from the study if their surgery is cancelled or postponed (for clinical or non-clinical reasons) for more than approximately 3 months after baseline. These participants will also be withdrawn from the intervention. Participants will not be withdrawn until the approximately 3-month timepoint and can continue intervention sessions until that time. If it is determined that the patient will not undergo surgery before the 3-month timepoint before intervention sessions start, the Wellness Partner does not need to start intervention sessions.
- **Re-hospitalization or additional surgery:** These kinds of complications can be very difficult and discouraging for patients. Allow time to sit with negative emotions. Hospital visits can be a particularly helpful way of offering support, or the patient may prefer just brief phone calls to check in. Continue with regular sessions once the patient is discharged again, re-visiting goals in their changing circumstances as in the first post-op session.
- **Unsuccessful surgery:** In some cases, the surgeon attempts but is not able to accomplish the goals of the surgery. This means that, on top of recovering from surgery and anesthesia, these patients also have to cope with the disappointment and prognostic implications of the failed surgery. Continue with the Wellness Program after discharge, allowing plenty of time to process their experiences and emotions.
- **Patient unreachable for sessions:** Make reasonable attempts to contact patients for their sessions, leaving a voicemail if you are unable to reach them. Try their alternate number as well, if provided. If participant has email and consented to email contact, send them an email. If several attempts are unsuccessful, send a "trying to reach you" letter (see appendix for template). Log each attempt at contact. The session content of the early sessions builds on each other, so once you are able to re-connect with the patient, continue with the next session in the sequence (do not skip sessions).

12. Appendix: Assessments and Materials

1. PHQ ADS items and interpretation

This assessment is conducted by research staff at Baseline, 1 month post-op and 3 months post-op. This measure combines two instruments, the PHQ-9, which assesses depression symptoms, and the GAD-7, which assess anxiety symptoms. Patients with an elevated combined score are eligible for the study.
The patient's responses and scores should be reviewed by the Wellness Partner before Session 1.

© 2026 Abraham J et al. *JAMA Network Open*.

PHQ-9 Depression

**Over the last 2 weeks, how often have you been bothered by any of the following problems?** *(Use "✔" to indicate your answer")*	**Not at all**	**Several days**	**More than half the days**	**Nearly every day**
1. Little interest or pleasure in doing things................	0	1	2	3
2. Feeling down, depressed, or hopeless.................	0	1	2	3
3. Trouble falling or staying asleep, or sleeping too much..	0	1	2	3
4. Feeling tired or having little energy.......................	0	1	2	3
5. Poor appetite or overeating..................................	0	1	2	3
6. Feeling bad about yourself — or that you are a failure or have let yourself or your family down......................	0	1	2	3
7. Trouble concentrating on things, such as reading the newspaper or watching television............................	0	1	2	3
8. Moving or speaking so slowly that other people could have noticed? Or the opposite — being so fidgety or restless that you have been moving .around a lot more than usual..	0	1	2	3
9. Thoughts that you would be better off dead or of hurting yourself in some way...	0	1	2	3

© 2026 Abraham J et al. *JAMA Network Open*.

GAD-7 Anxiety

Over the **last 2 weeks**, how often have you been bothered by the following problems? *(Use "✔" to indicate your answer")*	Not at all	Several days	More than half the days	Nearly every day
1. Feeling nervous, anxious or on edge	0	1	2	3
2. Not being able to stop or control worrying	0	1	2	3
3. Worrying too much about different things	0	1	2	3
4. Trouble relaxing	0	1	2	3
5. Being so restless that it is hard to sit still	0	1	2	3
6. Becoming easily annoyed or irritable	0	1	2	3
7. Feeling afraid as if something awful might happen	0	1	2	3

PHQ-9 Total scores are interpreted as follows:

0-5 = Mild depression severity
6-10 = Moderate depression severity
11-15 = Moderately severe depression
16-20 = Severe depression

GAD-7 Total scores are interpreted as follows:

0-5 = Mild anxiety severity
6-10 = Moderate anxiety severity
11-15 = Moderately severe anxiety
16-21 = Severe anxiety

- Note whether anxiety or depression are more prominent for the patient.
- Note which symptoms are reported at the highest frequencies and discuss these prominent symptoms with the patient. Activity plans could be developed to alleviate the most bothersome symptoms.
- Note whether the patient reported any suicidal thoughts (#9 of PHQ-9). Follow up on this at session 1 to learn the details and context and develop a safety plan (see Suicide Safety Procedures). These patients should be assessed for suicidal thoughts at each session using question 5 of the Brief Symptom Assessment.

2. *Brief symptom assessment*

The Brief Symptom Assessment is designed to initiate discussion about the patient's mood and anxiety symptoms during the previous week and serve as a low burden way of assessing progress during the Wellness Program. Be sure to acknowledge the patient's experiences and use their responses in discussions about the patient's mood and activities.

1. Overall, how would you say your mood has been during the past week?

2. During the past week, have you been feeling anxious, worried, or stressed? If so, how bad has that been for you, on a scale from 0-10, where 0 is not at all and 10 is the very worst it could be?
If patient reports they have not been feeling anxious at all, record 0.
If patient can only provide a range and is unable to select a number, record the highest value and note details below.

0 1 2 3 4 5 6 7 8 9 10

   a. Anxiety details (optional):

3. During the past week, have you been feeling depressed, down, sad, or blue? If so, how bad has that been for you, on a scale from 0-10, where 0 is not at all and 10 is the very worst it could be?
If patient reports they have not been feeling depressed at all, record 0.
If patient can only provide a range and is unable to select a number, record the highest value and note details below.

0 1 2 3 4 5 6 7 8 9 10

   a. Depression details (optional):

4. What do you think contributed to your mood and emotions this week?

For participants with suicidal ideation at baseline or subsequently:

5. Have you had any thoughts of suicide during the past week?

☐ Yes *If yes, continue with suicide risk assessment.*
☐ No
☐ Not assessed (no SI reported at baseline or subsequently)

3. Suicide safety procedures

Patients are assessed for the presence of suicidal thoughts at Baseline using the PHQ ADS. If a patient endorses suicidal thoughts on the PHQ ADS at baseline, they should be assessed for suicidality at each session using item 5 of the brief symptom assessment.
When a patient endorses or spontaneously reports suicidal thoughts, it is crucial to assess the risk and respond accordingly. The "Quick Suicide Safety Screening" is designed as an efficient method to assess suicidal risk. Additional probes and clarifications may be needed.

Quick Suicide Safety Screening

(Ask these questions if patient scores above a 0 on PHQ-9 item 9; or if patient spontaneously reports suicidal thoughts)

1. Do you currently have suicidal thoughts or thoughts of killing yourself?
____
____

If NO, STOP! Risk is LOW, follow action plan below.

© 2026 Abraham J et al. *JAMA Network Open*.

a. **If YES,** have you thought of how you would kill yourself? Do you have a plan?
____
____

If NO, Skip to Question 2.
If YES, ask i.:

i. Do you have the means available to you right now? ______________________________

If NO, STOP! Risk is HIGH, follow action plan below.
If YES, ask ii.

ii. Are you engaging in any behaviors right now to try to harm yourself? Do you want to act on the plan you have right now?
____

If YES to either, risk is IMMEDIATE, follow action plan below.

2. Have you ever attempted suicide before? ____________________________________

If YES, risk is HIGH, follow action plan below.
If NO, risk is MODERATE, follow action plan below.

RISK LEVEL	ACTION
**Low**	1. Give patient resources (1.800.273.TALK or BHR 314.469.6644)
**Moderate**	1. Contract for safety with patient & schedule call back within 4 hours<br>2. Inform Study Psychiatrist (Lenze cell 314-498-1919)<br>3. Call back patient at scheduled time and discuss tx recommendations
**High**	1. Contract for safety with patient & schedule call back within 1 hour or keep patient on phone<br>2. Inform Study Psychiatrist (Lenze cell 314-498-1919)<br>3. Discuss tx recommendations with patient
**Immediate**	1. Keep patient on phone<br>2. Call 911<br>3. Follow instructions of 911<br>4. Inform Study Psychiatrist (Lenze cell 314-498-1919)

© 2026 Abraham J et al. *JAMA Network Open*.

4. Trying to Reach You Letter

Date

Participant Name
Participant Address

Dear Mr./Ms. [ ],

I hope this letter finds you well. It has been approximately [length of time since consent] since you enrolled in the Behavioral Activation and Medication Optimization perioperative study. Our records indicate that we have not been able to successfully reach you for a scheduled phone visit. Please contact me, **[study team member's name and contact info]** as soon as possible. You may also contact **[alternate study team member's name and contact info].**

Thank you for participating in this important study. We look forward to hearing from you again soon.

Sincerely,

[Study team member name]
[Study team member contact info and/or job title]

Washington University School of Medicine

© 2026 Abraham J et al. *JAMA Network Open*.

Center for Perioperative Mental Health

14. Patient materials and handouts

What are Your Wellness Goals?

Having a major surgery comes with mental and physical challenges. It can help to have a plan for your recovery that includes activities to support your well-being. Having a plan for how you spend your time can help you regain a sense of control, help you feel more like yourself, and get you back to doing the things that are important to you. For each activity listed below, consider if it's something you'd like to continue, something you'd like to start doing for the first time, start doing again, or if it's something you don't do or don't want to do during your recovery.

	Continue doing or do more often	Start doing or start doing again	Don't want to do
Spending time with my spouse, friends, or family.			
Mindfulness, meditation, or relaxation techniques.			
Religious or spiritual practices.			
Maintaining my thinking and memory skills.			
Challenging my brain with brain training exercises.			
Improving the amount or quality of my sleep.			
Pursuing a hobby.			
Going out in the community for errands or socializing.			
Doing paid or volunteer work.			
Taking care of the home or yard.			
Exercising or working out.			
Cooking at home.			
Healthy Eating.			

Is there something else that you'd like to do or work towards during your recovery?

© 2026 Abraham J et al. *JAMA Network Open*.

**Planning Your Wellness Activities**<br>Discuss your priorities with your wellness partner. Work together to set specific goals to achieve the things that matter most to you. A wellness activity is something you can do to help yourself feel better mentally or physically.	
Today's Date	
What is your Wellness Activity Goal?<br>(an action focused, specific goal)	
When will you do it?<br>(which days, what time of day?)	
What could get in the way?	
What will help you achieve your goal?	

Tracking the Outcome
Keep track each day what happens with your wellness activities. Make any additional notes you find useful. Discuss with your wellness partner at your next session.

Date	Progress	Notes: How did you feel before, during, after the activity? What made it harder or easier to do?

© 2026 Abraham J et al. *JAMA Network Open*.

Wellness Journal

Week of: ______________

Center for Perioperative Mental Health

Use this form if you'd like to log your daily activities and track your progress on your Wellness Activity Goals.

My Goal This Week is…____

	**Monday**	**Tuesday**	**Wednesday**	**Thursday**
Wellness Activity				
Mood				
Notes				
	**Friday**	**Saturday**	**Sunday**	
Wellness Activity				
Mood				
Notes				

© 2026 Abraham J et al. *JAMA Network Open*.

Mindfulness and Recovery from Surgery
Mindfulness has been defined as the practice of cultivating non-judgmental awareness in everyday life.
Regularly practicing mindfulness can help us to better manage stress. Consider for a moment the stressors you're dealing with right now, including your surgery and medical conditions, as well as any other challenges going on in your life.
Ask yourself:

- Would you like to try new ways to manage stress?
- How has stress impacted your relationships?
- Have you been feeling scattered or disorganized?
- Have you been feeling fuzzy or foggy, instead of clear-headed?
- Do you find it hard to deal with painful emotions?

Setting time aside to practice mindfulness can help as you cope with life's challenges and everyday stressors. It may be difficult at times, but noticing discomfort is part of the journey.
A mindfulness practice consists of formal practices or meditations, as well as informal practices, where you work to bring non-judgmental awareness into your everyday life. It is also helpful to make note of your experiences and reflect on what you are noticing.

Try it out: Mindful Check-In
Adapted from A Mindfulness-Based Stress Reduction Workbook (Stahl & Goldstein 2010)
First, find a place where you can comfortably sit or lie down for a few moments without distractions. Try to bring your full, undivided attention as you read this text, pausing to reflect.
Take a few moments to be still.
Begin by feeling into your body and mind and allowing any thoughts, emotions, or physical sensations to just be.
Allow yourself to simply be here now, in the present moment. Spend some time here, just noticing without judgement, without trying to figure things out.
After a few moments, congratulate yourself for taking time for this meditation practice.

Learn More

1. A Mindfulness-Based Stress Reduction Workbook by Bob Stahl & Elisha Goldstein, 2010 (book)
2. MBSR Every Day: Daily Practices from the Heart of Mindfulness-Based Stress Reduction by Elisha Goldstein & Bob Stahl (book)
3. UCSD Center for Mindfulness Resources: https://cih.ucsd.edu/mindfulness/mindfulness-compassion-resources
4. Healthy Minds Program (mobile app): https://hminnovations.org/meditation-app

© 2026 Abraham J et al. *JAMA Network Open.*

Self-Help for Disrupted Sleep

How has your sleep been lately? If you've had some trouble falling or staying asleep, you're not alone. About 50% of older adults report difficulties with their sleep. Getting enough quality sleep is important to our health and well-being.

Sleep and Surgery

Quite often, our sleep routines get disrupted to some degree around the time of surgery. This can happen for all kinds of different reasons, such as being awakened earlier than usual in the hospital, or sleeping more during the day while you are recovering from surgery and anesthesia. That is normal and typically doesn't persist too long.

If you continue to have problems with your sleep that affect your ability to function in the daytime, consider trying some self-help strategies to try to improve your sleep.

Healthy Sleep Habits

- Aim to stick to a consistent sleep schedule. You may find on some days you need a bit more rest than usual. If you find daytime sleep makes it harder for you to sleep at night, then you should try to reduce or avoid sleeping during the day. Naps that are longer than an hour or that take place late in the afternoon can be especially disruptive to nighttime sleep.
- Build a bedtime routine that helps you to relax and unwind before bed. This could include things like reading, taking a warm bath, or listening to music.
- Eating, drinking, and smoking can all affect your sleep*:*
    - Eating large meals, spicy foods, or fatty foods can lead to wakefulness or discomfort at bedtime. Try to finish eating for the day 2-3 hours before bedtime. If you do need to eat before bed, choose something small and light.
    - Caffeine can take as long as 8 hours to fully wear off.
    - Nicotine is a stimulant and can make it hard to sleep.
    - Alcohol may seem like it helps you to nod off, but it keeps you from getting deep sleep. Then, when it wears off, you may wake up in the middle of the night.
- Consider your sleeping environment: is it dark, quiet, cool, and comfortable? You may find you need some additional pillows to be comfortable after surgery. Are there things in your bedroom that could make sleep more difficult, like a TV or computer?
- If you find that you can't sleep after trying for a while, get up and go to another room to do something relaxing until you feel sleepy. The reason for this is that it helps keep a strong link in the brain between being in bed and sleeping. Spending too much time in bed awake weakens that link – it sends our brain the wrong message about what it means to be in bed.
- Talk to your doctor if you continue to have difficulty sleeping, are sleeping excessively, or are not feeling rested. Your primary care doctor may be able to help you, or they may refer you to a sleep specialist.

Questions to ask yourself:

1. What do your bedtime and waking routines look like? Have they changed since your surgery?
2. Are your sleep habits working for you or are there some things you'd like to change?

Next Steps

One of the most common first steps for treating sleep problems is to keep a sleep diary. This doesn't have to be complicated, and it will likely just take a moment each day. Here's a few things you may want to include in your sleep diary:

- when you went to bed
- about how long it took you to fall asleep
- how many times you woke up during the night, and for how long
- when you woke up
- when you got out of bed
- estimated total nighttime sleep
- estimated time in bed
- any daytime sleep

Keeping a sleep diary is a good way to understand patterns and can help you to see if any changes you make are working. It can also help you to quantify the problems you are having should you need to discuss your sleep with your doctor.

Learn More

1. Society of Behavioral Sleep Medicine Public Resources: offers expert videos on sleep topics https://www.behavioralsleep.org/index.php/sbsm/community-education

© 2026 Abraham J et al. *JAMA Network Open.*

2. National Health Service (UK) Sleep problems and insomnia self-help guide: this guide can be accessed online or downloaded and printed out. Note that the additional resources in the hyperlinks are to services offered in the UK.
https://www.nhsinform.scot/illnesses-and-conditions/mental-health/mental-health-self-help-guides/sleep-problems-and-insomnia-self-help-guide
3. CBT-I Coach: This mobile phone app is available for Apple and Android devices. It was designed by the Veterans Administration as a component to receiving an evidence-based psychotherapy for treating insomnia, however it can be used on its own.
https://www.ptsd.va.gov/appvid/mobile/cbticoach_app_public.asp
4. Washington University Sleep Medicine Center offers evaluation and treatment for sleep disorders.
https://sleep.wustl.edu/ 314-362-4342

© 2026 Abraham J et al. *JAMA Network Open.*

Training Your Brain

You probably know that exercise helps keep your muscles strong, but "use it or lose it" applies to your brain as well. Living a life that is mentally stimulating can provide protection against memory disorders and brain decline. Reading books, solving problems, learning new information and skills, hobbies that require concentration, and playing interactive games are all activities that contribute to brain health.

On the cellular level, brain cells that are being stimulated: receive more oxygen, create more connections to other cells, and help to develop new brain cells. Over time, this keeps your brain healthy and sharp. On the other hand, in an environment without enough mental stimulation, the brain becomes less healthy over time.

Lifestyle changes such as retirement, or a prolonged medical recovery period, can lead to a reduction in mental stimulation. It's worth doing some reflecting about which activities in your life are mentally stimulating, and whether they will continue to be available to you after your surgery. If not, consider trying out some new activities to keep your brain in shape.

Try it out: Happy Neuron

CPMH participants can use a brain training program called Happy Neuron during their participation free of charge. This is a web-based program with different games designed to challenge different aspects of your memory and thinking. Each game has several levels that get progressively harder so that you continue to be challenged.

Read about the program here: https://www.happy-neuron.com/

If you'd like to give it a try, ask your Wellness Partner to set up your account.

Try it out

Keeping your brain healthy doesn't have to be boring – in fact, it really shouldn't be. Follow your interests and curiosities to activities that you enjoy or that help you feel a sense of accomplishment or purpose. Seek to go deeper and challenge yourself in things that interest you, and seek out experiences that are new to you. The options are truly endless, from arts and crafts, to crosswords, jigsaw puzzles, board games, trivia games, reading, or listening to audio books or podcasts. If you are feeling bored, take it as a sign that your brain could use some more exercise.

1. Oasis: Oasis is an organization with 9 centers in the US including St. Louis. They offer lifelong learning classes, health and wellness classes, and volunteer opportunities. They have in-person classes as well as online classes. https://www.oasisnet.org/ 314-862-2933

2. Your local library: Many libraries offer access to materials digitally. This is a great way to keep your mind busy even when you're staying at home to recover. A librarian can help make sure you're set up and ready to read (or listen, if you prefer audio books).

Activity – Rest Cycle
Have you ever had a “good” day with your pain or energy where you pushed yourself to be really active, only to pay for it with increased pain and fatigue the next day (or days!)? This is a common experience for people who are recovering from surgery, or who are coping with chronic pain or medical conditions. This experience is called the **pain cycle**, and sometime people can find themselves repeating the pain cycle over and over.

Pain Cycle

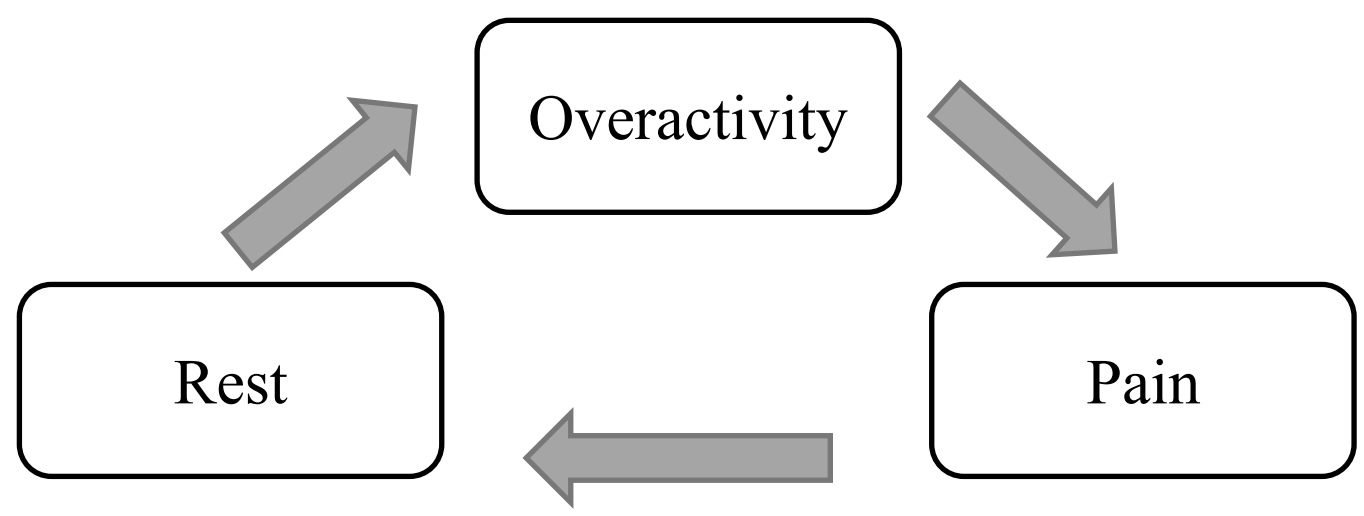

Repeating the pain cycle over and over again can cause problems in the short term and the long term. In the short term, it can be difficult to cope with the increased pain and fatigue, and in some cases can even hinder your body’s recovery from surgery. It can have an emotional impact as well: you may feel discouraged, down, tense, or worried. In the long term, you may find yourself avoiding things that usually make you feel good in order to prevent pain. What can you do instead? One approach is to develop an **activity – rest cycle** instead, or in other words, pacing yourself. You can alternate periods of planned rest between periods of planned activities.

Activity – Rest Cycle

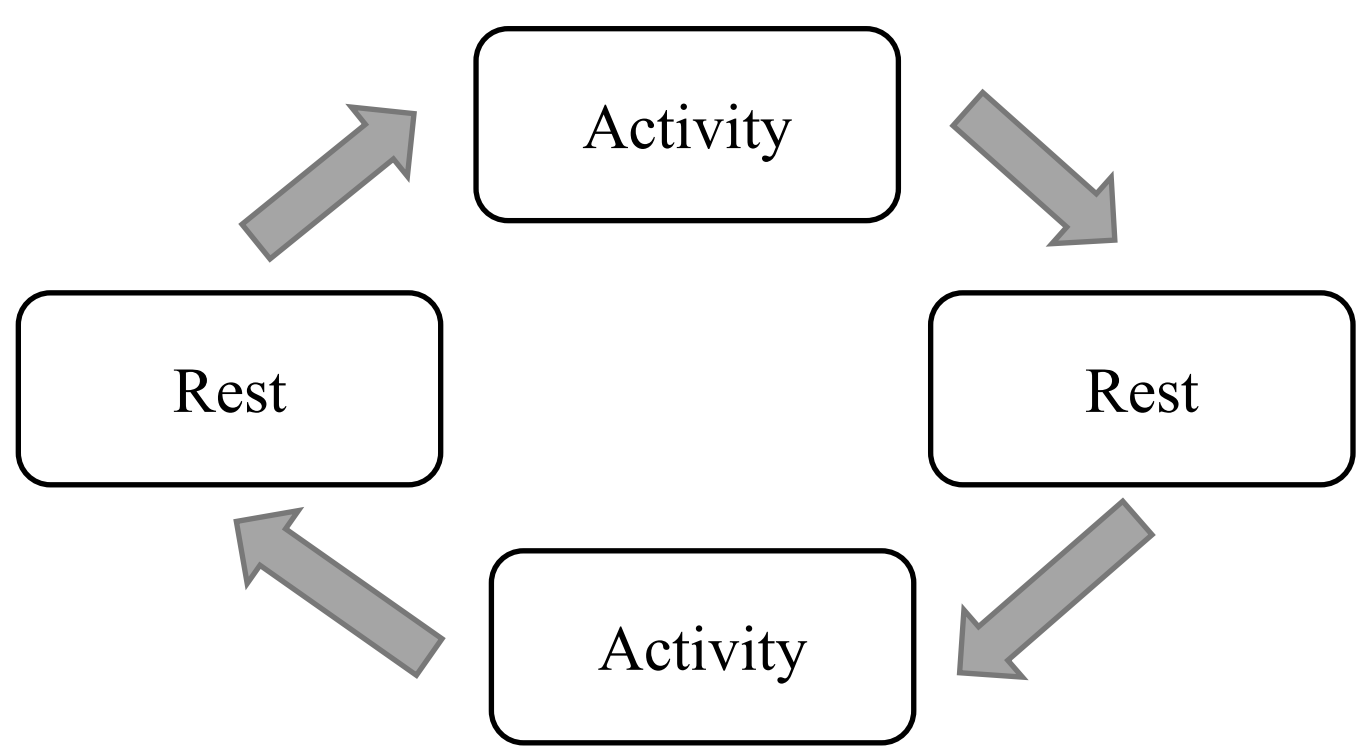

Questions to ask yourself:

1. What kinds of things do you tend to overdo?

2. What might be a good time limit for each activity before you stop and rest?

3. How long might you need to rest afterwards?

Then, keep track of how it goes and adjust from there. You can try using the chart below or keep track on a calendar or journal. Make a quick note of how you feel afterwards.

**Activity Pacing Form**			
	Activity 1	Activity 2	Activity 3
Activity			
Active Goal			
Rest Goal			
Date:			
Active:			
Rest:			
# times:			
Notes:			
Date:			
Active:			
Rest:			
# times:			
Notes:			
Date:			
Active:			
Rest:			
# times:			
Notes:			
Date:			
Active:			
Rest:			
# times:			
Notes:			

Center for Perioperative Mental Health (CPMH) Pharmacological (or Medication) Optimization Standard Operating Procedure (SOP)

1. OVERVIEW

Medication optimization aims to a) reduce the use of problematic medications that increase risk for falls and delirium in older adults b) increase sub-therapeutic doses of antidepressant medications. Medication optimization during the perioperative period also entails assessing for these specific medications throughout the hospital stay in order to ensure harmful medications are not initiated and that patients are appropriately re-started on their antidepressant medications. Medication optimization occurs through a collaborative decision-making process throughout the perioperative period.

2. Home Medication Optimization

3. Session 1 (May occur prior to or after hospital admission)

1. Before session 1:

- The Wellness Partner will arrange a phone call with the patient and a pharmacy team member.
- The pharmacy team member will collect and review the patient's past medical history, home medication list and medication fill history (when available) from the electronic health record (EPIC) and prepare for a focused discussion of targeted antidepressant and harmful medications found in Appendix 1 and Appendix 2. The pharmacy team member will provide a draft written summary and suggested plan to the CPMH intervention team for all patients randomized to the intervention arm. The summary email should include the following:
    - Patient ID, surgery date, surgery/procedure type, medication list, assessment, and a proposed recommendation (for patients with no targeted interventions, the recommendation will note that there are no changes to be made).
- If a pharmacy team member is not available, the Wellness Partner can perform this task in collaboration with the pharmacy team.

2. During session 1:

- The Wellness Partner will orient the patient to the Wellness Program and introduce the pharmacy team member.
- The pharmacy team member will then let the patient know what the purpose of their discussion is:
    1. Example: "Many older adults are taking multiple medications. Some medications can cause problems such as increased risk for falls or confusion for older adults, especially around the time of surgery. We would like the review the list of your home medications and discuss any specific medications that could be optimized before your surgery that pertain to your brain."
- Confirm and review the medications, doses and frequencies on the patient's home medication list. Ask for any over-the-counter medications or supplements, as well as any as-needed medications.
- If the patient isn't taking any medications eligible for medication optimization, let them know you didn't identify any medications that could cause problems for their thinking or balance. Let them know that our team will be reviewing the medications that they are given in the hospital and once they return home as well.
- In the event that patients ask questions to the pharmacy team or Wellness Partner that are not specific to the targeted medications identified for optimization in this study, the intervention team will inform the patient to contact their physician. If the patient needs help doing this, the intervention team can facilitate connecting the patient with the applicable prescriber. If the patient is taking harmful medication eligible for **deprescribing, dose reduction or frequency change**:
    1. Find out the reason they take it and whether they perceive it to be helpful.
    2. Find out how long they have been taking it and if they have ever been on a different dosage.
    3. Ask if they've noticed any problems with balance, cognition, sedation or confusion.
    4. If the patient has been taking a centrally acting anticholinergic ask if they have been experiencing any possible anticholinergic side effects from the harmful medication such as dry mouth or constipation.

© 2026 Abraham J et al. *JAMA Network Open*.

5. Assess the patient's willingness to a) stop taking the medication, b) take a lower dose of the medication, or c) take the medication less frequently.
6. Find out who prescribes the medication and get buy-in to contact the prescriber if indicated (e.g., patient in agreement to stop taking or reduce the dose of a prescribed medication or if a potential, non-harmful prescription alternative exists).
7. For sleep medications specifically:
    - The primary focus should be on counseling and sleep hygiene practices without the use of pharmacologic sleep aids.
    - If sleep medications are needed, melatonin, trazodone, and ramelteon are considered relatively safe.
    - If the patient expresses major concern about their ability to sleep if the harmful medication is discontinued, over the counter melatonin at a single daily dose of 3-5mg can be suggested as an alternative.
    - If the patient has failed melatonin and is prescribed a harmful sleep medication eligible for deprescribing, the pharmacy team may contact the PCP and recommend changing the patient's prescription to one of the safer sleep medications.

- If the patient is taking an antidepressant eligible for **dose escalation**:
    1. Find out the reason they take it and whether they perceive it to be helpful.
    2. Explain that their medication may be more helpful for improving their mood and anxiety if taken at a higher dose.
    3. Ask if they have ever tried taking it at a higher dose and whether they had any side effects or improvements in their depressive symptoms.
    4. Ask if they would have any concerns with increasing the dose.
    5. Find out who prescribes the medication and get buy-in to contact the patient's prescriber.
- Ask the participant whether they are agreeable to receiving separate phone calls from the Wellness Partner and pharmacy team performing MO at the participant's preferred contact times for the rest of the study.
- Summarize the plan and next steps, making sure to address any questions or concerns that the patient has.
    - For most changes, the next step would be confirming the plan with the intervention team.
    - Let the patient know when they can expect to hear back from you.
    - Consider the feasibility of any changes in the context of patient's surgery timeline.

3. After session 1:

- Provide a revised (or new if this did not occur before the call) written summary to the CPMH Intervention team of the intervention call.
- When necessary, contact the patient's PCP/ prescriber. In some cases, the plan may be discussed first with the patient and then with their provider, before finally following up with the patient.
    - Discontinuing an over-the-counter medication or temporarily reducing the frequency of PRN medications can be enacted without contacting the PCP/ prescriber. For other changes, the PCP/ prescriber should be contacted.
    - Typically, this will occur via Epic. For providers outside of WU/BJC Epic, fax a letter and call the office to confirm receipt and/or discuss with the provider.
- Call the patient back in the agreed upon time frame to discuss the plan and next steps:
    - Update the patient on any action that the pharmacy team has taken since the last contact (for example, if any correspondence occurred with their prescribing physician).
    - For deprescribing, indicate which medications to stop, how to stop (immediately vs taper), and any contingencies which could include alternative therapies (e.g., "if patient has worsening bladder symptoms, we will recommend a different non-toxic medication").
    - For dose escalation, indicate which medication to increase, how to increase (increment and maximum), and any contingencies.
    - Decide on a plan for communicating with their PCP/prescriber (see Contacting Medical Providers below)
    - Note any concerns or barriers expressed by the patient and update the CPMH Intervention team.
- Record the patient's relevant medications and intervention information in REDCap according to the medication data collection form.

- Record in REDCap on the “patient information” form whether the participant agrees to be contacted separately by the pharmacy team.

4. Medication Optimization During Hospital Admission

1. Upon Admission

- Review the home medication list that is entered into EPIC and ensure it matches what was discussed with the patient if a previous session has already occurred.
- Check the active orders tab and medications administered daily in EPIC to assess any previous or new orders for potentially harmful medications. Enter any relevant meds into REDCap ordered or administered within one week prior to surgery date if the patient has been hospitalized for longer than one week prior to surgery. After surgery occurs, all relevant hospital medication orders and administration records should be collected until discharge.
- Check if the patient’s home antidepressant (or alternative or new antidepressant) was started.
- In the event that an antidepressant is not started upon admission or if a previous or new potentially harmful medication is started, a CPMH pharmacist will be notified and will contact the primary care team to discuss an intervention
- Enter all relevant interventions in REDCap

2. Transition of care & Post-operative Check for Resuming Antidepressant Treatment

- Check the patient’s active orders tab and medications administered daily after each transition of care and within 24 hours post-operatively to assess whether the patient’s antidepressant treatment was held for surgery. If it was held, check to see if it has been re-started.
- In the event that a held antidepressant is not re-started post-operatively, a CPMH pharmacist will be notified and will contact the primary care team to discuss restarting.
- Enter all relevant interventions in REDCap

3. Transition of care & Post-operative Check for potentially harmful medications

- Check the patient’s active orders tab and medications administered daily after each transition of care and within 24 hours post-operatively to assess whether a potentially harmful medication has been started.
- In the event that a new or previous potentially harmful medication is initiated, a CPMH pharmacist will be notified and will contact the primary care team to discuss discontinuing, decreasing or alternative therapy.
- Enter all relevant interventions in REDCap

5. Medication Reconciliation and Optimization AT Hospital Discharge

- At the time of hospital discharge, the pharmacy team will review the discharge medication list from the discharge summary note and the fill history for antidepressants and potentially harmful medications.
- In the event that something notable is identified, the pharmacy team will convey this information to the Wellness Partner and they will discuss this with the patient at the next planned session.
- The pharmacy team will record relevant medications and interventions in REDCap.

6. Sessions occurring after discharge

- Check REDCap patient information form to confirm whether the participant is agreeable to separate calls from the Wellness Partner and pharmacy team.
- Throughout the 3-month post-operative period, the Wellness Partner and pharmacy team will continue to follow up about the medication optimization plan to assess whether it was

© 2026 Abraham J et al. *JAMA Network Open*.

implemented, problem solve any barriers, and assess for any benefits or adverse effects. The Wellness Partner will provide regular updates to the pharmacy team.

- If session 1 did not occur before hospitalization, this will occur after discharge.
- In the event that patients ask questions to the pharmacy team or Wellness Partner that are not specific to the targeted medications identified for optimization in this study, the intervention team will inform the patient to contact their physician. If the patient needs help doing this, the intervention team can facilitate looking up contact information or identifying an upcoming appointment.

1. *Targeted Harmful Medications for Optimization*

Included on the list are medications that may be harmful for older adults and are therefore eligible for deprescribing. CNS-active potentially inappropriate medications (PIMs) that we targeted for this study were based on the 2023 AGS Beers Criteria [1], focusing on centrally acting drugs with strong anticholinergic or sedative effects. Selected classes included first-generation antihistamines, antiemetics, antimuscarinics, antiparkinsonian agents, antispasmodics, muscle relaxants, benzodiazepines, non-benzodiazepine "Z drugs," and tricyclic antidepressants (see table below). These medications can cause decreased energy, cognitive impairment, and increased fall risk. These are medications that do not align with larger goals of care and for which risks outweigh benefits. Prioritize drugs for discontinuation that have the lowest benefit-harm ratio and lowest likelihood of adverse withdrawal reactions or disease rebound syndromes (or if so, can they be tapered).

**Targeted Harmful Medications for Optimization**	
**Generic Name**	**Common Brand names and OTC names**
**Anticholinergics: First Generation Antihistamines:** Highly anticholinergic; clearance reduced with advanced age, and tolerance develops when used as hypnotic; risk of confusion, dry mouth, constipation, and other anticholinergic effects or toxicity	
Brompheniramine	Bromfed DM, Dimetapp, Brotapp [OTC] [DSC]; Lodrane D [OTC]; Q-Tapp Cold & Allergy [OTC] [DSC]; Rynex PSE [OTC], Brohist D [OTC]
Carbinoxamine	Karbinal ER, RyVent
Chlorpheniramine	Aller-Chlor [OTC]; Allergy Relief [OTC]; Allergy [OTC]; Allergy-Time [OTC] [DSC]; Chlor-Trimeton Allergy [OTC]; Chlor-Trimeton [OTC]; Pharbechlor [OTC]
Clemastine	
Cyproheptadine	Periactin
Dexbrompheniramine	Ala-Hist PE [OTC]; Alahist PE [OTC]; G-Hist PE [OTC], Chlo Hist [OTC], Chlo Tuss [OTC]
Dexchlorpheniramine	RyClora, Rescon [OTC], WesTussin DM [OTC]
Dimenhydrinate	Driminate [OTC]
Diphenhydramine (oral)	Aler-Dryl [OTC]; Allergy Relief [OTC]; Anti-Hist Allergy [OTC]; [DSC]; Banophen [OTC]; Benadryl Allergy Extra Str [OTC]; Benadryl Allergy Ultratabs [OTC]; Benadryl Allergy [OTC]; Complete Allergy Relief [OTC]; Diphen; Diphen [OTC]; Diphenhist [OTC]; Geri-Dryl [OTC]; GoodSense Allergy Relief [OTC]; GoodSense Sleep Aid [OTC]; M-Dryl [OTC]; Naramin [OTC]; Nighttime Sleep Aid [OTC]; Pharbedryl [OTC]; Siladryl Allergy [OTC]; Simply Sleep [OTC]; Sleep Tabs [OTC]; Tetra-Formula Nighttime Sleep [OTC]; Total Allergy Medicine [OTC]; Total Allergy [OTC]; ZzzQuil [OTC]
Doxylamine	Sleep Aid [OTC], Unisom, Diclegis, Bonjesta
Hydroxyzine	Vistaril
Meclizine	Antivert; Bonine [OTC]; Motion-Time [OTC]; Travel-Ease [OTC]
Pyrilamine	Capron DM [OTC]; Capron DMT [OTC], Glen PE [OTC], Midol Complete [OTC]
Triprolidine	Histex PD [OTC]; Histex PDX [OTC] [DSC]; Histex [OTC]
**Anticholinergics: Antiemetics**	
Prochlorperazine	Compro; Compazine
Promethazine	Phenergan; Promethegan
**Anticholinergics: Antimuscarinics for urinary incontinence**	

© 2026 Abraham J et al. *JAMA Network Open.*

Fesoterodine	Toviaz
Oxybutynin	Ditropan
Tolterodine	Detrol; Detrol LA
**Anticholinergics: Antiparkinsonian agents:** Not recommended for prevention or treatment of extrapyramidal symptoms with antipsychotics; more effective agents available for treatment of Parkinson disease	
Benztropine (oral)	Cogentin
Trihexyphenidyl	Artane; Trihexy
**Anticholinergics: Antispasmodics:** Highly anticholinergic, uncertain effectiveness	
Atropine (excludes ophthalmic)	Lomotil
Belladonna alkaloids	B&O Supprettes; B&O; Opium and Belladonna
Clidinium-chlordiazepoxide	Librax
Dicyclomine	Bentayl
Hyoscyamine	Anaspaz; Ed-Spaz; Hyosyne; Levbid; Levsin; Levsin/SL; NuLev; Oscimin, Symax Duotab; Symax-SL; Symax-SR
Methscopolamine	
Propantheline	
Scopolamine	Transderm-Scop
**Anticholinergics: Skeletal muscle relaxants:** Most muscle relaxants poorly tolerated by older adults because some have anticholinergic adverse effects, sedation, increased risk of fractures; effectiveness at dosages tolerated by older adults questionable	
Carisoprodol	Soma; Vanadom
Chlorzoxazone	Lorzone
Cyclobenzaprine	Amrix; Fexmid; Flexeril
Metaxalone	Skelaxin; Metaxall
Methocarbamol	Robaxin
Orphenadrine	
**CNS: Benzodiazepines:** Older adults have increased sensitivity to benzodiazepines and decreased metabolism of long-acting agents; in general, all benzodiazepines increase risk of cognitive impairment, delirium, falls, fractures, and motor vehicle crashes in older adults. May be appropriate for seizure disorders, rapid eye movement sleep behavior disorder, benzodiazepine withdrawal, ethanol withdrawal, severe generalized anxiety disorder, and periprocedural anesthesia	
Alprazolam	Xanax
Chlordiazepoxide	Librium
Clonazepam	Klonipin
Clorazepate	Tranxene-T
Diazepam	Valium
Estazolam	ProSom
Flurazepam	
Lorazepam	Ativan
Midazolam	Nayzilam
Oxazepam	Serax
Quazepam	Doral
Temazepam	Restoril
Triazolam	Halcion
**CNS: Non-benzodiazepine "Z drugs":** Nonbenzodiazepine benzodiazepine receptor agonist hypnotics (ie, Z drugs) have adverse events similar to those of benzodiazepines in older adults (eg, delirium, falls, fractures); increased emergency room visits/ hospitalizations; motor vehicle crashes; minimal improvement in sleep latency and duration	
Eszopiclone	Lunesta
Zaleplon	Sonata
Zolpidem	Ambien; Ambien CR; Edluar
**CNS: other:** High rate of physical dependence; sedating	
Meprobamate	Equanil
**CNS: Tricyclic Antidepressants:** Highly anticholinergic, sedating, and cause orthostatic	

© 2026 Abraham J et al. *JAMA Network Open.*

hypotension; safety profile of low-dose doxepin (≤6 mg/day) comparable to that of placebo	
Amitriptyline	Elavil
Amoxapine	Asendin
Clomipramine	Anafranil
Desipramine	Norpramin
Doxepin > 6 mg/day	Silenor
Imipramine	Janimine; Tofranil-PM; Tofranil
Nortriptyline	Pamelor
Protriptyline	Vivactil
Trimipramine	Surmontil
Secondary amine tricyclics (desipramine, nortriptyline, and protriptyline) on the list are generally less anticholinergic/antihistaminergic. They may not always require an intervention. These will be important to specifically discuss with the intervention team.	

2. Antidepressant Medications of Interest

Included in this list are relevant antidepressants that should be collected. Within this document, a list of eligible antidepressants for dose escalation are noted. We targeted the following antidepressants for dose optimization: SSRIs, SNRIs, MAOIs, bupropion, mirtazapine, and vilazodone (see table below). Minimum effective doses were based on the Antidepressant Treatment History Form: Short Form (ATHF-SF), consistent with prior research and expert guidelines [2-5].

**Antidepressant medications of interest**	
Generic	US Brand Name
SSRIs	
**Citalopram**	**Celexa**
**Escitalopram**	**Lexapro**
Fluvoxamine	Luvox
**Fluoxetine**	**Prozac, Sarafem**
**Paroxetine**	**Paxil, Paxil CR, Brisdelle, Pexeva**
**Sertraline**	**Zoloft**
Vortioxetine	Tintellix
SNRIs	
Desvenlafaxine	Pristiq, Khedezla
**Duloxetine**	**Cymbalta, Drizalma sprinkle**
Levomilnacipran	Fetzima
**Venlafaxine**	**Effexor, Effexor XR**
Milnacipran	Savella
MAOIs	
Isocarboxazid	Marplan
Moclobemide	
Phenelzine	Nardil
Selegiline	Eldepryl, Zelapar, Emsam
Tranylcypromine	Parnate
Other	
**Bupropion**	**Wellbutrin, Aplenzin, Forfivo**

Mirtazapine	Remeron
**Vilazodone**	**Viibryd**

BOLDED meds are those eligible for dose escalation

- Citalopram: increase to 20mg if dose less than 20mg.
- Escitalopram: increase to 10mg if dose less than 10mg.
- Sertraline: increase to 50mg if dose less than 50mg.
- Fluoxetine: increase to 20mg if dose less than 20mg.
- Paroxetine: increase to 20mg if dose less than 20mg.
- Duloxetine: increase to 60mg if dose less than 60mg.
- Venlafaxine: increase to 150mg if dose less than 150mg.
- Vilazodone: titrate (by 10mg/week) to 40mg if dose less than 40mg.
- Bupropion: increase total daily dose to 300mg if total daily dose is less than 300mg.

7. Sample Communications with Patients

1. De-Prescribing

"Oxybutynin causes fatigue and cognitive problems in older people. Have you noticed any problems with your concentration, energy, or balance?" "You told me you take it for your bladder—do you think it's helping?" "You will think more clearly, have more energy, and have better balance if you stop it or switch to a different medication that isn't bad for your brain." "What do you think? [if in doubt, ask directly: "would you be willing to stop or switch this medication?]" . Is it ok with you if we contact your doctor to discuss these changes?"

2. Dose Escalation

"I see that you are taking 75mg of venlafaxine each day, and continue to have anxiety and a low mood. Venlafaxine works better to manage those symptoms when it is taken at a higher dose, 150mg. Have you ever taken venlafaxine at a dose higher than 75mg? Would you have any concerns with trying a higher dose to provide better management of your mood and anxiety? Is it ok with you if we contact your doctor to discuss these changes?"

3. Following up

"When you were in the hospital, you stopped taking oxybutynin because it causes fatigue and worsens memory. Since then, have you noticed any improvement in your energy or thinking?"

(If person responds yes, then make sure to respond positively and link this to their goals, eg "Great – your energy is improved! You're closer to your goal of taking walks with your wife in the evening!"

If the person responds no, then still say: "That's ok – people don't always notice these improvements. But I can guarantee you, since you stopped this medication, your brain is working better.")

8. Contacting Medical Providers

The prescriber should be contacted if there is a recommended change to the prescription that the patient would like to implement. The patient can choose whether they would like to contact their prescriber themselves or if the pharmacy team can contact them directly. We prefer that the pharmacy team contact the prescriber.
In both cases, the formal letter included in the MO SOP should be used. Based on patient preference, the pharmacy team can send directly to prescriber or provide it to the patient to give to their prescriber.
We do not need to contact the prescriber in the case of a recommendation to change PRN med usage (e.g., reduction in frequency of use) or if it is an OTC med (unless the patient prefers the prescriber be notified in these cases).
Timeline: contact prescriber prior to the surgery (if MO occurs prior to surgery), ideally within 48 hours

© 2026 Abraham J et al. *JAMA Network Open.*

1. Sample Letter to Physician

Washington University in St.Louis

School of Medicine

MONTH, DAY, YEAR

Dear Dr. XXX,

Your patient, XXX, is participating in a clinical trial through the Center for Perioperative Mental Health.

The goals of this trial are to mitigate anxiety and depression symptoms during the perioperative period. Key CPMH precepts include:

* Patient preparedness for surgery in the preoperative period
* Enhancing recovery during the postoperative period, including coordination and communication with inpatient providers

As part of these goals, we reviewed the medications your patient is taking, and we recommend stopping / tapering off of/ reducing frequency of / reducing dose of these drugs:

(Medication name, dose, frequency, reason we have suggested stopping)

We have identified that your patient is taking a subtherapeutic dose of their antidepressant, and we recommend increasing the dose as follows:
Current: Medication name, dose, frequency
Recommended: Medication name, dose, frequency
****

Thank you very much for your consideration. We will be able to track patients carefully as they proceed through the perioperative period and <u>will be happy to work with you in providing monitoring and feedback.</u>

We believe our research will achieve significant improvements in perioperative mental health and overall patient care.

Sincerely,

Eric Lenze, MD
Professor of Psychiatry

Perioperative Mental Health Intervention Training

Training was provided for all interventionists: prior to beginning the study, wellness partners participated in four 1-hour weekly training sessions covering the psychological management's manual of procedures and principles of compassionate care; and pharmacy team members were trained through multiple weekly sessions to review good clinical practices, the pharmacological component's manual of procedures, EHR access and navigation, and principles of compassionate care.

eMethods 2. Perioperative mental health intervention implementation evaluation methods: data collection and analysis

eTable 1. Intervention delivery process measures

PMH bundle delivery process measures	Data Source	Description	Time Points
Intervention implementation fidelity	Fidelity rating checklists within weekly intervention session forms based on a framework developed by Bellg et al. [6] and Borrelli et al. [7]	Data collected via checklist on PMH bundle component enactment and interventionist adherence to manual of procedures, alongside patient reception of psychological and pharmacological components	Weekly; EOS
Patient experiences with intervention	Patient interviews informed by the Consolidated Framework of Implementation Research, comprising of 39 constructs across five domains [8]: intervention characteristics, inner setting, outer setting, individual characteristics, and implementation process	Interviews gathering patient perspectives on their experiences with the intervention and its delivery	EOS
Interventionist experiences	Interventionist periodic reflections [9]	Meetings to capture interventionist (psychological management wellness partner and MO pharmacy team) experiences with patients and intervention, as well as adaptations to intervention	Start of study; mid-point; EOS
	Interventionist case review notes		Weekly
Intervention delivery	Patient intervention session forms	Documentation of the total number of psychological management sessions, the timing of delivery (preoperative and postoperative), the modality (phone or zoom), and the duration of each session in minutes.	Weekly
Target engagement	Patient self-reported combined measure of modified versions of: Sinclair Compassion Questionnaire-Short Form (SCQ-SF) [10], Behavioral Activation for Depression Scale (BADS) Activation subscale [11], the MiPrep survey [12], and single-item question about "whether speaking with a pharmacist helped patients to understand that certain medications can affect mental health?"	The SCQ-SF is a 5-item patient-reported measure modified to assess compassion of the wellness provider; the BADS Activation Subscale (7 items) measures target engagement of behavioral activation in studies of geriatric depression [13]; and the MiPrep survey (3 items) assesses patients' preparation for medical interventions	2 months postoperatively

Data Analysis of Intervention Implementation Outcomes

Intervention implementation fidelity: Separate checklists were developed for psychological and pharmacological components, based on guided principles of fidelity [6,7]. 20% of participants were randomly selected from each surgical cohort for fidelity evaluation (cardiac, orthopedic, and oncologic; n = 60), with even distribution across interventionists. Participants were eligible for intervention implementation fidelity evaluation if they had completed at least four intervention sessions, and fidelity was assessed weekly and at end of study. Fidelity to psychological management was assessed by a trained rater (RM), while fidelity to the pharmacological component was assessed by a medical resident (EW) and a research coordinator (EL) for the 60 participants.

Patient and interventionist experiences with intervention: Patient interviews informed by the Consolidated Framework of Implementation Research [8] were conducted at end of study to gather patient perspectives on their experiences with the intervention and its delivery. Interventionist (psychological management wellness partner and MO pharmacy team) experiences were captured using interventionist periodic reflections [9] at the start, mid-point, and end of study, and notes transcribed from weekly interventionist case reviews.

Qualitative data was analyzed using inductive thematic analysis and content analysis [14,15]. First, a researcher and medical resident (JW, JA) read through several transcripts (e.g., focus groups, interviews) multiple times for familiarity. Second, transcripts were openly coded using data-driven codes. Researchers independently and iteratively coded data and created a codebook for the remaining transcripts following multiple rounds of discussion for 100% consensus. Third, similar and overlapping codes were organized into sub-themes and higher-level themes.

Patient intervention session forms were analyzed using content analysis. A researcher (JA) and research coordinator (AM) first organized and read through session forms to familiarize with the data. Second, forms were openly coded using phrases relevant to session content. Similarly, a codebook was iteratively refined and finalized following multiple rounds of discussion between JA and AM for 100% consensus. Third, the frequency of each code was tabulated and presented categorically.

Intervention delivery: Intervention delivery was measured with weekly patient intervention session forms. Data points included the total number of psychological management sessions for each patient, along with timing of delivery (preoperative and postoperative), modality (phone or zoom), and duration of each session in minutes.

Target engagement: We administered a survey two months post-surgery to patients in the intervention group who completed at least 1 session. This patient self-reported survey was designed to assess participant engagement with key components of the intervention, including behavioral activation, perceived compassion, surgical preparedness, and education on medication. The survey consisted of the modified Sinclair Compassion Questionnaire-Short Form (SCQ-SF, 5 items) [10], Behavioral Activation for Depression Scale (BADS, 7 items) Activation subscale [11], the MiPrep survey (3 items) [12], and single-item question about "whether speaking with a pharmacist helped patients to understand that certain medications can affect mental health?" All survey items were rated on a 5-point Likert scale ranging from "strongly disagree" (1) to "strongly agree" (5). Results were analyzed descriptively by cohort.

© 2026 Abraham J et al. *JAMA Network Open*.

eTable 2. Cronbach alphas at baseline and 3 months for each surgical sub-population for depression (PHQ-9) and anxiety (GAD-7) scales.

	Baseline				Month 3			
	Cardiac	Oncologic	Orthopedic	Pooled	Cardiac	Oncologic	Orthopedic	Pooled
Anxiety Symptoms (GAD-7)	0.66	0.75	0.71	0.71	0.85	0.86	0.78	0.84
Depressive Symptoms (PHQ-9)	0.64	0.73	0.64	0.67	0.79	0.77	0.79	0.78

© 2026 Abraham J et al. *JAMA Network Open.*

eTable 3. Impact of intervention on health-related quality of life

Least Squares Means (Standard Errors)[a]	Cardiac		Oncologic		Orthopedic		Pooled	
	Intervention	EUC	Intervention	EUC	Intervention	EUC	Intervention	EUC
**# of Healthy Days**								
**Month 0**	14.9 (1.0)	15.5 (1.1)	16.5 (1.0)	15.8 (1.1)	15.6 (0.9)	13.4 (1.0)	15.6 (0.5)	14.8 (0.6)
**Month 1**	17.0 (1.1)	17.2 (1.1)	17.4 (1.0)	16.4 (1.1)	17.0 (0.9)	14.9 (1.0)	17.1 (0.6)	16.1 (0.6)
**Month 3**	21.7 (1.1)	19.5 (1.1)	21.8 (1.0)	18.3 (1.1)	20.6 (0.9)	19.8 (1.0)	21.3 (0.6)	19.2 (0.6)
**Difference Between Months 0 and 3**	6.9 (1.1)	4.0 (1.1)	5.3 (0.8)	2.5 (0.9)	5.1 (1.0)	6.4 (1.0)	5.7 (0.6)	4.5 (0.6)
**Contrast: LSM (SE); P-value**	-2.8 (1.5); p = 0.06[b]		-2.8 (1.2); p = 0.02[b]		1.3 (1.4); p = 0.3[b]		-1.3 (0.8); p = 0.1[b]	

[a]The pooled analysis reflects the average across cohorts and should be distinguished from the meta-analysis.
[b]The contrast of interest is (INT_{M0} – INT_{M3}) - (UC_{M0} – UC_{M3}).

© 2026 Abraham J et al. *JAMA Network Open*.

eTable 4. Incidences of delirium and all-cause rehospitalizations

	Cardiac (n = 102)		Oncology (n = 100)		Orthopedics (n = 104)		Pooled (N = 306)	
	Intervention (n = 51)	EUC (n = 51)	Intervention (n = 52)	EUC (n = 48)	Intervention (n = 50)	EUC (n = 54)	Intervention (n=153)	EUC (n=153)
**Delirium, n (%)**	17 (33)	17 (33)	2 (4)	4 (8)	1 (2)	1 (2)	20 (13)	22 (14)
**Rehospitalizations, n (%)**	7 (14)	13 (25)	5 (10)	6 (13)	4 (8)	6 (11)	16 (10)	25 (16)

© 2026 Abraham J et al. *JAMA Network Open.*

eTable 5. Impact of intervention on pain

LSMs (Standard Errors)	Cardiac			Oncologic			Orthopedic			Pooled		
	Intervention	EUC	Difference between Intervention and EUC	Intervention	EUC	Difference between Intervention and EUC	Intervention	EUC	Difference between Intervention and EUC	Intervention	EUC	Difference between Intervention and EUC
**Pain Interference**												
Month 1	1.3 (0.4)	1.7 (0.4)	-0.4 (0.5)	2.2 (0.4)	2.9 (0.5)	-0.8 (0.5)	2.3 (0.4)	3.3 (0.4)	-1.0 (0.5)	1.8 (0.2)	2.7 (0.2)	-0.9 (0.3)
Month 3	0.4 (0.3)	0.8 (0.2)	-0.4 (0.3)	1.4 (0.4)	1.9 (0.4)	-0.5 (0.5)	0.9 (0.3)	1.4 (0.3)	-0.4 (0.4)	0.8 (0.2)	1.4 (0.2)	-0.6 (0.2)
**Pain at Rest**												
Month 1	1.5 (0.4)	2.3 (0.4)	-0.8 (0.5)	2.4 (0.5)	3.4 (0.5)	-1.0 (0.6)	2.3 (0.3)	2.9 (0.4)	-0.6 (0.5)	2.0 (0.2)	2.9 (0.2)	-0.9 (0.3)
Month 3	0.8 (0.3)	1.3 (0.3)	-0.5 (0.4)	1.6 (0.5)	2.1 (0.5)	-0.5 (0.5)	1.5 (0.3)	1.5 (0.3)	-0.05 (0.4)	1.3 (0.2)	1.7 (0.2)	-0.5 (0.3)
**Pain with Movement**												
Month 1	2.0 (0.4)	2.6 (0.4)	-0.5 (0.6)	2.9 (0.6)	4.2 (0.6)	-1.3 (0.7)	3.1 (0.4)	4.3 (0.5)	-1.2 (0.6)	2.6 (0.3)	3.7 (0.3)	-1.1 (0.4)
Month 3	1.1 (0.4)	1.4 (0.3)	-0.3 (0.4)	1.8 (0.5)	2.4 (0.6)	-0.6 (0.6)	1.9 (0.4)	2.2 (0.5)	-0.3 (0.6)	1.5 (0.2)	2.1 (0.2)	-0.5 (0.3)

© 2026 Abraham J et al. *JAMA Network Open.*

eTable 6. Impact of intervention on falls

	Cardiac		Difference Between Groups	Oncologic		Difference Between Groups	Orthopedic		Difference Between Groups	Pooled		Difference Between Groups
**Monthly Number of Falls, LSM (SE)**	Intervention	EUC		Intervention	EUC		Intervention	EUC		Intervention	EUC	
**Month 1**	0.13 (0.08)	0.30 (0.08)	-0.17 (0.12)[a]	0.10 (0.05)	0.13 (0.06)	-0.03 (0.07)[a]	0.12 (0.07)	0.12 (0.07)	-0.002 (0.10)[a]	0.11 (0.04)	0.18 (0.04)	-0.07 (0.06)[a]
**Month 3**	0.20 (0.05)	0.20 (0.05)	0.005 (0.08)[b]	0.22 (0.06)	0.18 (0.07)	0.04 (0.08)[b]	0.08 (0.03)	0.14 (0.04)	-0.07 (0.05)[b]	0.16 (0.03)	0.17 (0.03)	-0.02 (0.04)[b]
**Severity of Falls, Raw Means (SD)**												
**Month 1**	0.2 (0.8)	0.3 (0.9)	P = 0.2[c]	0.1 (0.4)	0.1 (0.4)	P = 0.7[c]	0.4 (1.2)	0.2 (0.7)	P = 0.1[c]	0.2 (0.8)	0.2 (0.7)	P = 0.1[c]
**Month 3**	0.4 (0.9)	1.2 (2.0)		0.5 (1.2)	0.3 (0.9)		0.2 (0.7)	0.5 (1.0)		0.4 (0.9)	0.7 (1.4)	

[a]The contrast of interest is $INT_{M1} – UC_{M1}$.
[b]The contrast of interest is $INT_{M3} – UC_{M3}$.
[c]The P-value represents the group effect.

© 2026 Abraham J et al. *JAMA Network Open.*

eTable 7. Impact of intervention on length of hospital stay and length of ICU stay

**Median (25th, 75th) Percentile**	**Cardiac (n = 102)**		**Oncology (n = 100)**		**Orthopedics (n = 104)**		**Pooled (N = 306)**	
	Intervention (n = 51)	EUC (n = 51)	Intervention (n = 52)	EUC (n = 48)	Intervention (n = 50)	EUC (n = 54)	Intervention (n=153)	EUC (n=153)
**Length of Hospital Stay**	129.0 (31.0, 272.0)	194.0 (32.0, 361.0)	28.5 (9.3, 82.5)	30.5 (11.3, 71.5)	29.0 (27.0, 32.3)	27.5 (25.0, 31.0)	31.0 (26.0, 117.0)	31.0 (26.0, 150.5)
**Length of ICU Stay**	22.0 (0.0, 74.0)	24.0 (0.0, 94.0)	0.0 (0.0, 0.0)	0.0 (0.0, 0.0)	0.0 (0.0, 0.0)	0.0 (0.0, 0.0)	0.0 (0.0, 0.0)	0.0 (0.0, 0.0)

© 2026 Abraham J et al. *JAMA Network Open*.

eFigure 2. Intervention reach

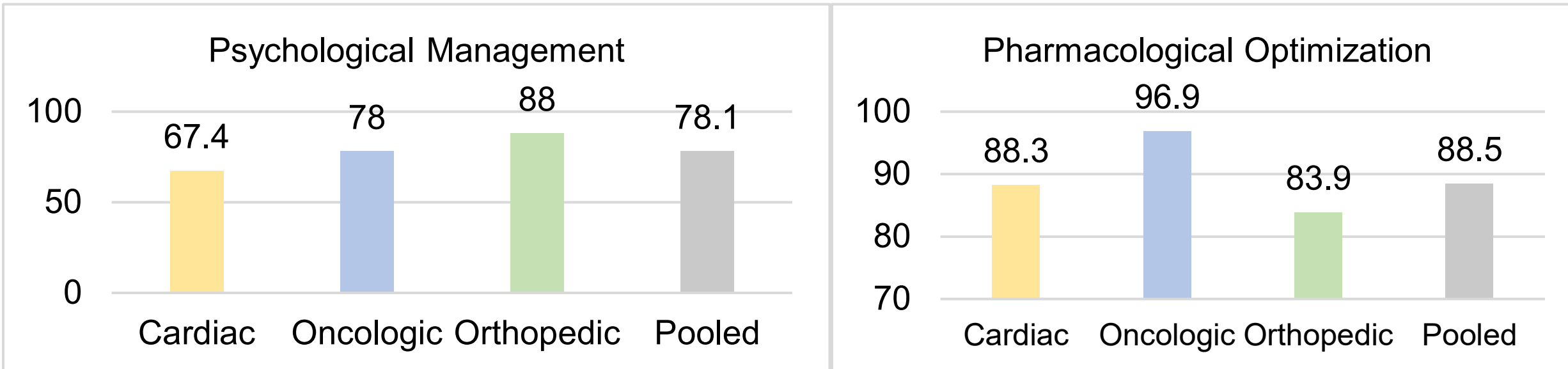

© 2026 Abraham J et al. *JAMA Network Open.*

eTable 8. Psychological management reach details

	Psychological management reach (% ≥7 sessions)	Average sessions per patient	Breakdown (Pre-op / Post-op)	Average duration (min/session)
**Cardiac**	67.4%	8.5	1.5 / 7.0	42.1
**Oncologic**	78.0%	8.7	1.2 / 7.5	43.2
**Orthopedic**	88.0%	9.0	1.7 / 7.3	41.9
**Pooled**	78.1%	8.8	1.5 / 7.3	42.4

eTable 9. Pharmacological optimization reach details

	Pharmacological optimization reach	# of patients eligible for pharmacological optimization intervention	# of patients accepting pharmacological optimization intervention
**Cardiac**	83.3%	24	20
**Oncologic**	96.9%	32	31
**Orthopedic**	83.9%	31	26
**Pooled**	88.5%	87	77

eFigure 3. Acceptability, appropriateness, and feasibility of psychological management

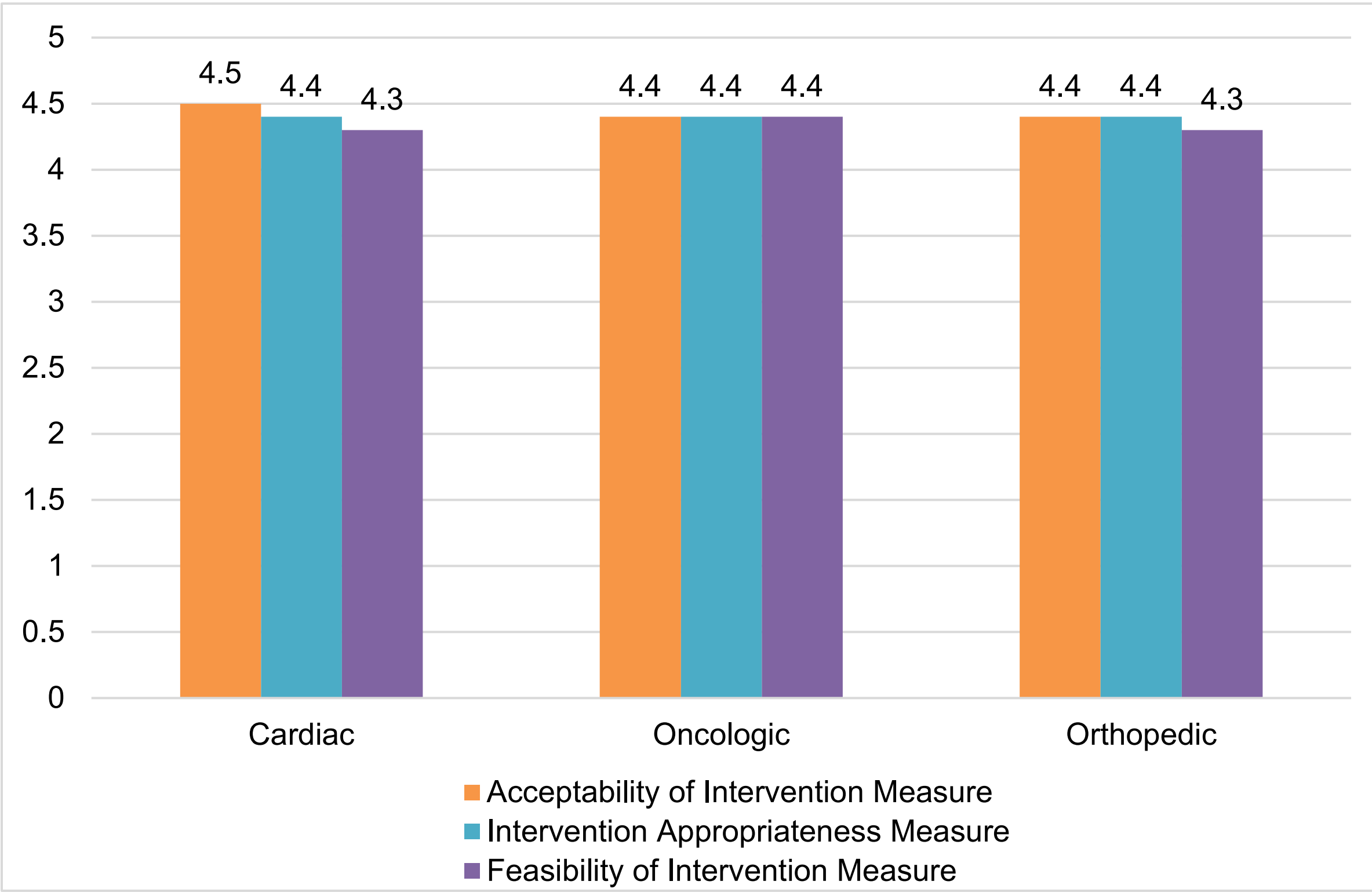

© 2026 Abraham J et al. *JAMA Network Open*.

eFigure 4. Acceptability, appropriateness, and feasibility of pharmacological optimization

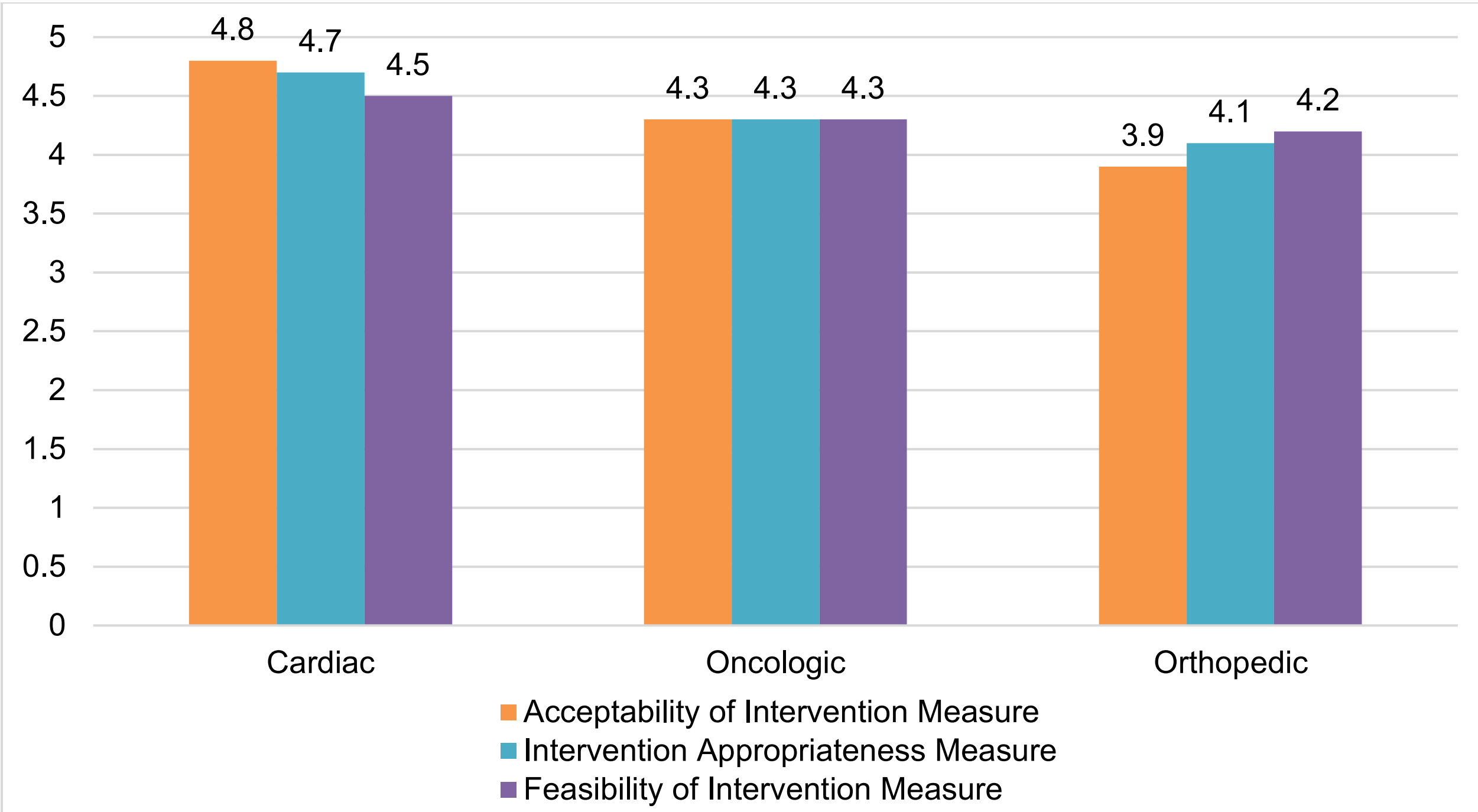

eTable 10. Patient satisfaction with psychological management

**Before beginning your session with your wellness partner, did your partner give you all the information you needed about the behavioral change component? (n = 112)**	0.0% (Lower Proportion: No)	6.2% (Middle Proportion: Yes, somewhat)	93.8% (Top Box Score: Yes, definitely)
**Before beginning your session with your wellness partner, did your partner provide you with easy-to-understand information about the behavioral change component? (n = 112)**	0.0% (Lower Proportion: No)	3.6% (Middle Proportion: Yes, somewhat)	96.4% (Top Box Score: Yes, definitely)
**During your sessions, did your partner explain what to expect during the behavioral activities? (n = 111)**	0.9% (Lower Proportion: No)	22.5% (Middle Proportion: Yes, somewhat)	76.6% (Top Box Score: Yes, definitely)
**During your sessions, did your partner listen carefully to you? (n = 112)**	0.0% (Lower Proportion: No)	0.0% (Middle Proportion: Yes, somewhat)	100.0% (Top Box Score: Yes, definitely)
**During your sessions, did your partner spend enough time with you? (n = 112)**	0.0% (Lower Proportion: No)	0.0% (Middle Proportion: Yes, somewhat)	100.0% (Top Box Score: Yes, definitely)
**During your sessions, did your partner encourage you to ask questions? (n = 112)**	0.0% (Lower Proportion: No)	3.6% (Middle Proportion: Yes, somewhat)	96.4% (Top Box Score: Yes, definitely)
**During your sessions, did your partner show respect for what you had to say? (n = 112)**	0.0% (Lower Proportion: No)	0.9% (Middle Proportion: Yes, somewhat)	99.1% (Top Box Score: Yes, definitely)
**Did the sessions make you feel more calm and relaxed? (n = 112)**	2.7% (Lower Proportion: No)	15.2% (Middle Proportion: Yes, somewhat)	82.1% (Top Box Score: Yes, definitely)
**Composite Proportional Score**	0.4%	6.5%	93.0%
**During your sessions, did your partner talk with you about the reasons you might want to participate in the behavioral activities? (n = 111)**	4.5% (Lower Proportion: Not at all/A little)	18.0% (Middle Proportion: Some)	77.5% (Top Box Score: A lot)

**During your sessions with your wellness partner, did your partner ask which behavior changes you thought would be best for you? (n = 110)**	0.9%<br>(Lower Proportion: No)	------	99.1%<br>(Top Box Score: Yes)
**During your sessions with your wellness partner, did your partner tell you there was more than one way to improve your mental health/well-being perioperatively? (n = 104)**	0.0%<br>(Lower Proportion: No)	------	100.0%<br>(Top Box Score: Yes)
**Composite Proportional Score**	0.4%	------	99.6%
**Using any number from 0-10, where 0- is the worst partner possible and 10 is the best partner possible, what number would you use to rate the sessions with your partner? (n = 112)**	3.6%<br>(Lower Proportion: 0-6)	8.0%<br>(Middle Proportion: 7-8)	88.4%<br>(Top Box Score: 9-10)
**During your sessions, did your partner warm you about any signs or symptoms that would need immediate medical attention due to any behavioral activities/changes? (n = 77)**	16.9%<br>(Lower Proportion: No)	22.1%<br>(Middle Proportion: Yes, somewhat)	61.0%<br>(Top Box Score: Yes, definitely)

© 2026 Abraham J et al. *JAMA Network Open.*

eTable 11. Patient satisfaction with pharmacological optimization

**Before beginning your session with your wellness partner/pharmacy team, did they give you all the information you needed about medication review activities? (n = 49)**	4.1% (Lower Proportion: No)	22.4% (Middle Proportion: Yes, somewhat)	73.5% (Top Box Score: Yes, definitely)
**Before beginning your session with your wellness partner/pharmacy team, did they provide you with easy-to-understand information about the medication review? (n = 50)**	2.0% (Lower Proportion: No)	12.0% (Middle Proportion: Yes, somewhat)	86.0% (Top Box Score: Yes, definitely)
**During your sessions, did your wellness partner/pharmacy team explain what to expect during your medication review? (n = 51)**	2.0% (Lower Proportion: No)	27.5% (Middle Proportion: Yes, somewhat)	70.6% (Top Box Score: Yes, definitely)
**During your sessions, did your wellness partner/pharmacist warn you about any signs or symptoms that would need immediate medical attention due to any medication changes? (n = 48)**	27.1% (Lower Proportion: No)	25.0% (Middle Proportion: Yes, somewhat)	47.9% (Top Box Score: Yes, definitely)
**Composite Proportional Score**	8.8%	21.7%	69.5%
**During your sessions, did your wellness partner/pharmacy team talk with you about the reasons you might want to make changes to your medications? (n = 51)**	7.8% (Lower Proportion: Not at all/A little)	25.5% (Middle Proportion: Some)	66.7% (Top Box Score: A lot)
**During your sessions with your wellness partner/pharmacy team, did they ask which medication would be best for you? (n = 43)**	32.6% (Lower Proportion: No)	----------	67.4% (Top Box Score: Yes)
**During your pharmacy sessions, did your pharmacist listen carefully to you? (n = 38)**	2.6% (Lower Proportion: No)	5.3% (Middle Proportion: Yes, somewhat)	92.1% (Top Box Score: Yes, definitely)

© 2026 Abraham J et al. *JAMA Network Open*.

**During your pharmacy sessions, did your pharmacist spend enough time with you? (n =38)**	0.0%<br>(Lower Proportion: No)	0.0%<br>(Middle Proportion: Yes, somewhat)	100.0%<br>(Top Box Score: Yes, definitely)
**During your pharmacy sessions, did your pharmacist encourage you to ask questions? (n = 38)**	0.0%<br>(Lower Proportion: No)	7.9%<br>(Middle Proportion: Yes, somewhat)	92.1%<br>(Top Box Score: Yes, definitely)
**During your pharmacy sessions, did your pharmacist show repsect for what you had to say? (n = 38)**	2.6%<br>(Lower Proportion: No)	2.6%<br>(Middle Proportion: Yes, somewhat)	94.7%<br>(Top Box Score: Yes, definitely)
**Did the pharmacy sessions make you feel more calm and relaxed? (n = 38)**	7.9%<br>(Lower Proportion: No)	10.5%<br>(Middle Proportion: Yes, somewhat)	81.6%<br>(Top Box Score: Yes, definitely)
**Composite Proportional Score**	2.6%	5.3%	92.1%
**During your pharmacy sessions, did your pharmacist tell you there was more than one way (alternate medications) to improve your mental health/well-being perioperatively? (n = 37)**	29.7%<br>(Lower Proportion: No)	------	70.3%<br>(Top Box Score: Yes)
**Using any number from 0-10, where 0 is the worst pharmacist possible and 10 is the best pharmacist possible, what number would you use to rate the sessions with your pharmacist? (n = 42)**	11.9%<br>(Lower Proportion: 0-6)	7.1%<br>(Middle Proportion: 7-8)	81.0%<br>(Top Box Score: 9-10)

eTable 12. Intervention delivery process measures

Intervention Components	Cardiac	Oncologic	Orthopedic	Pooled
**Psychological Management**				
Average Psychological Management Sessions	6.5 (3.0)	7.3 (2.8)	8.4 (2.3)	7.4 (2.8)
Psychological Management Preoperative Sessions	1.2 (1.2)	1.0 (1.4)	1.5 (1.5)	1.2 (1.4)
Psychological Management Postoperative Sessions	5.3 (2.8)	6.3 (2.4)	6.8 (1.7)	6.2 (2.4)
Psychological Management Session Duration (Minutes)	41.7 (19.5)	42.9 (17.0)	42.6 (16.7)	42.4 (17.6)
**Pharmacological Optimization**				
Average Pharmacological Optimization Sessions	2.3 (1.4)	2.2 (1.4)	2.2 (1.2)	2.2 (1.5)
Pharmacological Optimization Preoperative Sessions	1.3 (0.8)	1.1 (0.3)	1.3 (0.8)	1.2 (0.7)
Pharmacological Optimization Postoperative Sessions	1.7 (1.1)	1.9 (1.5)	1.7 (1.3)	1.8 (1.3)
Pharmacological Optimization Session Duration (Minutes)	17.2 (9.2)	17.1 (9.4)	17.6 (10.1)	17.3 (9.6)

© 2026 Abraham J et al. *JAMA Network Open*.

eTable 13. Summary of planned and completed psychological management activities[a]

	Cardiac (N=44)		Oncologic (N=46)		Orthopedic (N=52)	
	Planned	Completed	Planned	Completed	Planned	Completed
Exercise	33	28	37	33	43	43
Mindfulness	26	15	33	19	32	18
Improve sleep	24	8	26	20	32	24
Engage in brain training activities	19	13	21	17	23	12
Invest in hobbies and interests	20	19	20	20	33	26
Socialize with friends, family, and community	20	20	25	23	28	25
Do chores	23	22	32	30	31	28
Contact care team about health concerns	14	10	10	5	10	5
Prepare for surgery	4	4	1	1	7	7
Make diet changes	5	4	10	10	0	0
Self-care	11	9	10	9	8	6
Focus on work	3	3	3	3	7	6
Create routine	1	0	15	10	6	5
Quit unhealthy habits	2	1	2	2	3	1

	Cardiac (N=44)		Oncologic (N=46)		Orthopedic (N=52)	
	**Planned**	**Completed**	**Planned**	**Completed**	**Planned**	**Completed**
Prepare for the future	1	1	2	1	3	1

[a]Across cohorts, the most common goals and activities that patients reported were exercise, engaging in mindfulness, investing in hobbies and interests, and improving sleep. Some patients admitted that factors such as physical recovery from surgery, feelings of being overwhelmed, and lack of consistent ability to document activities outside of sessions were challenging, but they noted that flexibility in activity and session structure helped them to stay on track with their sessions and cited compassionate, personalized care as crucial to the success of the intervention.

Completion rates for activities were highest in the oncologic cohort at 82%, while slightly lower in cardiac (76%) and orthopedic (78%) cohorts. The table provides further details on planned and completed psychological management goals across cohorts.

eTable 14. Summary of pharmacological interventions

Medication Types	Cardiac	Oncologic	Orthopedic	Pooled
Muscle Relaxants	9	28	5	42
Benzodiazepines	12	7	12	31
Anti-emetics	1	9	14	24
First Generation Antihistamines	7	4	13	24
Non-benzodiazepine Z-drugs	4	4	4	12
Anticholinergics	3	2	1	6
Tricyclic Antidepressants	1	1	4	6
**Total**	**37**	**55**	**53**	**145**

eTable 15. Medication types within pharmacological optimization sessions

	Cardiac	Oncologic	Orthopedic	Pooled
**De-prescribing**				
PIMs Eligible for De-Prescribing<br>Out-of-Hospital<br>In-Hospital	113<br>36<br>77 (53/77 were one-time orders given before ability to intervene)	150<br>40<br>110 (85/110 were one-time orders given before ability to intervene)	187<br>33<br>154 (127/154 were one-time orders given before ability to intervene)	450<br>109<br>341 (265/341 were one-time orders given before ability to intervene)
Proposed Deprescribing	40	53	54	147
Accepted & Implemented Deprescribing	30	49	42	121
**Antidepressant Dose Escalation**				
Home Antidepressants Eligible	2	3	2	7
Proposed Dose Escalation	1	3	1	5
Accepted & Implemented Dose Escalation	1	2	1	4

eTable 16. Intervention implementation fidelity to psychological management and pharmacological optimization

Intervention Adherence %	Cardiac (N=20)	Oncologic (N=20)	Orthopedic (N=20)	Pooled (N=60)
**Psychological Management**				
Psychological Management Treatment Delivery	98.60%	99.30%	98.60%	98.80%
Psychological Management Treatment Receipt	100.00%	100.00%	97.50%	99.20%
Psychological Management Enactment: Activity Completion	95.00%	100.00%	95.00%	96.70%
Psychological Management Enactment: Progress Recording	55.00%	75.00%	50.00%	76.70%
**Pharmacological Optimization**				
Pharmacological Optimization Treatment Delivery	79.40%	62.90%	86.90%	78.20%
Pharmacological Optimization Treatment Receipt	86.40%	86.40%	62.50%	71.90%
Pharmacological Optimization Enactment: Activity Completion	88.30%	87.50%	90.50%	89.40%
Pharmacological Optimization Enactment: Progress Recording	79.40%	62.90%	86.90%	78.20%

© 2026 Abraham J et al. *JAMA Network Open.*

eTable 17. Patient and interventionist experiences with the intervention[16]

**Benefits of Intervention**	*Collaboration between outpatient care team and Interventionists*	*"[MO was] basically reconfirming that what I'm doing and taking is proper for me right now and there is only one change of trying to delete one medication. During this process, my cardiologist has changed my medication quite frequently and your people have concurred with what was being done."* – Cardiac Patient 1394
	Compassion and support from interventionists	*"You feel like you had a coach. Someone who could listen, somebody who is outside the family and friends who is focused and understanding and knowledgeable about the process. My experience was very positive… and it helped me quite a bit in my recovery. My mental part of it, in particular. [Psychological management] kept me positive and [my wellness partner] encourage [me] – it was great… She helped me to stay on track, stay focused, and realize it's gonna take time but I just had to put in the time and effort and thought to moving ahead. I found it very helpful." – Cardiac Patient 1530*<br><br>*"You start to get a little anxiety a couple days before you get the CAT scan… You know, there's a lot of anxiety… You're not sure how bad it's gonna be, you're not sure if you're gonna have to go back on chemo so you know, it's kind of good to have someone to talk to, to just kind of to be levelheaded and kind of open-minded about things… I feel like [activity planning… helps you just kind of focus your mind and concentrate and relax." – Oncologic Patient 1050*<br><br>*"I would say, to some extent, [integrating compassionate care] comes automatically. Because you're dealing with these people (that are) opening up to you… They're not feeling good… So, instead of saying, 'Okay, I'm here to do my session, let's schedule activities…' You take time to listen to them, make sure they're okay… [Understand] what they're going through is hard and trying to talk to them to help them out." – Wellness Partner 2, Focus Group*
	PMH intervention flexibility and personalization	*"A couple times… we bumped into scheduling conflicts and just moved it around… That kind of flexibility was really good, because people have messes that they got to clean up so the flexibility aspect of that really helps." – Cardiac Patient 1699*<br><br>*"I know this is all for post-surgery and stuff, but being that I had underlying illness and blah blah, you know, it's trying to find your new way of life. 'Cause, you know, surgery's for a specific period of time, and then you're basically expected to recover have, you know, something happen afterwards. So, that was helpful to me to help me kind of set some goals that didn't have to do with, you know, cleaning my house… [My goals] have to be more, like, personal." (Oncologic Patient 1069)*
	Cohort-specific medication support	*"I think it got me to actually think about… the additional medications that I'd been asked to take… It actually [led me to question]… what other medications, since I've been going through this, you know, chemotherapy and surgery and stuff like that this past six months, to think about what I'm taking in. And as the future goes on to think about additionally what I'm gonna be taking in as well." – Oncologic Patient 1299*

		"A lot of time it's like pain and sleep; those are mostly the [medications that patients have]." – Pharmacist 5, Focus Group
**Limitations to Intervention**	*Less effective intervention for simpler surgeries*	*"I think if the surgery had been more complicated as far as healing goes it might have been a little – it might've, I don't know…People with more complicated healing surgeries might benefit even more than I did because my healing process was very easy." – Cardiac Patient 2049*
	Less effective pharmacological component for some cohorts	*"I think [the psychological management was helpful]. Not so much the physical part of it, because that falls on the physical therapist and whether or not I'm willing to do the work, but the mental and emotional part of it was very helpful, I thought." – Orthopedic Patient 1344*<br><br>*"With ortho, a lot of the [psychological management activities] were no longer available for them because of pain. So, [we made an effort to] focus on things that they would like to do again." – Wellness Partner 1, Focus Group*
	Difficulty in completing activities for some patients	*"Well, just the activities. I found them somewhat overwhelming. That's why I didn't follow through and do a lot of it…I was just focused on, uh, just trying to, just recover and do my day-to-day activities… I was just overwhelmed with the increased activity."* – Orthopedic Patient 1074
	Accessibility concerns based on patient technological literacy	*"I think the main difference (between Zoom and phone) would be accessibility. I think this particular patient population has a very difficult time signing on... any Zoom meeting I've had has been late by at least 15 min, because of patient difficulty in navigating the. IT environment... I have better patient interactions (over the telephone)." – Pharmacist 5, Focus Group*

eTable 18. Target engagement survey scores

Survey Mean Score (SD)	Cardiac (n=40)	Oncologic (n=46)	Orthopedic (n=48)
Behavioral Activation for Depression (BADS) activation subscale	4.2 (0.7)	4.3 (0.5)	4.2 (0.5)
Sinclair Compassion Questionnaire	4.8 (0.4) (n=39)	4.7 (0.7)	4.9 (0.3) (n=46)
MiPrep Survey for Patient Preparation	4.6 (0.5)	4.4 (0.5) (n=45)	4.6 (0.6) (n=47)
"Speaking with a pharmacist helped me understand that certain medications have the potential to affect my mental health."	3.9 (1.1) (n=36)	4.1 (1.1) (n=42)	4.1 (1.0) (n=42)

© 2026 Abraham J et al. *JAMA Network Open*.

References

1. Panel AGSBCUE. American Geriatrics Society 2023 updated AGS Beers Criteria® for potentially inappropriate medication use in older adults. *Journal of the American Geriatrics Society*. 2023;71(7):2052-2081.

2. Boehlen FH, Freigofas J, Herzog W, et al. Evidence for underuse and overuse of antidepressants in older adults: Results of a large population-based study. *Int J Geriatr Psychiatry*. Apr 2019;34(4):539-547. doi:10.1002/gps.5047

3. Desseilles M, Witte J, Chang TE, et al. Assessing the adequacy of past antidepressant trials: a clinician's guide to the antidepressant treatment response questionnaire. *J Clin Psychiatry*. Aug 2011;72(8):1152-4. doi:10.4088/JCP.11ac07225

4. Sackeim HA, Aaronson ST, Bunker MT, et al. The assessment of resistance to antidepressant treatment: Rationale for the Antidepressant Treatment History Form: Short Form (ATHF-SF). *J Psychiatr Res*. Jun 2019;113:125-136. doi:10.1016/j.jpsychires.2019.03.021

5. Taylor WD. Clinical practice. Depression in the elderly. *N Engl J Med*. Sep 25 2014;371(13):1228-36. doi:10.1056/NEJMcp1402180

6. Bellg AJ, Borrelli B, Resnick B, et al. Enhancing treatment fidelity in health behavior change studies: best practices and recommendations from the NIH Behavior Change Consortium. *Health Psychol*. 2004;23(5):443.

7. Borrelli B, Sepinwall D, Ernst D, et al. A new tool to assess treatment fidelity and evaluation of treatment fidelity across 10 years of health behavior research. *J Consult Clin Psychol*. 2005;73(5):852.

8. Breimaier HE, Heckemann B, Halfens RJ, Lohrmann C. The Consolidated Framework for Implementation Research (CFIR): a useful theoretical framework for guiding and evaluating a guideline implementation process in a hospital-based nursing practice. *BMC Nurs*. 2015;14:43.

9. Finley EP, Huynh AK, Farmer MM, et al. Periodic reflections: a method of guided discussions for documenting implementation phenomena. *BMC medical research methodology*. 2018;18(1):153.

10. Sinclair S, Hack TF, MacInnis CC, et al. Development and validation of a patient-reported measure of compassion in healthcare: the Sinclair Compassion Questionnaire (SCQ). *BMJ Open*. 2021;11(6):e045988.

11. Kanter JW, Mulick PS, Busch AM, Berlin KS, Martell CR. The Behavioral Activation for Depression Scale (BADS): psychometric properties and factor structure. *Journal of Psychopathology and Behavioral Assessment*. 2007;29(3):191-202.

12. Fakes KL, Hall AE, Carey ML, et al. Development and validation of the MiPrep survey: an instrument assessing patients' perceived preparation for medical interventions including medical imaging, radiotherapy, and surgery. *Value in Health*. 2019;22(6):704-711.

13. Alexopoulos GS, Raue PJ, Gunning F, et al. “Engage” therapy: behavioral activation and improvement of late-life major depression. *The American Journal of Geriatric Psychiatry*. 2016;24(4):320-326.

14. Clarke V, Braun V. Thematic analysis. In: Teo T, ed. *Encyclopedia of Critical Psychology*. Springer; 2014:1947-1952.

15. George A. Quantitative and qualitative approaches to content analysis. In: Krippendorff K, Bock M, eds. *The Content Analysis Reader*. Sage Publications; 2009:144-155.

16. Abraham J, Wang J, Meng A, et al. Integrating mental health into surgical care: a qualitative study of a perioperative mental health intervention. *Ann Surg Open*. 2026;7(2):e675.

© 2026 Abraham J et al. *JAMA Network Open.*
