# Supplementary material for: Perioperative Depression and Anxiety Care in Older Patients: A Randomized Clinical Trial
Source: JAMA Netw Open. 2026 Sep 9;9(9):e2632619. doi: 10.1001/jamanetworkopen.2026.32619 (PMC13559599; doi:10.1001/jamanetworkopen.2026.32619)
Supplement: Supplementary file 3 [file jamanetwopen-e2632619-s003.pdf]

Data Sharing Statement

Abraham. Perioperative Depression and Anxiety Care in Older Patients. *JAMA Netw Open*. Published September 09, 2026. doi:10.1001/jamanetworkopen.2026.32619

Data
Additional Information: ClinicalTrials.gov Identifier: NCT05575128 (Behavioral Activation and Medication Optimization In Older Adults Undergoing Cardiac Procedures https://clinicaltrials.gov/study/NCT05575128), NCT05685511 (Behavioral Activation and Medication Optimization For Improving Perioperative Mental Health In Older Adults Undergoing Oncologic Surgery https://clinicaltrials.gov/study/NCT05685511), NCT05697835 Perioperative Mental Health in Orthopedic Surgery (CPMH) https://clinicaltrials.gov/study/NCT05697835)
Data available: Yes
Data types: Deidentified participant data
How to access data: joannaa@wustl.edu
When available: With publication

Supporting Documents
Document types: None

Additional Information
Who can access the data: Researchers whose proposed use of the data has been approved
Types of analyses: For a specified purpose
Mechanisms of data availability: With a signed data access agreement
